# Supplementary material for: Deciphering the Origin and Evolution of Hepatitis B Viruses by Means of a Family of Non-enveloped Fish Viruses
Source: Cell Host Microbe. 2017 Sep 13;22(3):387–399.e6. doi: 10.1016/j.chom.2017.07.019 (PMC5604429; doi:10.1016/j.chom.2017.07.019)
Supplement: Document S1. Figures S1–S7 and Table S1 [file mmc1.pdf]

Cell Host & Microbe, Volume *22*

Supplemental Information

Deciphering the Origin and Evolution of Hepatitis B Viruses by Means of a Family of Non-enveloped Fish Viruses

Chris Lauber, Stefan Seitz, Simone Mattei, Alexander Suh, Jürgen Beck, Jennifer Herstein, Jacob Börold, Walter Salzburger, Lars Kaderali, John A.G. Briggs, and Ralf Bartenschlager

SUPPLEMENTAL INFORMATION

Figure S1, related to Figure 1. Genome maps of novel HBV-related fish viruses

Figure S2, related to Figure 1. Genome maps of new tetrapod hepatitis B viruses

Figure S3, related to Figure 3. Morphology and ultrastructure of heterologously expressed nackednavirus capsids

Figure S4, related to Figure 4. Uncalibrated phylogenetic trees of P

Figure S5, related to Figure 4. Uncalibrated Bayesian phylogenetic trees of P including outgroups, and of C

Figure S6, related to Figures 5 and 6. Correlation of the hepadnaviral phylogeny with the host phylogeny

Figure S7, related to Figure 4. Phylogenetic relationship of endogenous avihepadnaviruses (eAHBV-*FRY*), time-calibrated tree based on endogenous snake hepatitis B virus 1 (eSnHBV-1), and time-calibrated subtree of HBV genotype isolates from humans and apes

Table S1, related to Figure 1. Synopsis of novel HBV-related viruses described in this study

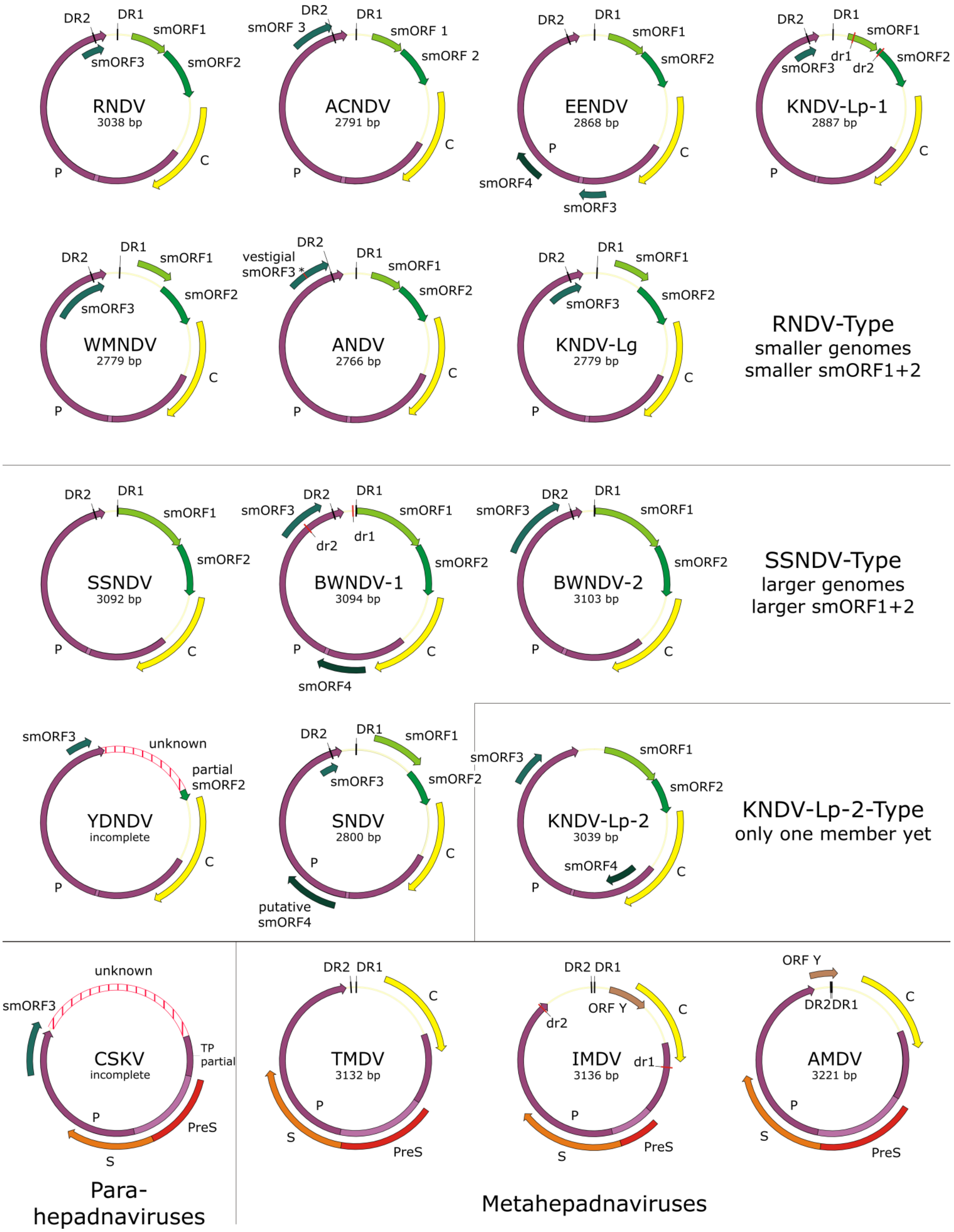

DR2
DR1
smORF1
smORF3
smORF2
RNDV
3038 bp
C
P
smORF 3
DR2
DR1
smORF 1
smORF 2
ACNDV
2791 bp
C
P
DR2
DR1
smORF1
smORF2
EENDV
2868 bp
C
P
smORF4
smORF3
DR2
DR1
smORF1
dr1
dr2
smORF2
smORF3
KNDV-Lp-1
2887 bp
C
P
DR2
DR1
smORF1
smORF2
smORF3
WMNDV
2779 bp
C
P
vestigial
smORF3 *
DR2
DR1
smORF1
smORF2
ANDV
2766 bp
C
P
DR2
DR1
smORF1
smORF2
smORF3
KNDV-Lg
2779 bp
C
P
RNDV-Type
smaller genomes
smaller smORF1+2
DR2
DR1
smORF1
smORF2
SSNDV
3092 bp
C
P
smORF3
DR2
DR1
smORF1
dr2
dr1
smORF2
BWNDV-1
3094 bp
C
P
smORF4
smORF3
DR2
DR1
smORF1
smORF2
BWNDV-2
3103 bp
C
P
SSNDV-Type
larger genomes
larger smORF1+2
smORF3
unknown
partial
smORF2
YDNDV
incomplete
C
P
DR2
DR1
smORF1
smORF3
smORF2
SNDV
2800 bp
C
P
putative
smORF4
smORF3
smORF1
smORF2
KNDV-Lp-2
3039 bp
smORF4
C
P
KNDV-Lp-2-Type
only one member yet
unknown
smORF3
TP
partial
CSKV
incomplete
P
PreS
S
Para-
hepadnaviruses
DR2 DR1
C
TMDV
3132 bp
P
S
PreS
DR2 DR1
ORF Y
C
dr2
IMDV
3136 bp
dr1
P
S
PreS
ORF Y
DR2DR1
C
AMDV
3221 bp
P
S
PreS
Metahepadnaviruses

Figure S1, related to Figure 1. Genome maps of HBV-related fish viruses

The 5'-ends of direct repeat DR1 were defined as genome start coordinates. Several viral genomes contain an additional pair of direct repeats (dr1, dr2) with uncertain functionality. None of the viruses described here has a PreC ORF typical for ortho- and avihepadnaviruses. Rows 1 to 4: nackednaviruses; row 5: hepadnaviruses. ACNDV: African cichlid nackednavirus (a fragment of the genome of this nackednavirus was previously described as ACHBV – African cichlid hepatitis B virus – by Hahn et al., 2015); AMDV: Astatotilapia metahepadnavirus; ANDV: Astatotilapia nackednavirus; BWNDV-1 and -2: “Baby whale” (Mormyrid) nackednaviruses; CSKV: Coho salmon kidney virus; EENDV: European eel nackednavirus; IMDV: Icefish metahepadnavirus; KNDV-Lg: Killifish nackednavirus from Lucania goodei; KNDV-Lp-1 and -2: Killifish nackednaviruses from Lucania parva; RNDV: Rockfish nackednavirus; SNDV: Stickleback nackednavirus; SSNDV: Sockeye salmon nackednavirus; TMDV: Tetra metahepadnavirus; WMNDV: Western mosquitofish nackednavirus; YNDV: Yellow drum nackednavirus.

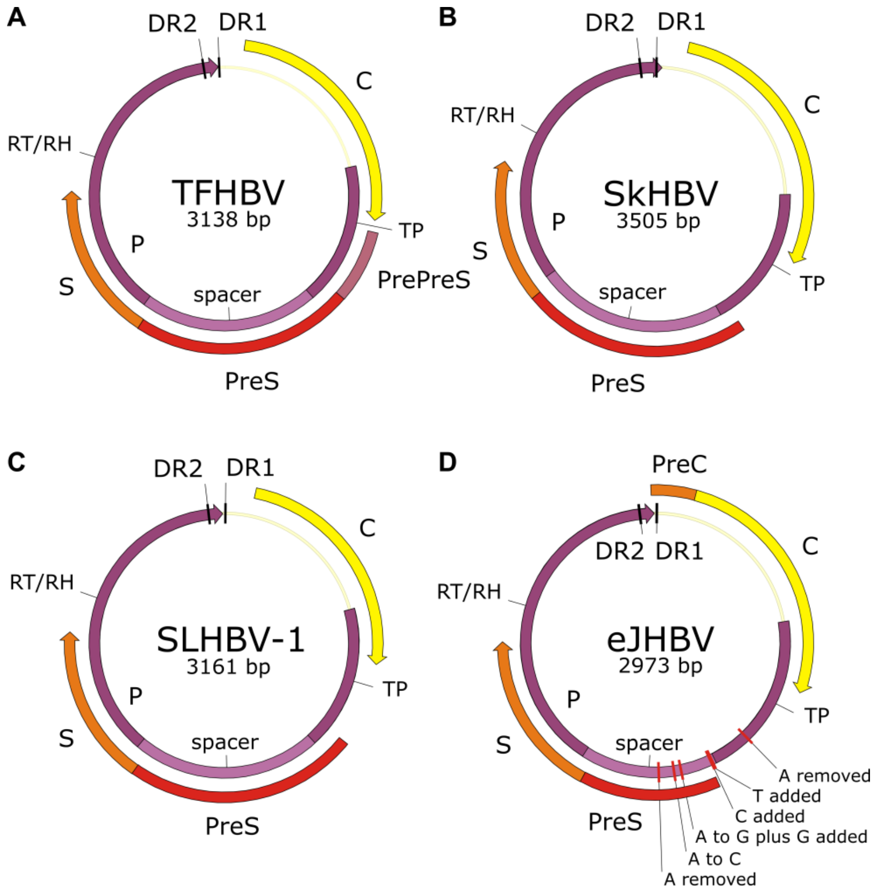

Figure S2, related to Figure 1. Genome maps of tetrapod hepatitis B viruses

(A) Tibetan frog hepatitis B virus (TFHBV). The same virus genome was independently discovered by Dill et al. (2016).

(B) Skink hepatitis B virus (SkHBV).

(C) Spiny lizard hepatitis B virus (SLHBV-1).

(D) Endogenous junco hepatitis B virus (eJHBV). Corrections for frameshift mutations and premature stop codons in eJHBV as indicated.

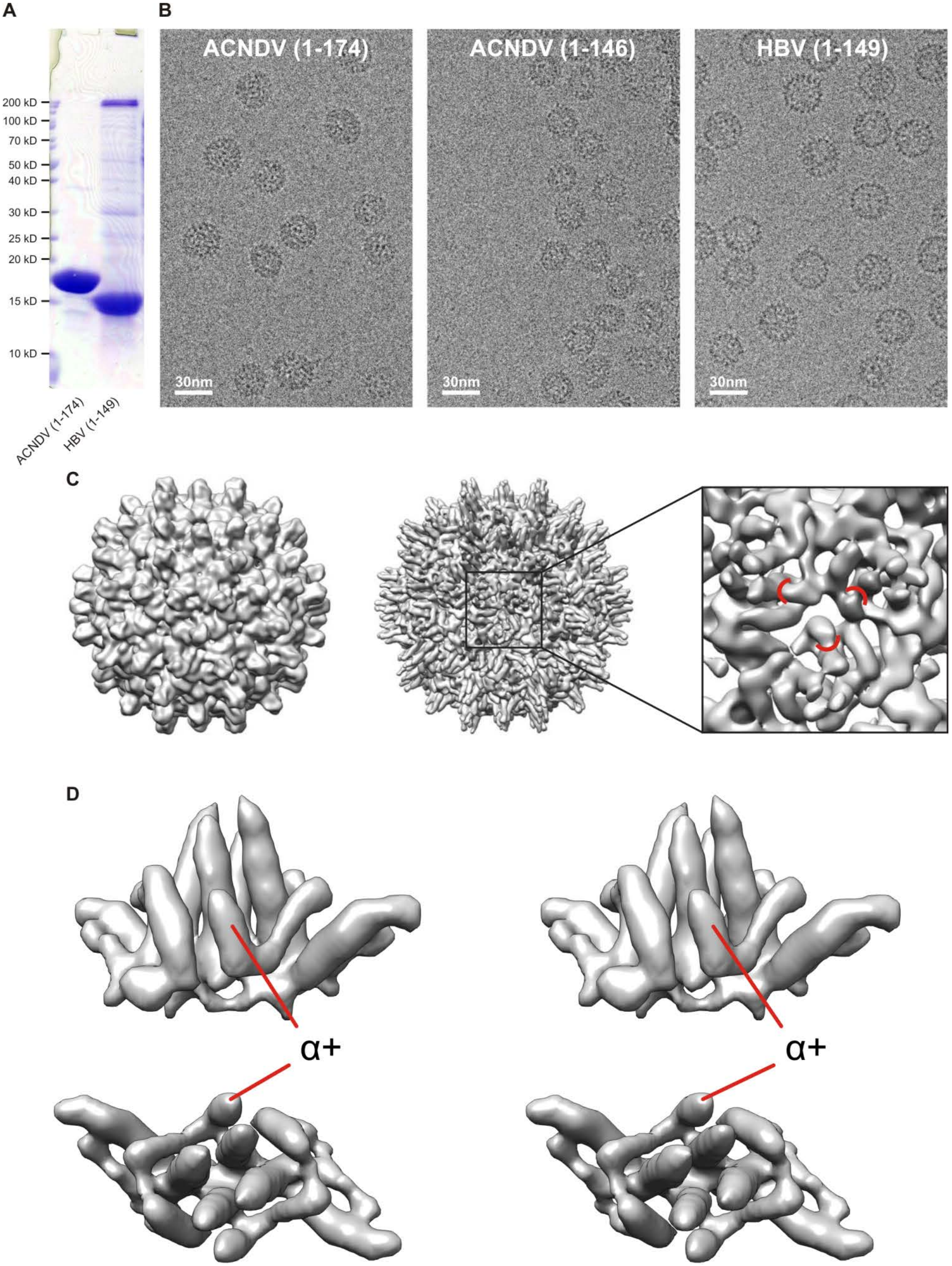

A
B
200 kD
100 kD
70 kD
50 kD
40 kD
30 kD
25 kD
20 kD
15 kD
10 kD
ACNDV (1-174)
HBV (1-149)
ACNDV (1-174)
ACNDV (1-146)
HBV (1-149)
30nm
30nm
30nm
C
D
α+
α+

Figure S3, related to Figure 3. Morphology and ultrastructure of heterologously expressed nackednavirus capsids

(A) SDS-PAGE and Coomassie-stain of highly purified ACNDV capsids consisting of full-length C protein (174 aa). Control: partially purified capsids built of truncated HBV C (aa 1-149).

(B) Cryo-electron micrographs of purified capsid particles self-assembled from full-length ACNDV C (aa 1-174), truncated ACNDV C (aa 1-146) and truncated HBV C proteins (aa 1-149), respectively. Full-length and truncated ACNDV capsids do not display the strict dimorphism known from HBV. They are more variable in shape than HBV capsid particles. The vast majority of ACNDV particles are small, and we were not able to detect a class of regular particles with T=4 icosahedral symmetry. Nonetheless, we do not rule out that some irregular particles might have local areas following a T=4 pattern. Full-length and truncated ACNDV particles appear similar, indicating that deletion of the C-terminal residues 147-174, including the nucleic acid binding domain, has no obvious influence on the shape or size distribution.

(C) Cryo-EM map of C-terminally truncated ACNDV (aa 1-146), filtered at 9 Å. Comparison with the structure of full-length ACNDV (Figure 3) indicates no substantial change in structure of the capsid scaffold. Notably, the reappearance of the additional helices sealing the holes at the local (pseudo-)three-fold axes independently corroborates that these helices represent the N-termini of ACNDV C protein molecules.

(D) Cross-eye stereo views onto an ACNDV C protein dimer. Top panel: side view; bottom panel: top view. α+: additional N-terminal α-helices not found in hepadnaviruses.

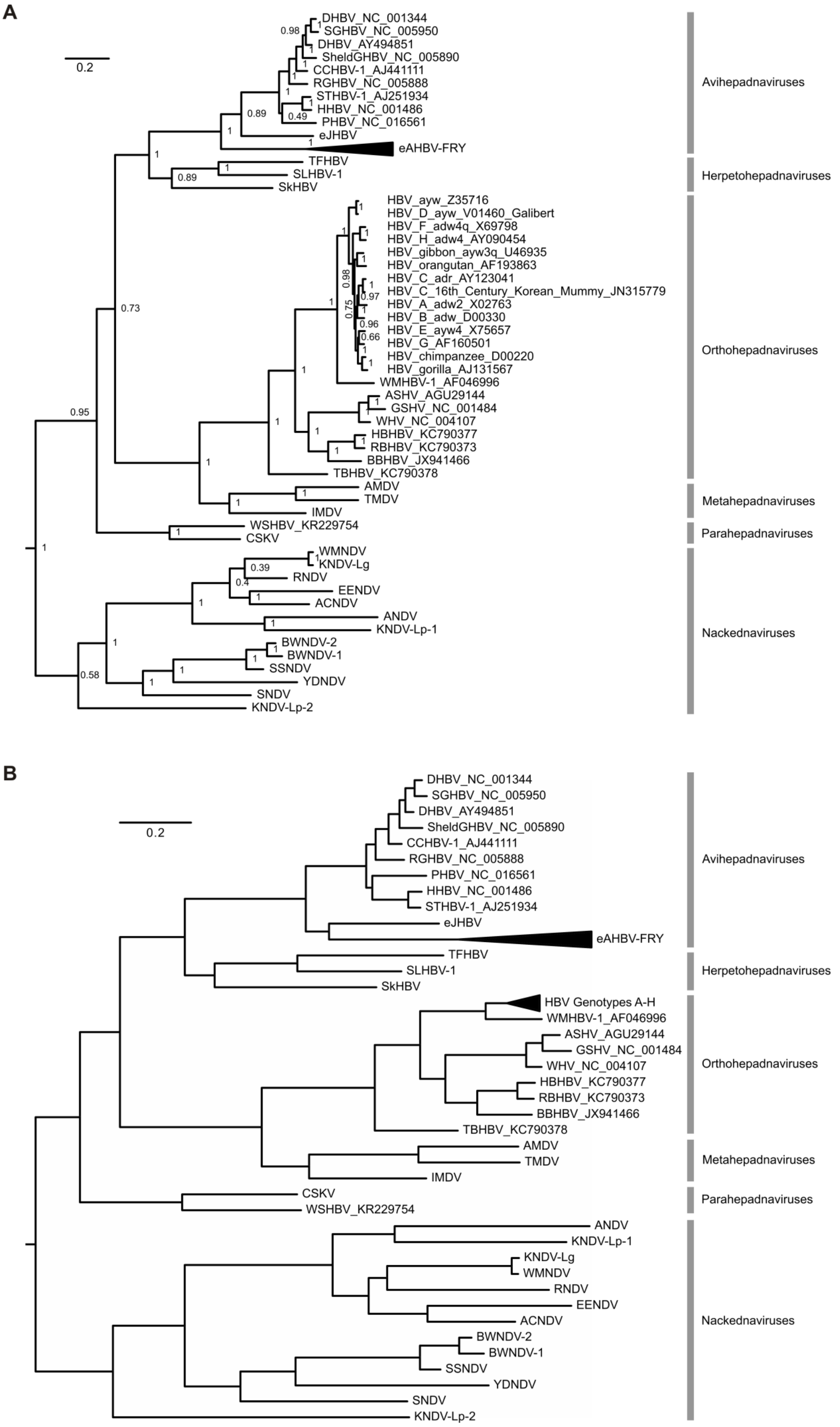
A
0.2
DHBV_NC_001344
SGHBV_NC_005950
DHBV_AY494851
SheldGHBV_NC_005890
CCHBV-1_AJ441111
RGHBV_NC_005888
STHBV-1_AJ251934
HHBV_NC_001486
PHBV_NC_016561
eJHBV
eAHBV-FRY
TFHBV
SLHBV-1
SkHBV
HBV_ayw_Z35716
HBV_D_ayw_V01460_Galibert
HBV_F_adw4q_X69798
HBV_H_adw4_AY090454
HBV_gibbon_ayw3q_U46935
HBV_orangutan_AF193863
HBV_C_adr_AY123041
HBV_C_16th_Century_Korean_Mummy_JN315779
HBV_A_adw2_X02763
HBV_B_adw_D00330
HBV_E_ayw4_X75657
HBV_G_AF160501
HBV_chimpanzee_D00220
HBV_gorilla_AJ131567
WMHBV-1_AF046996
ASHV_AGU29144
GSHV_NC_001484
WHV_NC_004107
HBHBV_KC790377
RBHBV_KC790373
BBHBV_JX941466
TBHBV_KC790378
AMDV
TMDV
IMDV
WSHBV_KR229754
CSKV
WMNDV
KNDV-Lg
RNDV
EENDV
ACNDV
ANDV
KNDV-Lp-1
BWNDV-2
BWNDV-1
SSNDV
YDNDV
SNDV
KNDV-Lp-2
Avihepadnaviruses
Herpetohepadnaviruses
Orthohepadnaviruses
Metahepadnaviruses
Parahepadnaviruses
Nackednaviruses
B
0.2
DHBV_NC_001344
SGHBV_NC_005950
DHBV_AY494851
SheldGHBV_NC_005890
CCHBV-1_AJ441111
RGHBV_NC_005888
PHBV_NC_016561
HHBV_NC_001486
STHBV-1_AJ251934
eJHBV
eAHBV-FRY
TFHBV
SLHBV-1
SkHBV
HBV Genotypes A-H
WMHBV-1_AF046996
ASHV_AGU29144
GSHV_NC_001484
WHV_NC_004107
HBHBV_KC790377
RBHBV_KC790373
BBHBV_JX941466
TBHBV_KC790378
AMDV
TMDV
IMDV
CSKV
WSHBV_KR229754
ANDV
KNDV-Lp-1
KNDV-Lg
WMNDV
RNDV
EENDV
ACNDV
BWNDV-2
BWNDV-1
SSNDV
YDNDV
SNDV
KNDV-Lp-2
Avihepadnaviruses
Herpetohepadnaviruses
Orthohepadnaviruses
Metahepadnaviruses
Parahepadnaviruses
Nackednaviruses

Figure S4, related to Figure 4. Uncalibrated phylogenetic trees of P

(A) Uncalibrated Bayesian tree based on conserved regions of the P protein (437 amino acid positions underlined with light grey bars in the P protein alignment in Supplemental Data File S3). The clade of hepatitis B virus genotypes A-H, including isolates from gibbons and great apes, is shown expanded. The eAHBV-FRY cluster is shown collapsed (see Figure S7A for an expanded subtree). Scale bar: 0.2 amino acid substitutions per site.

(B) Maximum likelihood phylogenetic tree of P. The tree is based on the same dataset as in Figures 4 and S4A. JTT + G4 + F substitution model selected by ProtTest (Abascal et al., 2005). The root was determined with TempEst (Rambaut et al., 2016b). Nackedna- and hepadnaviruses demarcate as well-separated sister clades, thus independently confirming the results of the Bayesian approach. Scale bar: 0.2 amino acid substitutions per site.

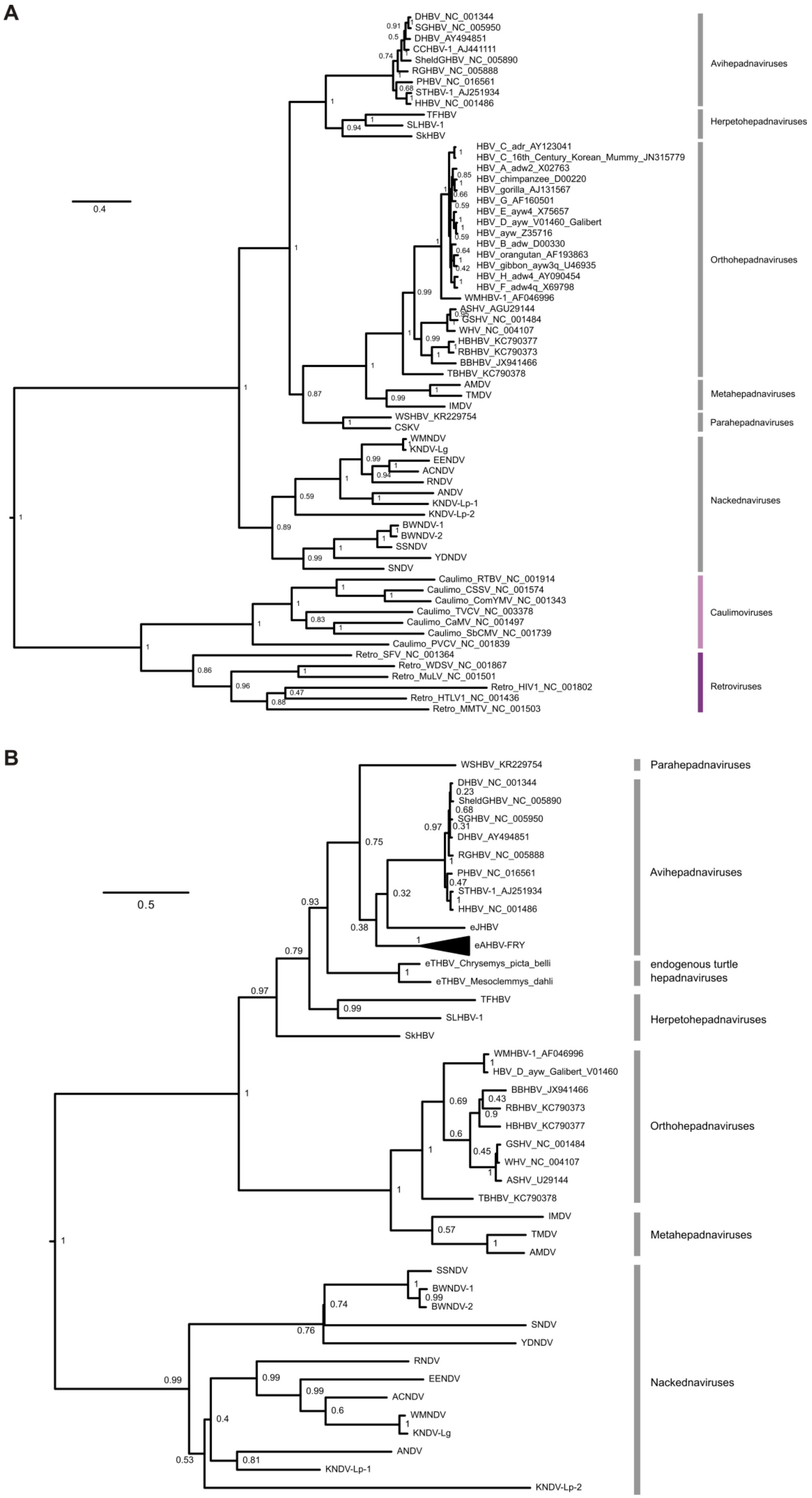
A
0.4
DHBV_NC_001344
SGHBV_NC_005950
DHBV_AY494851
CCHBV-1_AJ441111
SheldGHBV_NC_005890
RGHBV_NC_005888
PHBV_NC_016561
STHBV-1_AJ251934
HHBV_NC_001486
TFHBV
SLHBV-1
SkHBV
HBV_C_adr_AY123041
HBV_C_16th_Century_Korean_Mummy_JN315779
HBV_A_adw2_X02763
HBV_chimpanzee_D00220
HBV_gorilla_AJ131567
HBV_G_AF160501
HBV_E_ayw4_X75657
HBV_D_ayw_V01460_Galibert
HBV_ayw_Z35716
HBV_B_adw_D00330
HBV_orangutan_AF193863
HBV_gibbon_ayw3q_U46935
HBV_H_adw4_AY090454
HBV_F_adw4q_X69798
WMHBV-1_AF046996
ASHV_AGU29144
GSHV_NC_001484
WHV_NC_004107
HBHBV_KC790377
RBHBV_KC790373
BBHBV_JX941466
TBHBV_KC790378
AMDV
TMDV
IMDV
WSHBV_KR229754
CSKV
WMNDV
KNDV-Lg
EENDV
ACNDV
RNDV
ANDV
KNDV-Lp-1
KNDV-Lp-2
BWNDV-1
BWNDV-2
SSNDV
YDNDV
SNDV
Caulimo_RTBV_NC_001914
Caulimo_CSSV_NC_001574
Caulimo_ComYMV_NC_001343
Caulimo_TVCV_NC_003378
Caulimo_CaMV_NC_001497
Caulimo_SbCMV_NC_001739
Caulimo_PVCV_NC_001839
Retro_SFV_NC_001364
Retro_WDSV_NC_001867
Retro_MuLV_NC_001501
Retro_HIV1_NC_001802
Retro_HTLV1_NC_001436
Retro_MMTV_NC_001503
Avihepadnaviruses
Herpetohepadnaviruses
Orthohepadnaviruses
Metahepadnaviruses
Parahepadnaviruses
Nackednaviruses
Caulimoviruses
Retroviruses
B
0.5
WSHBV_KR229754
DHBV_NC_001344
SheldGHBV_NC_005890
SGHBV_NC_005950
DHBV_AY494851
RGHBV_NC_005888
PHBV_NC_016561
STHBV-1_AJ251934
HHBV_NC_001486
eJHBV
eAHBV-FRY
eTHBV_Chrysemys_picta_belli
eTHBV_Mesoclemmys_dahli
TFHBV
SLHBV-1
SkHBV
WMHBV-1_AF046996
HBV_D_ayw_Galibert_V01460
BBHBV_JX941466
RBHBV_KC790373
HBHBV_KC790377
GSHV_NC_001484
WHV_NC_004107
ASHV_U29144
TBHBV_KC790378
IMDV
TMDV
AMDV
SSNDV
BWNDV-1
BWNDV-2
SNDV
YDNDV
RNDV
EENDV
ACNDV
WMNDV
KNDV-Lg
ANDV
KNDV-Lp-1
KNDV-Lp-2
Parahepadnaviruses
Avihepadnaviruses
endogenous turtle hepadnaviruses
Herpetohepadnaviruses
Orthohepadnaviruses
Metahepadnaviruses
Nackednaviruses

Figure S5, related to Figure 4. Uncalibrated Bayesian phylogenetic trees of P including outgroups, and of C

(A) This P tree is based on conserved parts of RT and RH shared with caulimo- and retroviruses as outgroups (see P protein alignment in Supplemental Data File S3). Independent corroboration of the rooting of the phylogenetic inferences shown in Figures 4 and S4. Numbers at branching points show posterior probability support values. Scale bar: 0.4 amino acid substitutions per site.

(B) Bayesian phylogenetic tree of C based on 45 conserved amino acid residues of the C protein alignment shown in Supplemental Data File S4. JTT + G4 substitution model selected by ProtTest. Scale bar: 0.5 amino acid substitutions per site. The similarity of this tree with those for P (Figures 4, S4 and S5A) provides evidence that there is no significant variation of the phylogenies between genes. There is one prominent exception from the major concordance between the C- and P-based phylogenies: In the C-based tree the piscine parahepadnavirus WSHBV clusters with avihepadnaviruses, irrespective of inference method and substitution model used for tree inference. We assume this to be a case of homoplasy due to convergent evolution not reflecting the true phylogeny, since the C protein of WSHBV belongs to the plesiomorphic short type of C proteins while avi- and herpetohepadnaviruses share elongated C proteins as derived trait (Supplemental Data File S4). The other, minor differences, like the position of SkHBV, are likely caused by the limited number of conserved C protein residues resulting in a lower power to resolve isolated branches compared to the P proteins.

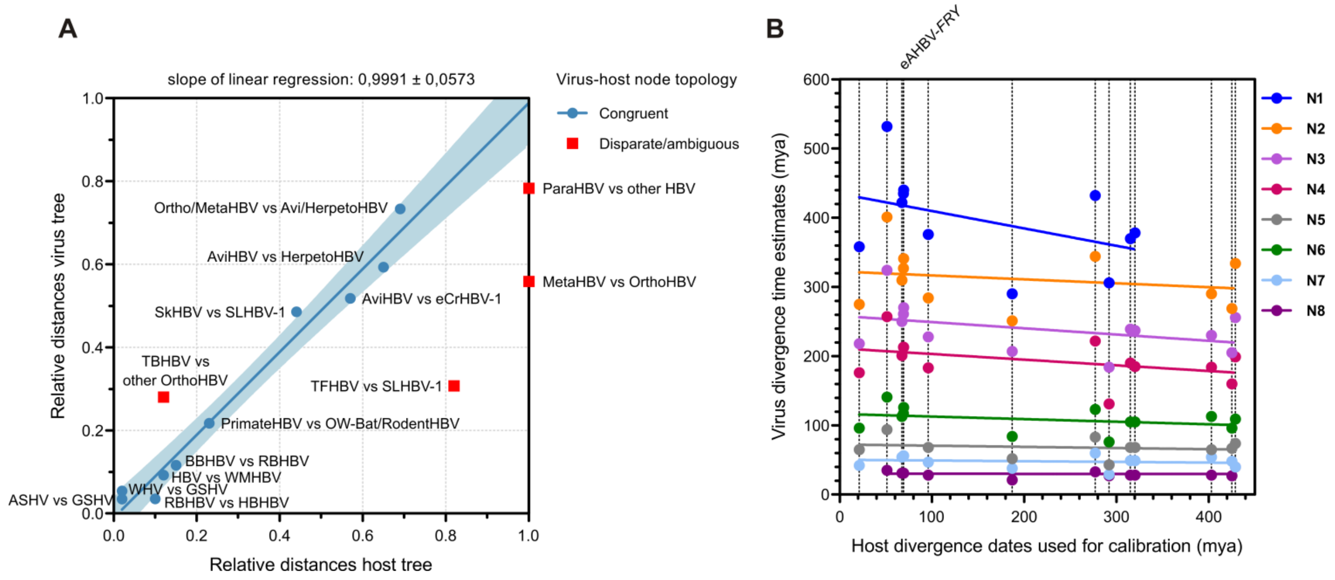

Figure S6, related to Figures 5 and 6. Correlation of the hepadnaviral phylogeny with the host phylogeny

(A) Matching of the relative distances between hepadnavirus and host nodes with congruent topology in the uncalibrated ultrametric trees shown in the tanglegram in Figure 5 (blue dots). Linear regression: $R^2 = 0.9743$. Slope = 0.9991 ± 0.0571 SD, indicating that also the roots of both trees represent a virus-host codivergence event (nackednaviral clade & actinopterygians vs. hepadnaviral clade & sarcorpterygians, including tetrapods). Red squares depict the relative distances of the major nodes with disparate virus-host topology in the tanglegram (Figure 5), as well as the one ambiguous node (piscine parahepadnaviruses vs. other hepadnaviruses) which – by mere cladistic topology – could represent an alternative cospeciation event associated with the split between actinopterygians and sarcopterygians. The evident double-mismatch of topology *and* relative branch length between the virus and host trees for these viral nodes makes it unlikely that they result from cospeciation events with their current host lineages. In order to corroborate the virus-host evolutionary patterns independently, we additionally performed statistical cophylogeny tests using ParaFit (Legendre

et al., 2002) and Jane4 (Conow et al., 2010). Based on the cladistic topology, global ParaFit *p*-values – indicating the probability of random virus-host associations, i.e. independent evolution of hosts and viruses – were <0.0001, 0.0015, and 0.3032, respectively, for the whole virus taxon sampling, hepadnaviruses only, and nackednaviruses only. When taking into account the relative branch lengths of the virus and host trees, the global ParaFit *p*-values were <0.0001 for the whole virus taxon sampling, 0.0133 for hepadnaviruses only, and 0.4025 for nackednaviruses only. Based on the cladistic topology, Jane4 predicted on average 3.54 of 12 speciation events in the nackednaviral lineage to result from virus-host cospeciation (frequency: 0.295), and 12.81 of 20 in the hepadnaviral lineage (frequency: 0.640), respectively. In a second experiment accounting for the relative branch lengths, Jane4 invariantly detected the same putative cospeciation events in all resulting solutions, three of them occurring on the nackednaviral side (frequency: 0.25) and 12 of them on the hepadnaviral side (frequency: 0.6).

(B) Virus divergence time estimates resulting from 14 independent tree inferences, each calibrated on one single node in the hepadnaviral phylogeny. Dots on each vertical dashed line represent viral node age estimates from an individual time-scaled tree. The three calibrations based on the root age of eAHBV-FRY are indicated. Node numberings as in Figures 5 and 6. Independently obtained age estimates for each node and related linear regression analyses are in the same color. The lack of a correlation (slopes of linear regressions not significantly deviating from zero) indicates that the retrieved divergence time estimates for any individual node are independent from the ages of the nodes used for tree calibration. This observation rules out a bias caused by substitution saturation which would lead to an systematic underestimation of divergence times when calibrating trees by use of young (more terminal)

nodes and an overestimation of divergence times when calibrating trees by use of ancient (more basal) nodes (van Tuinen and Torres, 2015).

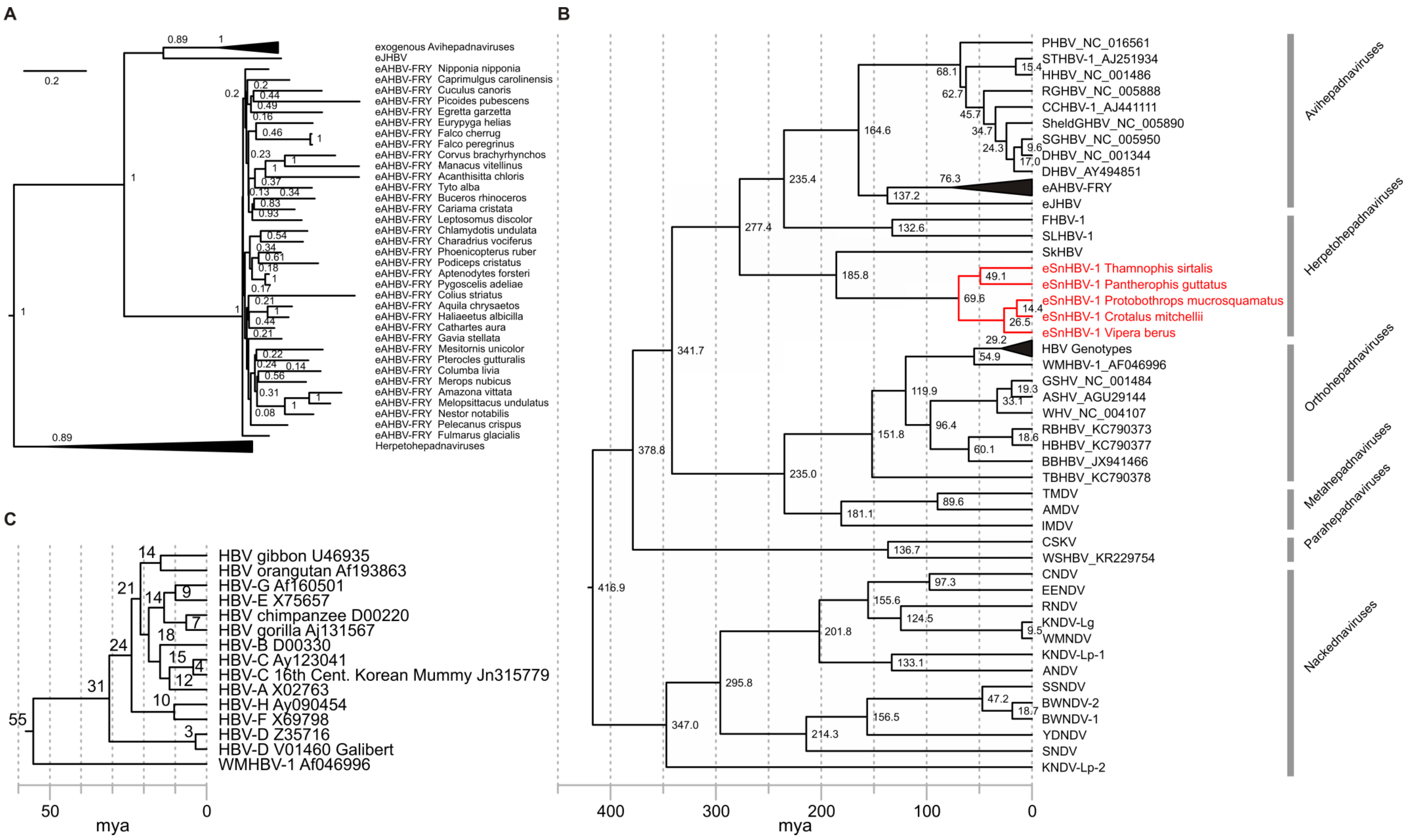
A
exogenous Avihepadnaviruses
eJHBV
eAHBV-FRY Nipponia nippon
eAHBV-FRY Caprimulgus carolinensis
eAHBV-FRY Cuculus canoris
eAHBV-FRY Picoides pubescens
eAHBV-FRY Egretta garzetta
eAHBV-FRY Eurypyga helias
eAHBV-FRY Falco cherrug
eAHBV-FRY Falco peregrinus
eAHBV-FRY Corvus brachyrhynchos
eAHBV-FRY Manacus vitellinus
eAHBV-FRY Acanthisitta chloris
eAHBV-FRY Tyto alba
eAHBV-FRY Buceros rhinoceros
eAHBV-FRY Cariama cristata
eAHBV-FRY Leptosomus discolor
eAHBV-FRY Chlamydotis undulata
eAHBV-FRY Charadrius vociferus
eAHBV-FRY Phoenicopterus ruber
eAHBV-FRY Podiceps cristatus
eAHBV-FRY Aptenodytes forsteri
eAHBV-FRY Pygoscelis adeliae
eAHBV-FRY Colius striatus
eAHBV-FRY Aquila chrysaetos
eAHBV-FRY Haliaeetus albicilla
eAHBV-FRY Cathartes aura
eAHBV-FRY Gavia stellata
eAHBV-FRY Mesitornis unicolor
eAHBV-FRY Pterocles gutturalis
eAHBV-FRY Columba livia
eAHBV-FRY Merops nubicus
eAHBV-FRY Amazona vittata
eAHBV-FRY Melopsittacus undulatus
eAHBV-FRY Nestor notabilis
eAHBV-FRY Pelecanus crispus
eAHBV-FRY Fulmarus glacialis
Herpetohepadnaviruses
0.2
B
PHBV_NC_016561
STHBV-1_AJ251934
HHBV_NC_001486
RGHBV_NC_005888
CCHBV-1_AJ441111
SheldGHBV_NC_005890
SGHBV_NC_005950
DHBV_NC_001344
DHBV_AY494851
eAHBV-FRY
eJHBV
FHBV-1
SLHBV-1
SkHBV
eSnHBV-1 Thamnophis sirtalis
eSnHBV-1 Pantherophis guttatus
eSnHBV-1 Protobothrops mucrosquamatus
eSnHBV-1 Crotalus mitchellii
eSnHBV-1 Vipera berus
HBV Genotypes
WMHBV-1_AF046996
GSHV_NC_001484
ASHV_AGU29144
WHV_NC_004107
RBHBV_KC790373
HBHBV_KC790377
BBHBV_JX941466
TBHBV_KC790378
TMDV
AMDV
IMDV
CSKV
WSHBV_KR229754
CNDV
EENDV
RNDV
KNDV-Lg
WMNDV
KNDV-Lp-1
ANDV
SSNDV
BWNDV-2
BWNDV-1
YDNDV
SNDV
KNDV-Lp-2
Avihepadnaviruses
Herpetohepadnaviruses
Orthohepadnaviruses
Metahepadnaviruses
Parahepadnaviruses
Nackednaviruses
400
300
200
100
0
mya
C
HBV gibbon U46935
HBV orangutan Af193863
HBV-G Af160501
HBV-E X75657
HBV chimpanzee D00220
HBV gorilla Aj131567
HBV-B D00330
HBV-C Ay123041
HBV-C 16th Cent. Korean Mummy Jn315779
HBV-A X02763
HBV-H Ay090454
HBV-F X69798
HBV-D Z35716
HBV-D V01460 Galibert
WMHBV-1 Af046996
50
0
mya

Figure S7, related to Figure 4. Phylogenetic relationship of endogenous avihepadnaviruses (eAHBV-*FRY*), time-calibrated tree based on endogenous snake hepatitis B virus 1 (eSnHBV-1), and time-calibrated subtree of HBV genotype isolates from humans and apes

(A) We extracted and reconstructed a set of 35 endogenous avihepadnaviral (eAHBV) P protein sequences from whole-genome sequencing data of birds (Zhang et al., 2014). These belong to an orthologous integration of an almost full-length viral genome near the *FRY* gene, and hence descended from a single genome invasion event. Since eAHBV-FRY is present in all currently sequenced genomes of Neoaves, but absent in the genomes of Galloanserae (Suh et al., 2013), the integration must have occurred in the ancestry of Neoaves after divergence from galloanserine birds, i.e. between 89 and 69 mya (Jarvis et al., 2014). Depicted is a subtree of the uncalibrated Bayesian phylogenetic tree in Figure S4A. The eAHBV-FRY cluster is shown expanded and extant exogenous avihepadnaviruses and herpetohepadnaviruses are included as outgroups. The explosive diversification of the eAHBV-FRY isolates closely resembles the rapid adaptive radiation of Neoaves which started 69—67 mya (Jarvis et al., 2014; Prum et al., 2015; Claramunt and Cracraft, 2015). Consequently, we assume concomitant diversification and used this age for dating the root of eAHBV-*FRY* to time-calibrate the viral phylogeny (see Figures 6 and S6B). Numbers at branching points show posterior probability support values. Scale bar: 0.2 amino acid substitutions per site.

(B) Time-calibrated Bayesian tree based on P sequences of eSnHBV-1 from five member species of the snake superfamily Colubroidea (marked in red). Numbers indicate node age estimates in mya. eSnHBV-1 was first detected in the genome of the speckled rattlesnake (*Crotalus mitchellii*) (Gilbert et al., 2014; Suh et al., 2014). We assembled and reconstructed additional, almost full-length eSnHBV-1 P sequences from the European adder (*Vipera berus*),

the brown spotted pit viper (*Protobothrops mucrosquamatus*), the corn snake (*Pantherophis guttatus*) and the common garter snake (*Thamnophis sirtalis*). 417 aa positions of the P protein alignment (Supplemental Data File S2) were utilized for reconstructing time-calibrated phylogenies. We computed a consensus tree from two independent Bayesian phylogenetic inferences (JTT+G4 model, relaxed molecular clock with log-normal distribution, Yules speciation prior), in which we dated the root of the eSnHBV-1 P protein sequences using published age estimates for the most recent common ancestor of Colubroidea of 61 mya (Pyron and Burbrink, 2012; Zheng and Wiens, 2016) or 77 mya (Castoe et al., 2009; Kyriazi et al., 2013), respectively. The retrieved node age estimates of this consensus tree match very well with those obtained in the calibrations based eAHBV-FRY (Figure 4). For example, the separation of nackedna- and hepadnaviruses was estimated to have occurred 417 mya and the root age of eAHBV-FRY was determined as 76 mya (compare to Figures 4 and 6).

(C) Time-calibrated subtree of the main tree in Figure 4 with the HBV genotype isolates from humans and apes shown expanded. Numbers indicate node age estimates in mya. Woolly monkey hepatitis B virus (WMHBV) from the New world monkey *Lagothrix lagotricha* included as outgroup.

Table S1, related to Figure 1. Synopsis of novel HBV-related viruses described in this study

Virus	Host; common name (scientific name)	Seq. type[a]	Source[b]	Tissue/organ	Remarks
**Nackednaviruses RNDV-type**					
RNDV	Tiger rockfish (*Sebastes nigrocinctus*)	WGS	AUPR01188533	Fin	Further hits in unpublished SRA
ACNDV	African cichlid (*Ophthalmotilapia ventralis*)	TS	gb|JL559376	Pooled organs	Further hits: gb|JL576735.1; SRX078329
ANDV	Astatotilapia (*Astatotilapia sp.*)	WGS	ERX240954	Unknown	Bioproject with 433 WGS experiments of individual fish
EENDV	European eel (*Anguilla anguilla*)	TS	SRX700630	Olfactory epithelium	
KNDV-Lg	Bluefin killifish (*Lucania goodei*)	TS	SRX340220	Pooled organs	Gill, eye, fin, testis, ovary, brain
KNDV-Lp-1	Rainwater killifish (*Lucania parva*)	TS	SRX340836	Pooled organs	PolyA-tail identified; gill, eye, fin, testis, ovary, brain
WMNDV	Western mosquitofish (*Gambusia affinis*)	TS	SRX376926	Ovary	
**Nackednaviruses SSNDV-type**					
SSNDV	Sockeye salmon (*Oncorhynchus nerka*)	TS	SRX265393	Pooled organs	PolyA-tail identified; heart, liver gonad, muscle, olfact. bulb
BWNDV-1	Baby whale (*Brienomyrus brachyistius*)	TS	SRX553136	Muscle	
BWNDV-2	Baby whale (*Brienomyrus brachyistius*)	TS	SRX573075	Electric organ	PolyA-tail identified
SNDV	Three-spined stickleback (*G. aculeatus*)	WGS	SRX1037831	Unknown	
YDNDV	Yellow drum (*Nibea albiflora*)	TS	SRX367575	Unknown	
**Nackednaviruses KNDV-Lp-2-type**					
KNDV-Lp-2	Rainwater killifish (*Lucania parva*)	TS	SRX340853	Pooled organs	Gill, eye, fin, testis, ovary, brain
**Parahepadnaviruses**					
CSKV	Coho salmon (*Oncorhynchus kisutch*)	TS	SRX1037831	Kidney	Not in liver and spleen => Coho salmon kidney virus
**Metahepadnaviruses**					
AMDV	Astatotilapia (*Astatotilapia sp.*)	WGS	ERX674915	Unknown	Bioproject with 433 WGS experiments of individual fish
IMDV	Crocodile icefish (*Chionodraco hamatus*)	TS	SRX145766	Muscle	
TMDV	Mexican tetra (*Astyanax mexicanus*)	TS	SRX229523	Eyes-surface	
**Herpetohepadnaviruses**					
TFHBV	Tibetan frog (*Nanorana parkeri*)	WGS	PRJNA243398	Muscle	High sequence coverage; probably high titer viremia
SkHBV	Skink (*Saproscincus basiliscus*)	TS	SRX213382	Unknown	
SLHBV-1	Spiny lizard (*Sceloporus adleri*)	WGS	SRX542351	Liver	pooled with heart, muscle
**Avihepadnaviruses**					
eJHBV	Dark-eyed junco (*Junco hyemalis*)	TS	PRJNA158927	Pooled organs	14 organs including liver

[a]Sequencing type: WGS: whole-genome shotgun sequencing; TS: transcriptome shotgun sequencing. [b]Source annotations: ERX and SRX are SRA experiments at NCBI; PRJNA are Bioprojects at NCBI; all others are genbank accession numbers.
