# Supplementary material for: Deciphering the Origin and Evolution of Hepatitis B Viruses by Means of a Family of Non-enveloped Fish Viruses
Source: Cell Host Microbe. 2017 Sep 13;22(3):387–399.e6. doi: 10.1016/j.chom.2017.07.019 (PMC5604429; doi:10.1016/j.chom.2017.07.019)
Supplement: Data S3. P Protein Alignment in pdf Format, Related to Figures 1 and 4–7 — Conserved functionally important motifs in TP, RT, and RH are boxed. Gray bars indicate the regions used for the phylogenetic tree inferences. [file mmc6.pdf]

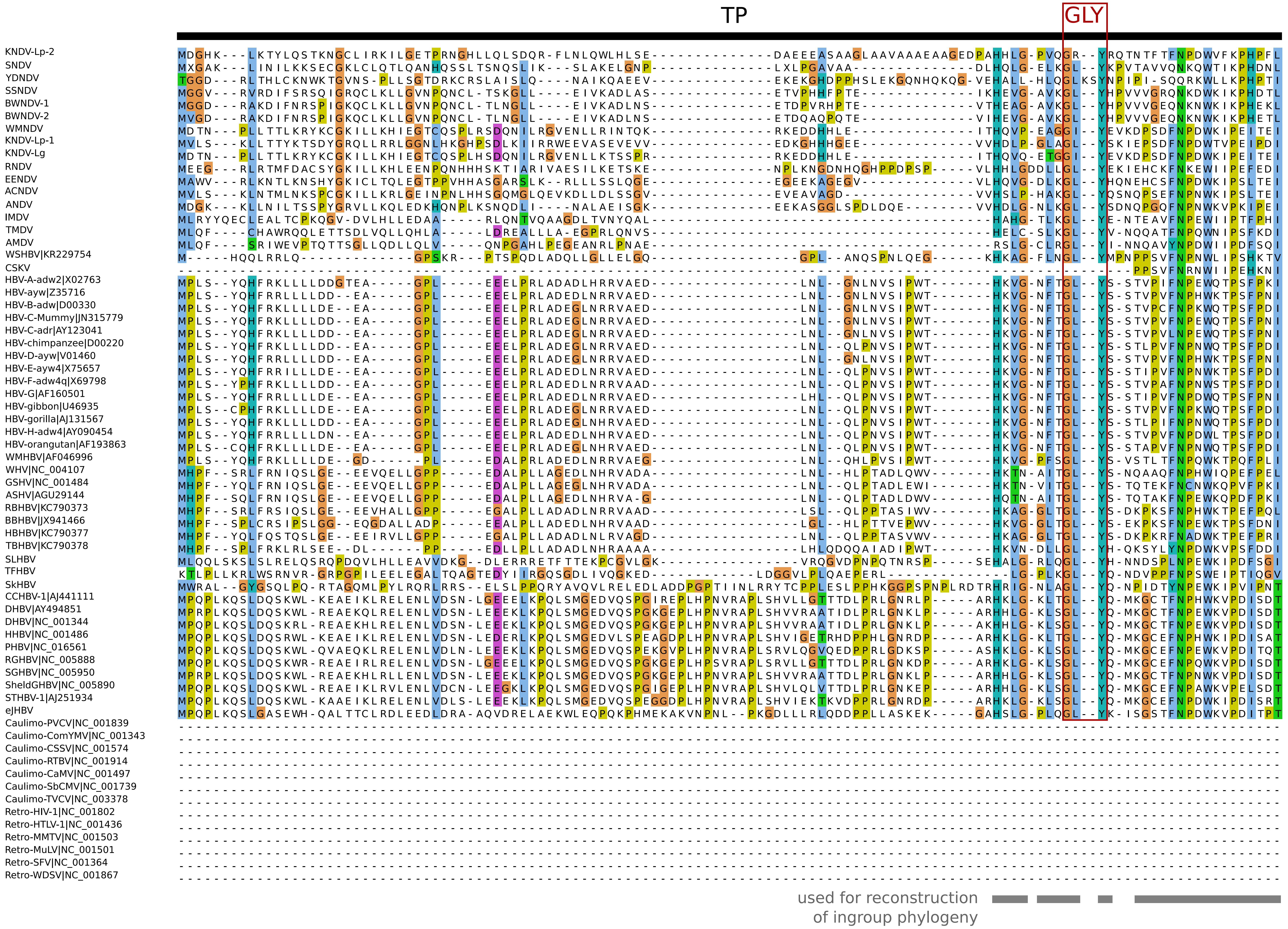

TP
GLY
KNDV-Lp-2
SNDV
YDNDV
SSNDV
BWNDV-1
BWNDV-2
WMNDV
KNDV-Lp-1
KNDV-Lg
RNDV
EENDV
ACNDV
ANDV
IMDV
TMDV
AMDV
WSHBV|KR229754
CSKV
HBV-A-adw2|X02763
HBV-ayw|Z35716
HBV-B-adw|D00330
HBV-C-Mummy|JN315779
HBV-C-adr|AY123041
HBV-chimpanzee|D00220
HBV-D-ayw|V01460
HBV-E-ayw4|X75657
HBV-F-adw4q|X69798
HBV-G|AF160501
HBV-gibbon|U46935
HBV-gorilla|AJ131567
HBV-H-adw4|AY090454
HBV-orangutan|AF193863
WMHBV|AF046996
WHV|NC_004107
GSHV|NC_001484
ASHV|AGU29144
RBHBV|KC790373
BBHBV|JX941466
HBHBV|KC790377
TBHBV|KC790378
SLHBV
TFHBV
SkHBV
CCHBV-1|AJ441111
DHBV|AY494851
DHBV|NC_001344
HHBV|NC_001486
PHBV|NC_016561
RGHBV|NC_005888
SGHBV|NC_005950
SheldGHBV|NC_005890
STHBV-1|AJ251934
eJHBV
Caulimo-PVCV|NC_001839
Caulimo-ComYMV|NC_001343
Caulimo-CSSV|NC_001574
Caulimo-RTBV|NC_001914
Caulimo-CaMV|NC_001497
Caulimo-SbCMV|NC_001739
Caulimo-TVCV|NC_003378
Retro-HIV-1|NC_001802
Retro-HTLV-1|NC_001436
Retro-MMTV|NC_001503
Retro-MuLV|NC_001501
Retro-SFV|NC_001364
Retro-WDSV|NC_001867
used for reconstruction of ingroup phylogeny

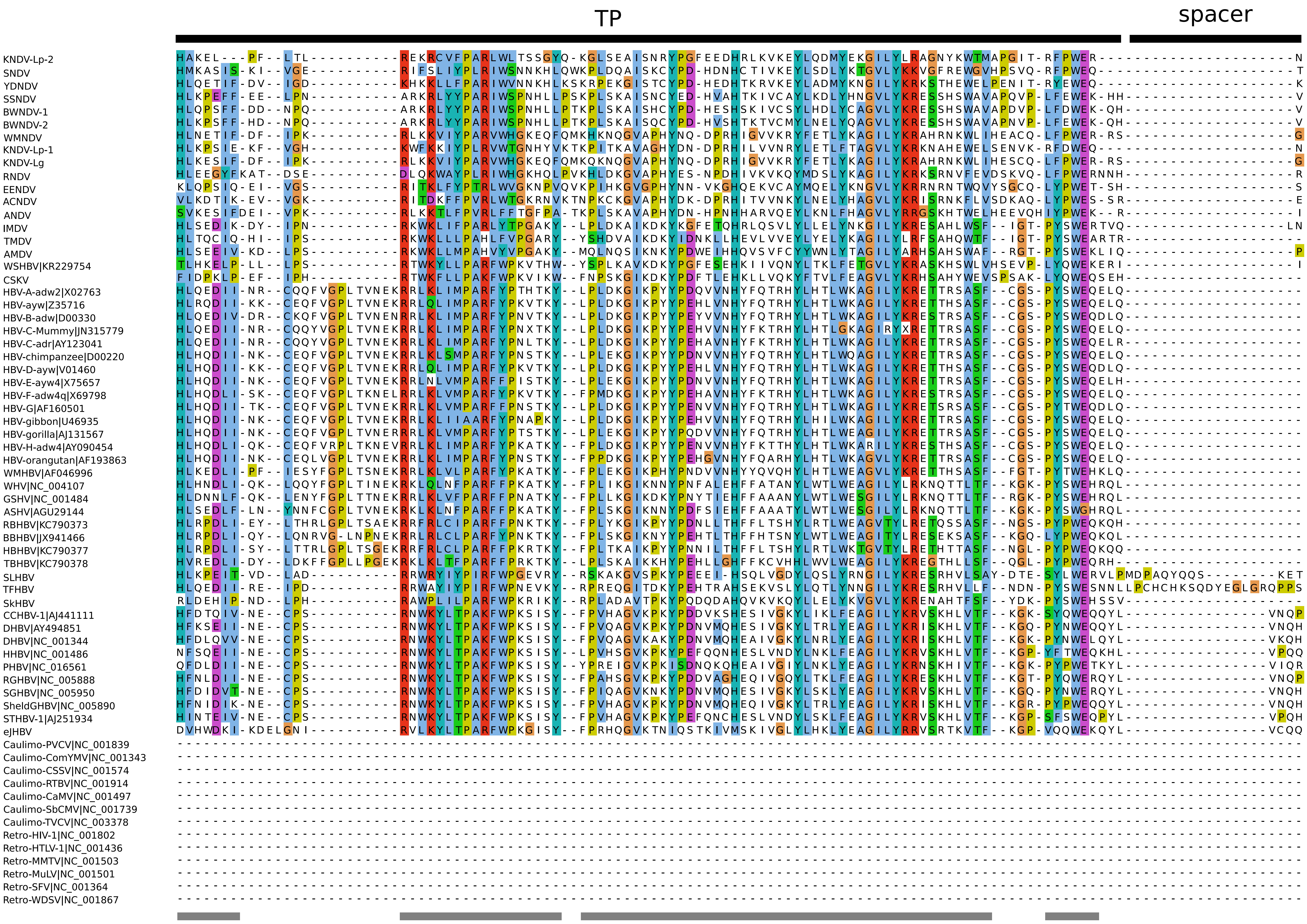
TP
spacer
KNDV-Lp-2
SNDV
YDNDV
SSNDV
BWNDV-1
BWNDV-2
WMNDV
KNDV-Lp-1
KNDV-Lg
RNDV
EENDV
ACNDV
ANDV
IMDV
TMDV
AMDV
WSHBV|KR229754
CSKV
HBV-A-adw2|X02763
HBV-ayw|Z35716
HBV-B-adw|D00330
HBV-C-Mummy|JN315779
HBV-C-adr|AY123041
HBV-chimpanzee|D00220
HBV-D-ayw|V01460
HBV-E-ayw4|X75657
HBV-F-adw4q|X69798
HBV-G|AF160501
HBV-gibbon|U46935
HBV-gorilla|AJ131567
HBV-H-adw4|AY090454
HBV-orangutan|AF193863
WMHBV|AF046996
WHV|NC_004107
GSHV|NC_001484
ASHV|AGU29144
RBHBV|KC790373
BBHBV|JX941466
HBHBV|KC790377
TBHBV|KC790378
SLHBV
TFHBV
SkHBV
CCHBV-1|AJ441111
DHBV|AY494851
DHBV|NC_001344
HHBV|NC_001486
PHBV|NC_016561
RGHBV|NC_005888
SGHBV|NC_005950
SheldGHBV|NC_005890
STHBV-1|AJ251934
eJHBV
Caulimo-PVCV|NC_001839
Caulimo-ComYMV|NC_001343
Caulimo-CSSV|NC_001574
Caulimo-RTBV|NC_001914
Caulimo-CaMV|NC_001497
Caulimo-SbCMV|NC_001739
Caulimo-TVCV|NC_003378
Retro-HIV-1|NC_001802
Retro-HTLV-1|NC_001436
Retro-MMTV|NC_001503
Retro-MuLV|NC_001501
Retro-SFV|NC_001364
Retro-WDSV|NC_001867

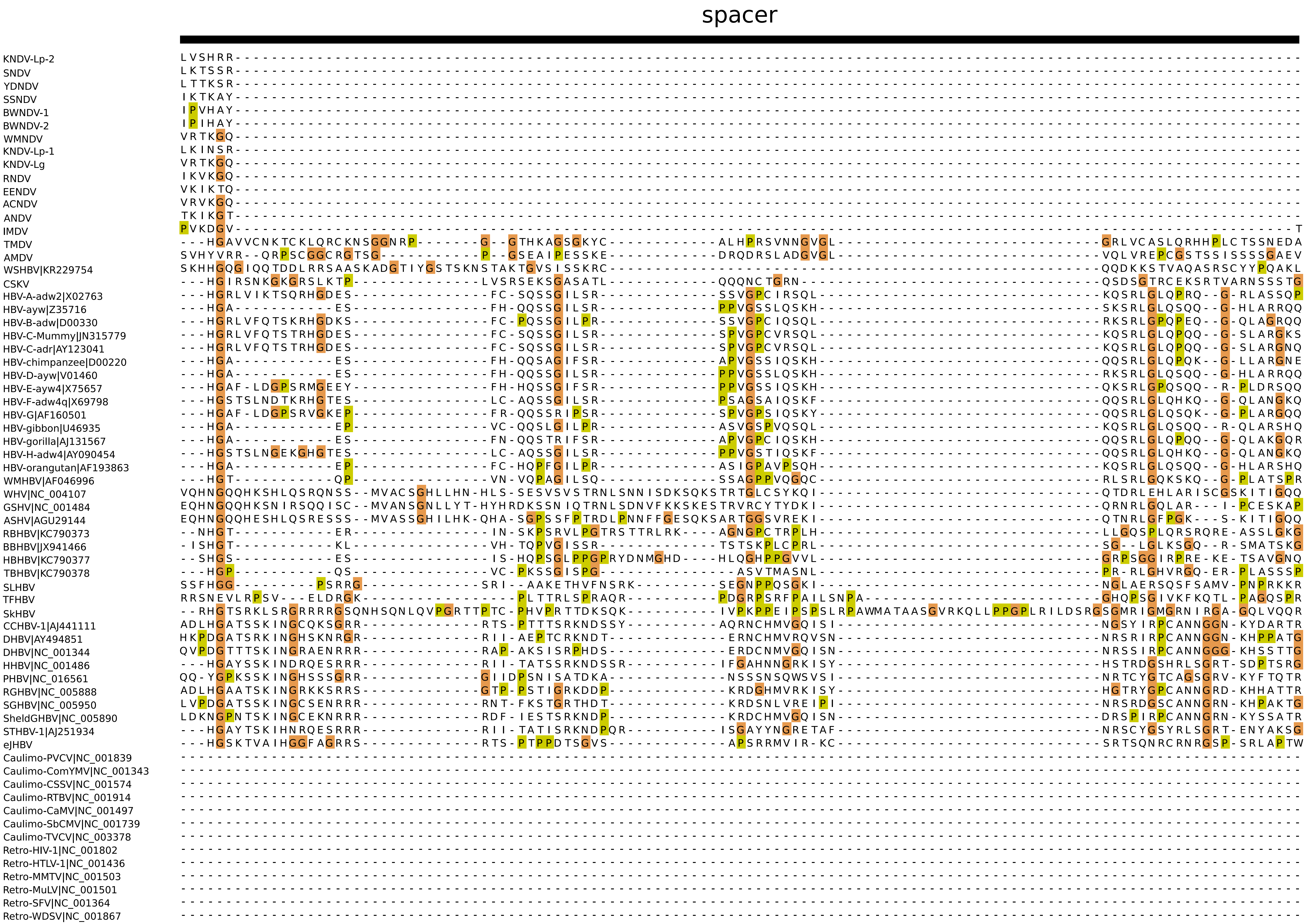
spacer
KNDV-Lp-2
SNDV
YDNDV
SSNDV
BWNDV-1
BWNDV-2
WMNDV
KNDV-Lp-1
KNDV-Lg
RNDV
EENDV
ACNDV
ANDV
IMDV
TMDV
AMDV
WSHBV|KR229754
CSKV
HBV-A-adw2|X02763
HBV-ayw|Z35716
HBV-B-adw|D00330
HBV-C-Mummy|JN315779
HBV-C-adr|AY123041
HBV-chimpanzee|D00220
HBV-D-ayw|V01460
HBV-E-ayw4|X75657
HBV-F-adw4q|X69798
HBV-G|AF160501
HBV-gibbon|U46935
HBV-gorilla|AJ131567
HBV-H-adw4|AY090454
HBV-orangutan|AF193863
WMHBV|AF046996
WHV|NC_004107
GSHV|NC_001484
ASHV|AGU29144
RBHBV|KC790373
BBHBV|JX941466
HBHBV|KC790377
TBHBV|KC790378
SLHBV
TFHBV
SkHBV
CCHBV-1|AJ441111
DHBV|AY494851
DHBV|NC_001344
HHBV|NC_001486
PHBV|NC_016561
RGHBV|NC_005888
SGHBV|NC_005950
SheldGHBV|NC_005890
STHBV-1|AJ251934
eJHBV
Caulimo-PVCV|NC_001839
Caulimo-ComYMV|NC_001343
Caulimo-CSSV|NC_001574
Caulimo-RTBV|NC_001914
Caulimo-CaMV|NC_001497
Caulimo-SbCMV|NC_001739
Caulimo-TVCV|NC_003378
Retro-HIV-1|NC_001802
Retro-HTLV-1|NC_001436
Retro-MMTV|NC_001503
Retro-MuLV|NC_001501
Retro-SFV|NC_001364
Retro-WDSV|NC_001867

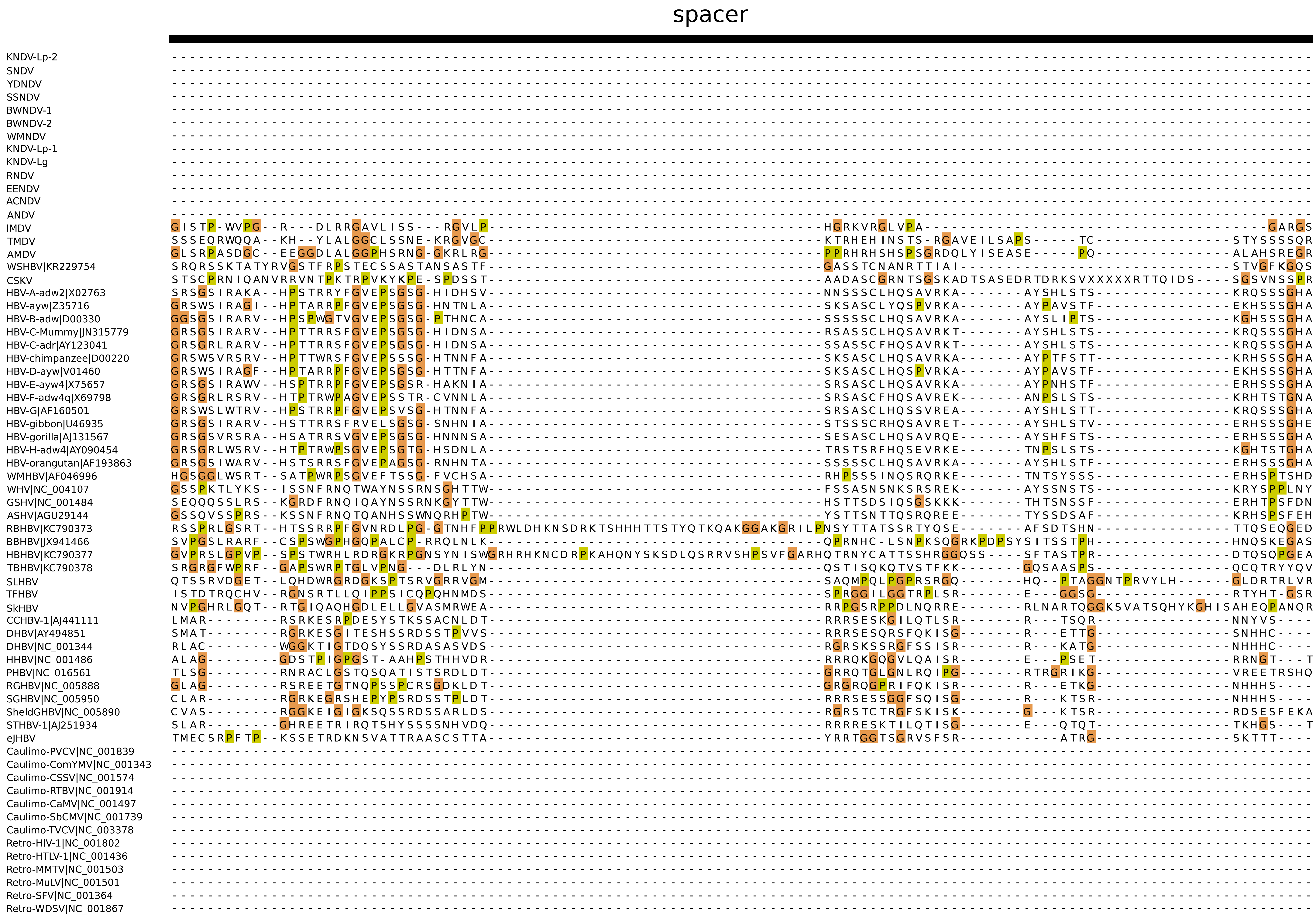
spacer
KNDV-Lp-2
SNDV
YDNDV
SSNDV
BWNDV-1
BWNDV-2
WMNDV
KNDV-Lp-1
KNDV-Lg
RNDV
EENDV
ACNDV
ANDV
IMDV
TMDV
AMDV
WSHBV|KR229754
CSKV
HBV-A-adw2|X02763
HBV-ayw|Z35716
HBV-B-adw|D00330
HBV-C-Mummy|JN315779
HBV-C-adr|AY123041
HBV-chimpanzee|D00220
HBV-D-ayw|V01460
HBV-E-ayw4|X75657
HBV-F-adw4q|X69798
HBV-G|AF160501
HBV-gibbon|U46935
HBV-gorilla|AJ131567
HBV-H-adw4|AY090454
HBV-orangutan|AF193863
WMHBV|AF046996
WHV|NC_004107
GSHV|NC_001484
ASHV|AGU29144
RBHBV|KC790373
BBHBV|JX941466
HBHBV|KC790377
TBHBV|KC790378
SLHBV
TFHBV
SkHBV
CCHBV-1|AJ441111
DHBV|AY494851
DHBV|NC_001344
HHBV|NC_001486
PHBV|NC_016561
RGHBV|NC_005888
SGHBV|NC_005950
SheldGHBV|NC_005890
STHBV-1|AJ251934
eJHBV
Caulimo-PVCV|NC_001839
Caulimo-ComYMV|NC_001343
Caulimo-CSSV|NC_001574
Caulimo-RTBV|NC_001914
Caulimo-CaMV|NC_001497
Caulimo-SbCMV|NC_001739
Caulimo-TVCV|NC_003378
Retro-HIV-1|NC_001802
Retro-HTLV-1|NC_001436
Retro-MMTV|NC_001503
Retro-MuLV|NC_001501
Retro-SFV|NC_001364
Retro-WDSV|NC_001867

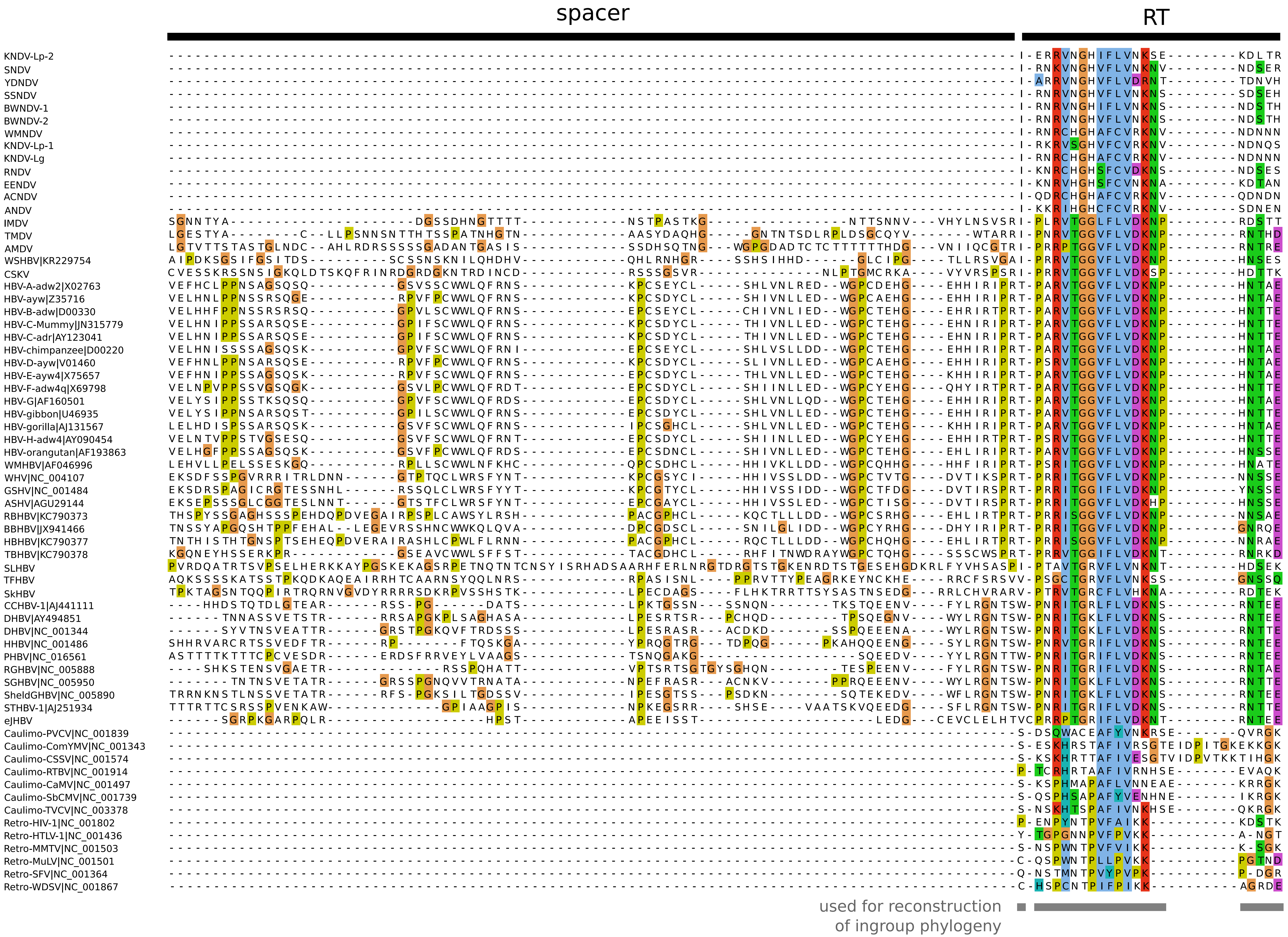

spacer
RT
KNDV-Lp-2
SNDV
YDNDV
SSNDV
BWNDV-1
BWNDV-2
WMNDV
KNDV-Lp-1
KNDV-Lg
RNDV
EENDV
ACNDV
ANDV
IMDV
TMDV
AMDV
WSHBV|KR229754
CSKV
HBV-A-adw2|X02763
HBV-ayw|Z35716
HBV-B-adw|D00330
HBV-C-Mummy|JN315779
HBV-C-adr|AY123041
HBV-chimpanzee|D00220
HBV-D-ayw|V01460
HBV-E-ayw4|X75657
HBV-F-adw4q|X69798
HBV-G|AF160501
HBV-gibbon|U46935
HBV-gorilla|AJ131567
HBV-H-adw4|AY090454
HBV-orangutan|AF193863
WMHBV|AF046996
WHV|NC_004107
GSHV|NC_001484
ASHV|AGU29144
RBHBV|KC790373
BBHBV|JX941466
HBHBV|KC790377
TBHBV|KC790378
SLHBV
TFHBV
SkHBV
CCHBV-1|AJ441111
DHBV|AY494851
DHBV|NC_001344
HHBV|NC_001486
PHBV|NC_016561
RGHBV|NC_005888
SGHBV|NC_005950
SheldGHBV|NC_005890
STHBV-1|AJ251934
eJHBV
Caulimo-PVCV|NC_001839
Caulimo-ComYMV|NC_001343
Caulimo-CSSV|NC_001574
Caulimo-RTBV|NC_001914
Caulimo-CaMV|NC_001497
Caulimo-SbCMV|NC_001739
Caulimo-TVCV|NC_003378
Retro-HIV-1|NC_001802
Retro-HTLV-1|NC_001436
Retro-MMTV|NC_001503
Retro-MuLV|NC_001501
Retro-SFV|NC_001364
Retro-WDSV|NC_001867
used for reconstruction
of ingroup phylogeny

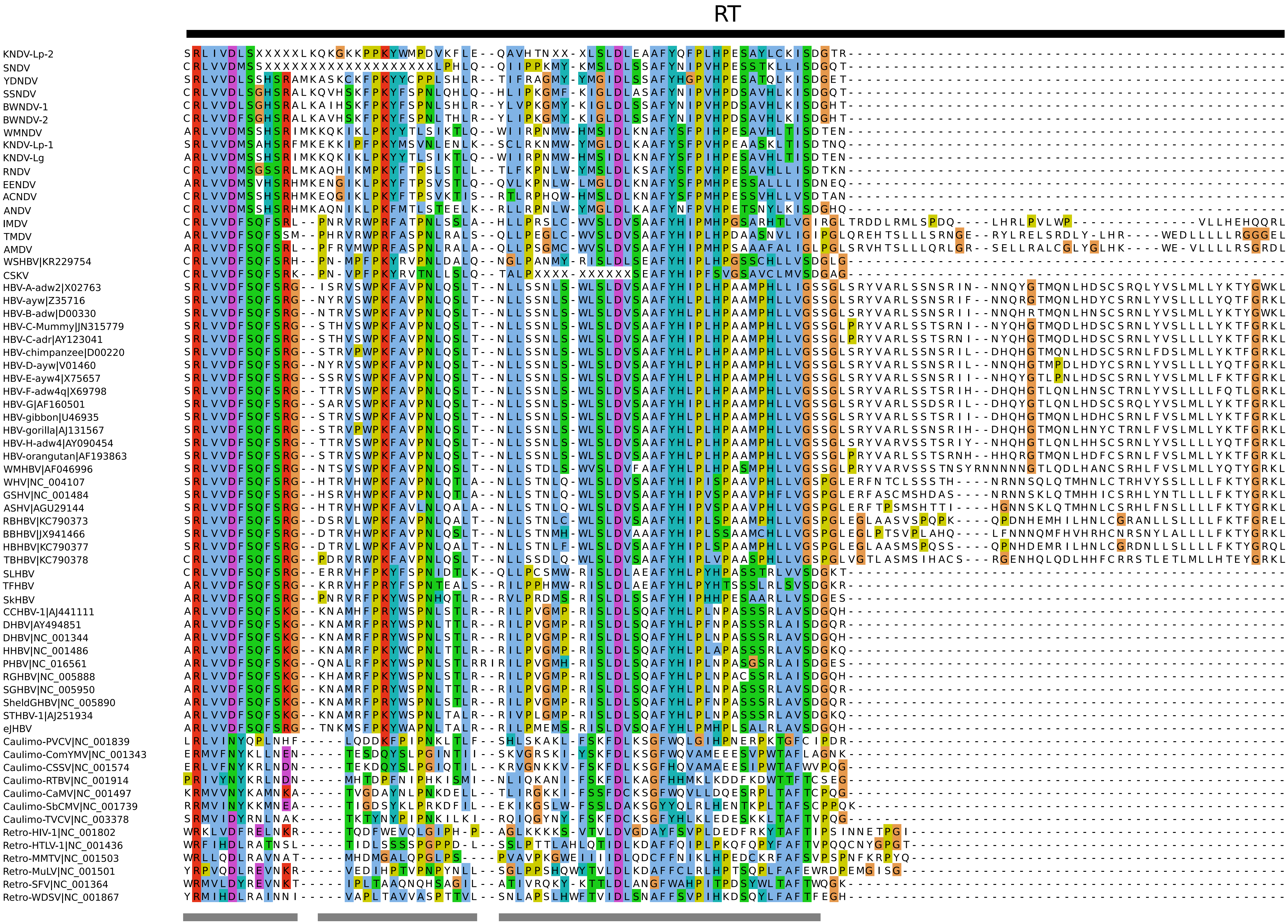
RT
KNDV-Lp-2
SNDV
YDNDV
SSNDV
BWNDV-1
BWNDV-2
WMNDV
KNDV-Lp-1
KNDV-Lg
RNDV
EENDV
ACNDV
ANDV
IMDV
TMDV
AMDV
WSHBV|KR229754
CSKV
HBV-A-adw2|X02763
HBV-ayw|Z35716
HBV-B-adw|D00330
HBV-C-Mummy|JN315779
HBV-C-adr|AY123041
HBV-chimpanzee|D00220
HBV-D-ayw|V01460
HBV-E-ayw4|X75657
HBV-F-adw4q|X69798
HBV-G|AF160501
HBV-gibbon|U46935
HBV-gorilla|AJ131567
HBV-H-adw4|AY090454
HBV-orangutan|AF193863
WMHBV|AF046996
WHV|NC_004107
GSHV|NC_001484
ASHV|AGU29144
RBHBV|KC790373
BBHBV|JX941466
HBHBV|KC790377
TBHBV|KC790378
SLHBV
TFHBV
SkHBV
CCHBV-1|AJ441111
DHBV|AY494851
DHBV|NC_001344
HHBV|NC_001486
PHBV|NC_016561
RGHBV|NC_005888
SGHBV|NC_005950
SheldGHBV|NC_005890
STHBV-1|AJ251934
eJHBV
Caulimo-PVCV|NC_001839
Caulimo-ComYMV|NC_001343
Caulimo-CSSV|NC_001574
Caulimo-RTBV|NC_001914
Caulimo-CaMV|NC_001497
Caulimo-SbCMV|NC_001739
Caulimo-TVCV|NC_003378
Retro-HIV-1|NC_001802
Retro-HTLV-1|NC_001436
Retro-MMTV|NC_001503
Retro-MuLV|NC_001501
Retro-SFV|NC_001364
Retro-WDSV|NC_001867

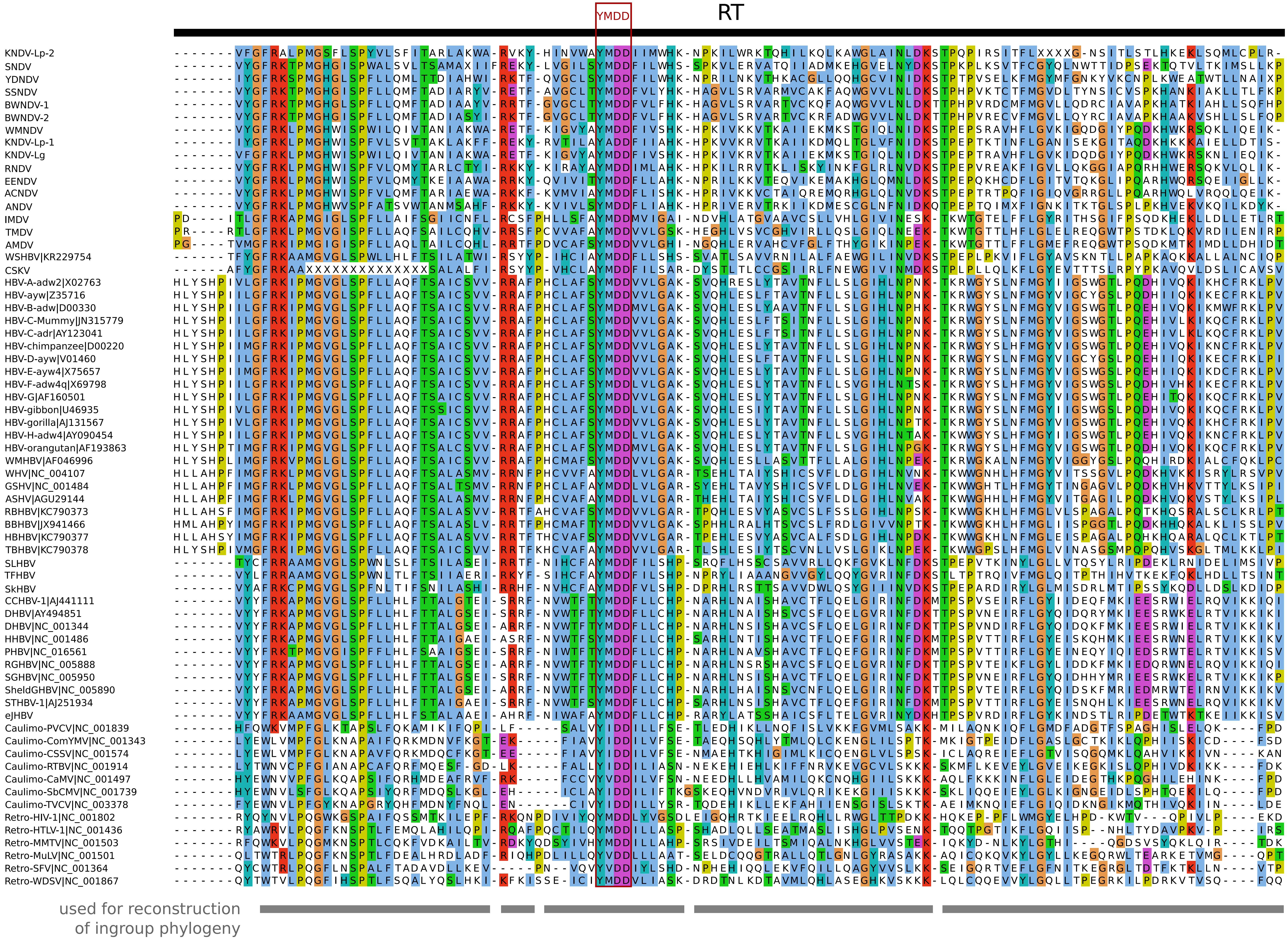

RT
YMDD
KNDV-Lp-2
SNDV
YDNDV
SSNDV
BWNDV-1
BWNDV-2
WMNDV
KNDV-Lp-1
KNDV-Lg
RNDV
EENDV
ACNDV
ANDV
IMDV
TMDV
AMDV
WSHBV|KR229754
CSKV
HBV-A-adw2|X02763
HBV-ayw|Z35716
HBV-B-adw|D00330
HBV-C-Mummy|JN315779
HBV-C-adr|AY123041
HBV-chimpanzee|D00220
HBV-D-ayw|V01460
HBV-E-ayw4|X75657
HBV-F-adw4q|X69798
HBV-G|AF160501
HBV-gibbon|U46935
HBV-gorilla|AJ131567
HBV-H-adw4|AY090454
HBV-orangutan|AF193863
WMHBV|AF046996
WHV|NC_004107
GSHV|NC_001484
ASHV|AGU29144
RBHBV|KC790373
BBHBV|JX941466
HBHBV|KC790377
TBHBV|KC790378
SLHBV
TFHBV
SkHBV
CCHBV-1|AJ441111
DHBV|AY494851
DHBV|NC_001344
HHBV|NC_001486
PHBV|NC_016561
RGHBV|NC_005888
SGHBV|NC_005950
SheldGHBV|NC_005890
STHBV-1|AJ251934
eJHBV
Caulimo-PVCV|NC_001839
Caulimo-ComYMV|NC_001343
Caulimo-CSSV|NC_001574
Caulimo-RTBV|NC_001914
Caulimo-CaMV|NC_001497
Caulimo-SbCMV|NC_001739
Caulimo-TVCV|NC_003378
Retro-HIV-1|NC_001802
Retro-HTLV-1|NC_001436
Retro-MMTV|NC_001503
Retro-MuLV|NC_001501
Retro-SFV|NC_001364
Retro-WDSV|NC_001867
used for reconstruction
of ingroup phylogeny

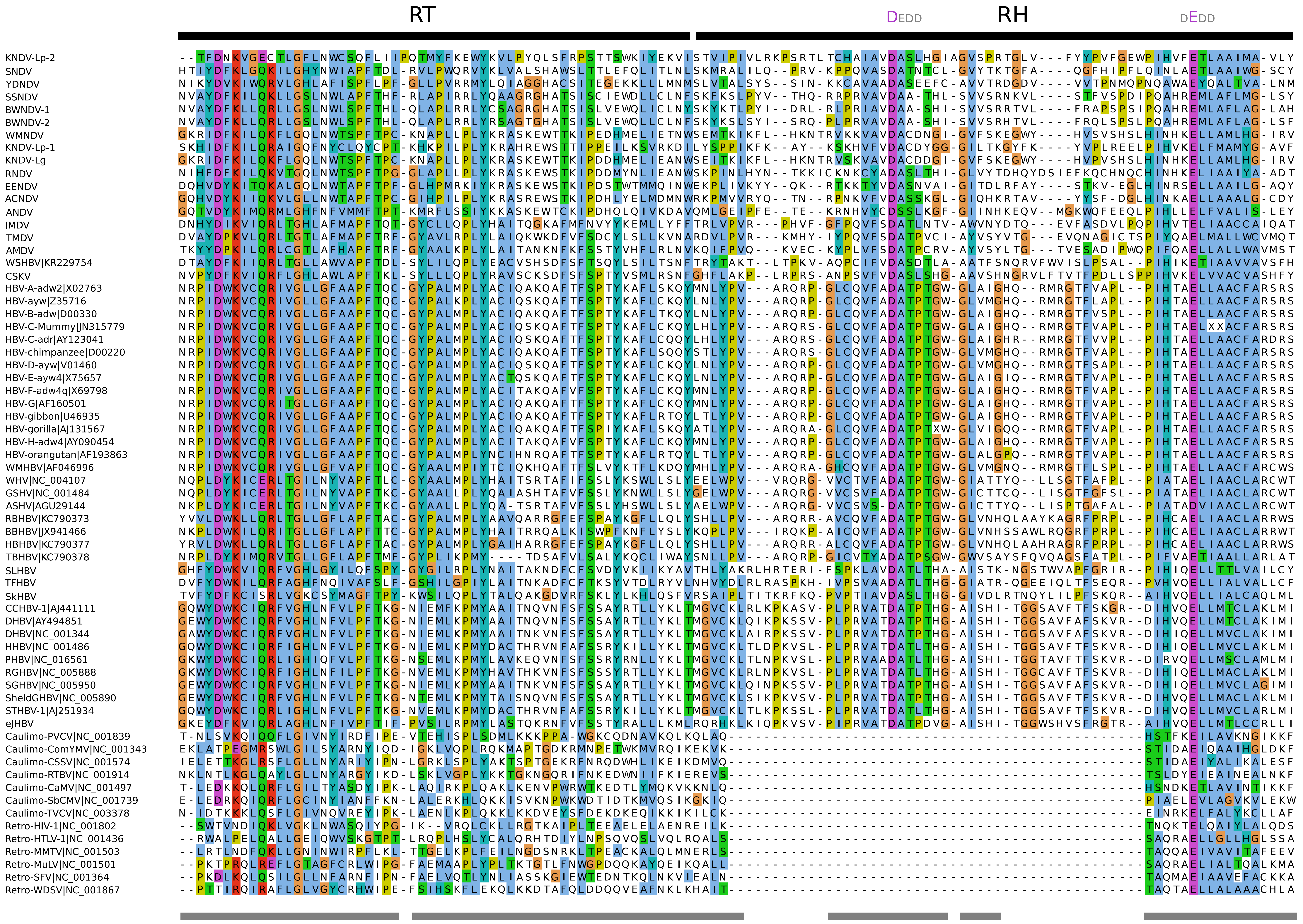
RT
DEDD
RH
dEDD
KNDV-Lp-2
SNDV
YDNDV
SSNDV
BWNDV-1
BWNDV-2
WMNDV
KNDV-Lp-1
KNDV-Lg
RNDV
EENDV
ACNDV
ANDV
IMDV
TMDV
AMDV
WSHBV|KR229754
CSKV
HBV-A-adw2|X02763
HBV-ayw|Z35716
HBV-B-adw|D00330
HBV-C-Mummy|JN315779
HBV-C-adr|AY123041
HBV-chimpanzee|D00220
HBV-D-ayw|V01460
HBV-E-ayw4|X75657
HBV-F-adw4q|X69798
HBV-G|AF160501
HBV-gibbon|U46935
HBV-gorilla|AJ131567
HBV-H-adw4|AY090454
HBV-orangutan|AF193863
WMHBV|AF046996
WHV|NC_004107
GSHV|NC_001484
ASHV|AGU29144
RBHBV|KC790373
BBHBV|JX941466
HBHBV|KC790377
TBHBV|KC790378
SLHBV
TFHBV
SkHBV
CCHBV-1|AJ441111
DHBV|AY494851
DHBV|NC_001344
HHBV|NC_001486
PHBV|NC_016561
RGHBV|NC_005888
SGHBV|NC_005950
SheldGHBV|NC_005890
STHBV-1|AJ251934
eJHBV
Caulimo-PVCV|NC_001839
Caulimo-ComYMV|NC_001343
Caulimo-CSSV|NC_001574
Caulimo-RTBV|NC_001914
Caulimo-CaMV|NC_001497
Caulimo-SbCMV|NC_001739
Caulimo-TVCV|NC_003378
Retro-HIV-1|NC_001802
Retro-HTLV-1|NC_001436
Retro-MMTV|NC_001503
Retro-MuLV|NC_001501
Retro-SFV|NC_001364
Retro-WDSV|NC_001867

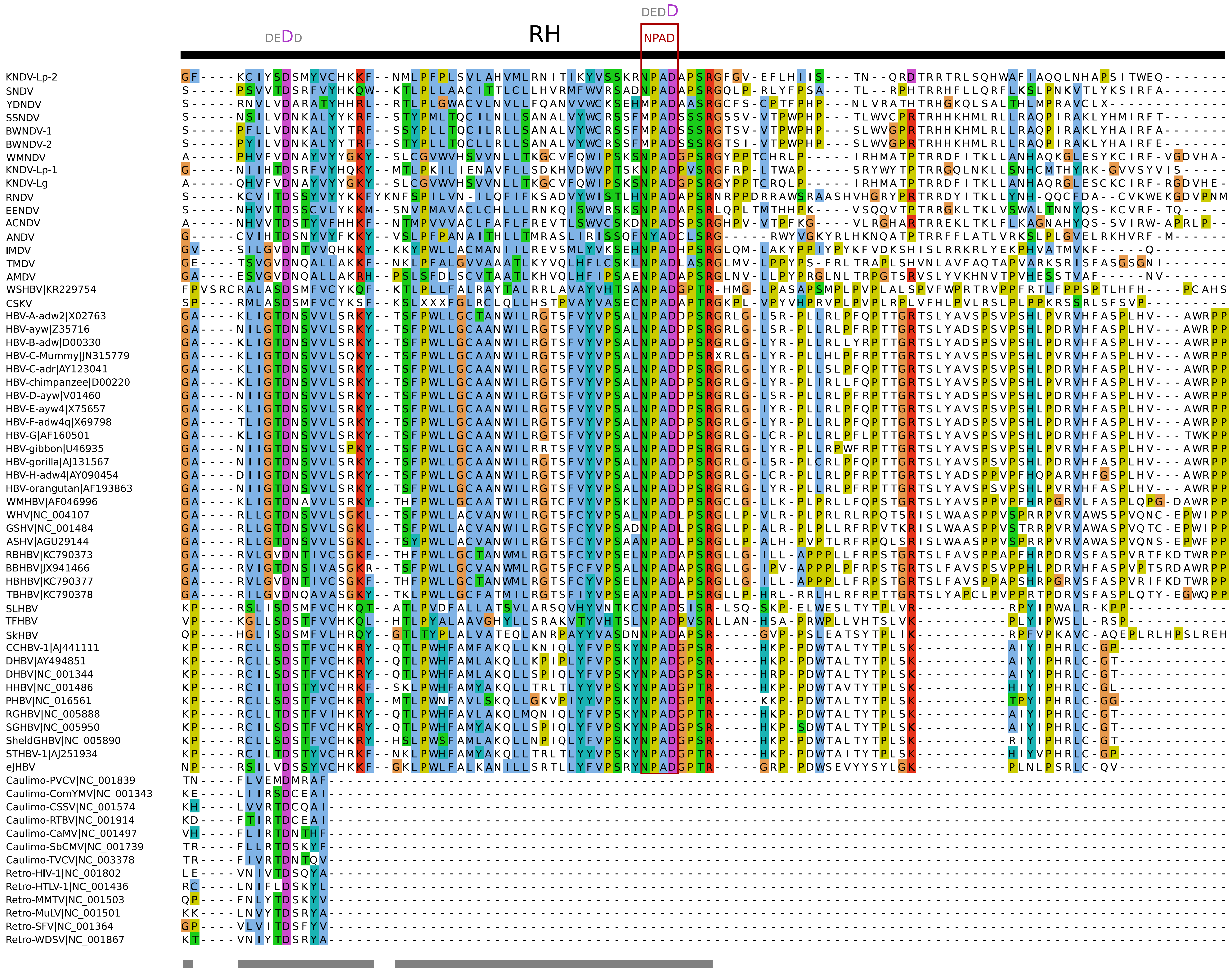
DEDD
RH
DEDD
NPAD
KNDV-Lp-2
SNDV
YDNDV
SSNDV
BWNDV-1
BWNDV-2
WMNDV
KNDV-Lp-1
KNDV-Lg
RNDV
EENDV
ACNDV
ANDV
IMDV
TMDV
AMDV
WSHBV|KR229754
CSKV
HBV-A-adw2|X02763
HBV-ayw|Z35716
HBV-B-adw|D00330
HBV-C-Mummy|JN315779
HBV-C-adr|AY123041
HBV-chimpanzee|D00220
HBV-D-ayw|V01460
HBV-E-ayw4|X75657
HBV-F-adw4q|X69798
HBV-G|AF160501
HBV-gibbon|U46935
HBV-gorilla|AJ131567
HBV-H-adw4|AY090454
HBV-orangutan|AF193863
WMHBV|AF046996
WHV|NC_004107
GSHV|NC_001484
ASHV|AGU29144
RBHBV|KC790373
BBHBV|JX941466
HBHBV|KC790377
TBHBV|KC790378
SLHBV
TFHBV
SkHBV
CCHBV-1|AJ441111
DHBV|AY494851
DHBV|NC_001344
HHBV|NC_001486
PHBV|NC_016561
RGHBV|NC_005888
SGHBV|NC_005950
SheldGHBV|NC_005890
STHBV-1|AJ251934
eJHBV
Caulimo-PVCV|NC_001839
Caulimo-ComYMV|NC_001343
Caulimo-CSSV|NC_001574
Caulimo-RTBV|NC_001914
Caulimo-CaMV|NC_001497
Caulimo-SbCMV|NC_001739
Caulimo-TVCV|NC_003378
Retro-HIV-1|NC_001802
Retro-HTLV-1|NC_001436
Retro-MMTV|NC_001503
Retro-MuLV|NC_001501
Retro-SFV|NC_001364
Retro-WDSV|NC_001867
