# Supplementary material for: Deciphering the Origin and Evolution of Hepatitis B Viruses by Means of a Family of Non-enveloped Fish Viruses
Source: Cell Host Microbe. 2017 Sep 13;22(3):387–399.e6. doi: 10.1016/j.chom.2017.07.019 (PMC5604429; doi:10.1016/j.chom.2017.07.019)
Supplement: Data S4. Core Protein Alignment in pdf Format, Related to Figure 3 [file mmc7.pdf]

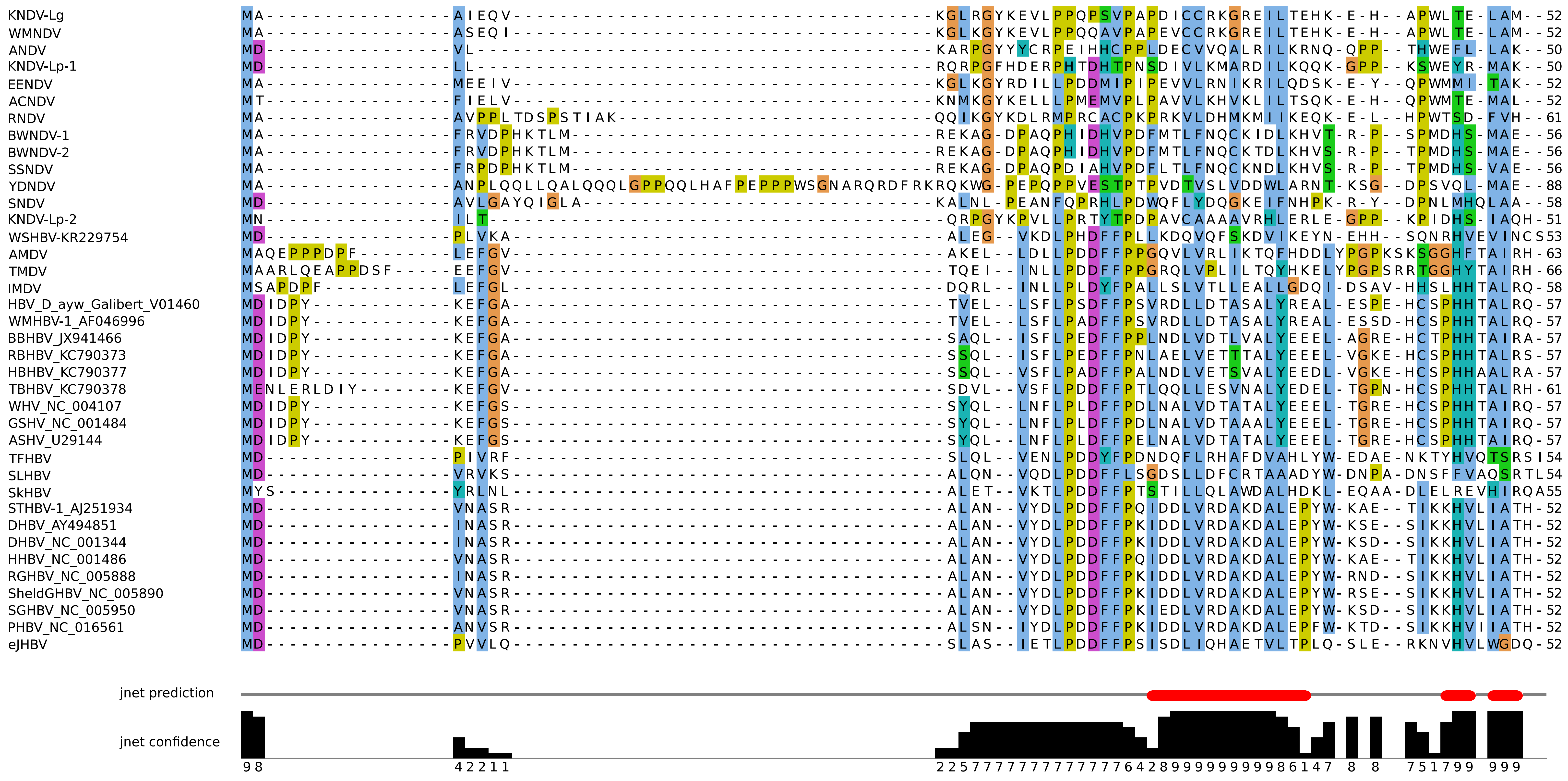
KNDV-Lg
WMNDV
ANDV
KNDV-Lp-1
EENDV
ACNDV
RNDV
BWNDV-1
BWNDV-2
SSNDV
YDNDV
SNDV
KNDV-Lp-2
WSHBV-KR229754
AMDV
TMDV
IMDV
HBV_D_ayw_Galibert_V01460
WMHBV-1_AF046996
BBHBV_JX941466
RBHBV_KC790373
HBHBV_KC790377
TBHBV_KC790378
WHV_NC_004107
GSHV_NC_001484
ASHV_U29144
TFHBV
SLHBV
SkHBV
STHBV-1_AJ251934
DHBV_AY494851
DHBV_NC_001344
HHBV_NC_001486
RGHBV_NC_005888
SheldGHBV_NC_005890
SGHBV_NC_005950
PHBV_NC_016561
eJHBV
jnet prediction
jnet confidence

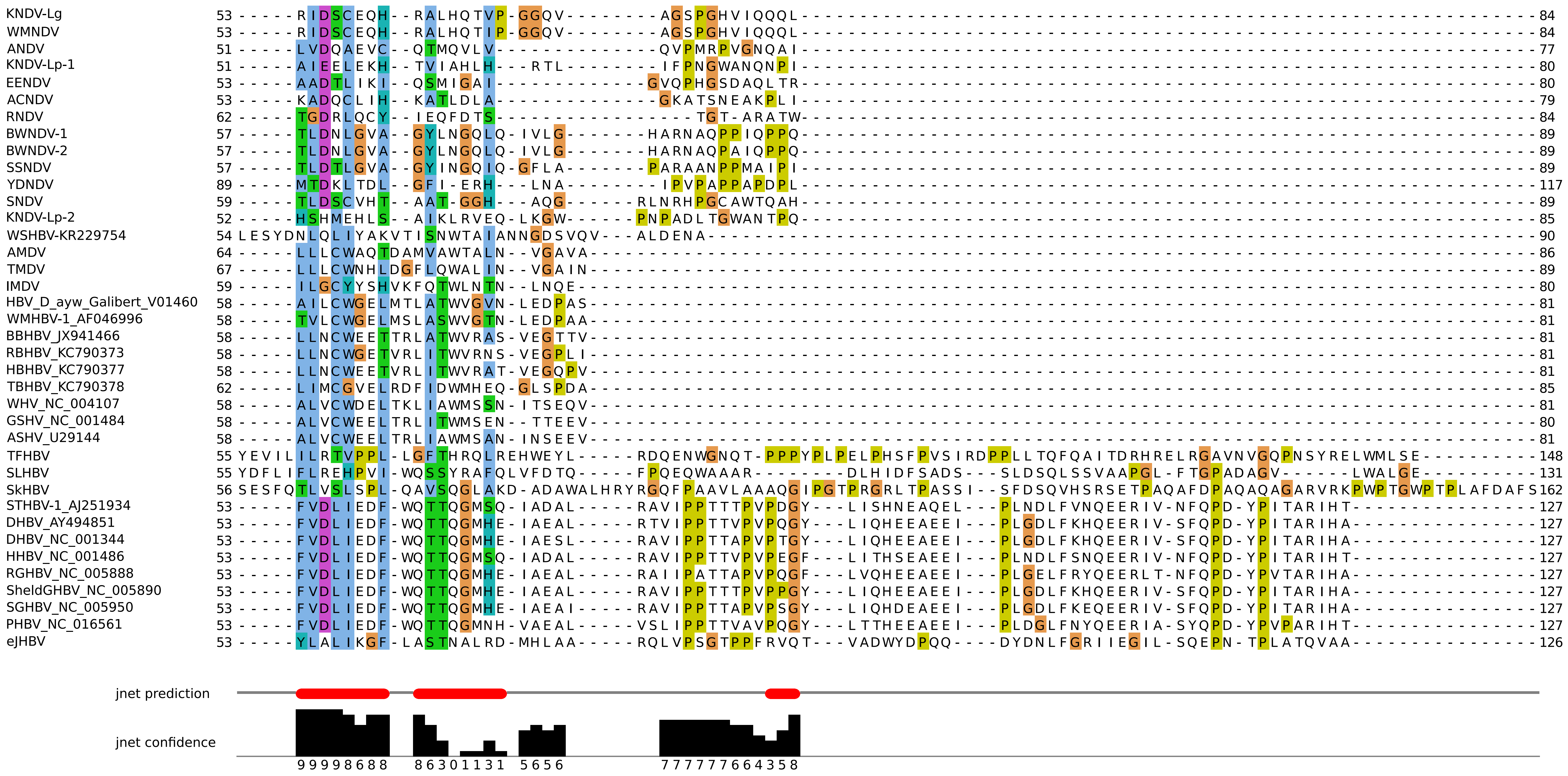

KNDV-Lg
WMNDV
ANDV
KNDV-Lp-1
EENDV
ACNDV
RNDV
BWNDV-1
BWNDV-2
SSNDV
YDNDV
SNDV
KNDV-Lp-2
WSHBV-KR229754
AMDV
TMDV
IMDV
HBV_D_ayw_Galibert_V01460
WMHBV-1_AF046996
BBHBV_JX941466
RBHBV_KC790373
HBHBV_KC790377
TBHBV_KC790378
WHV_NC_004107
GSHV_NC_001484
ASHV_U29144
TFHBV
SLHBV
SkHBV
STHBV-1_AJ251934
DHBV_AY494851
DHBV_NC_001344
HHBV_NC_001486
RGHBV_NC_005888
SheldGHBV_NC_005890
SGHBV_NC_005950
PHBV_NC_016561
eJHBV
jnet prediction
jnet confidence
99998688
86301131
5656
777777664358

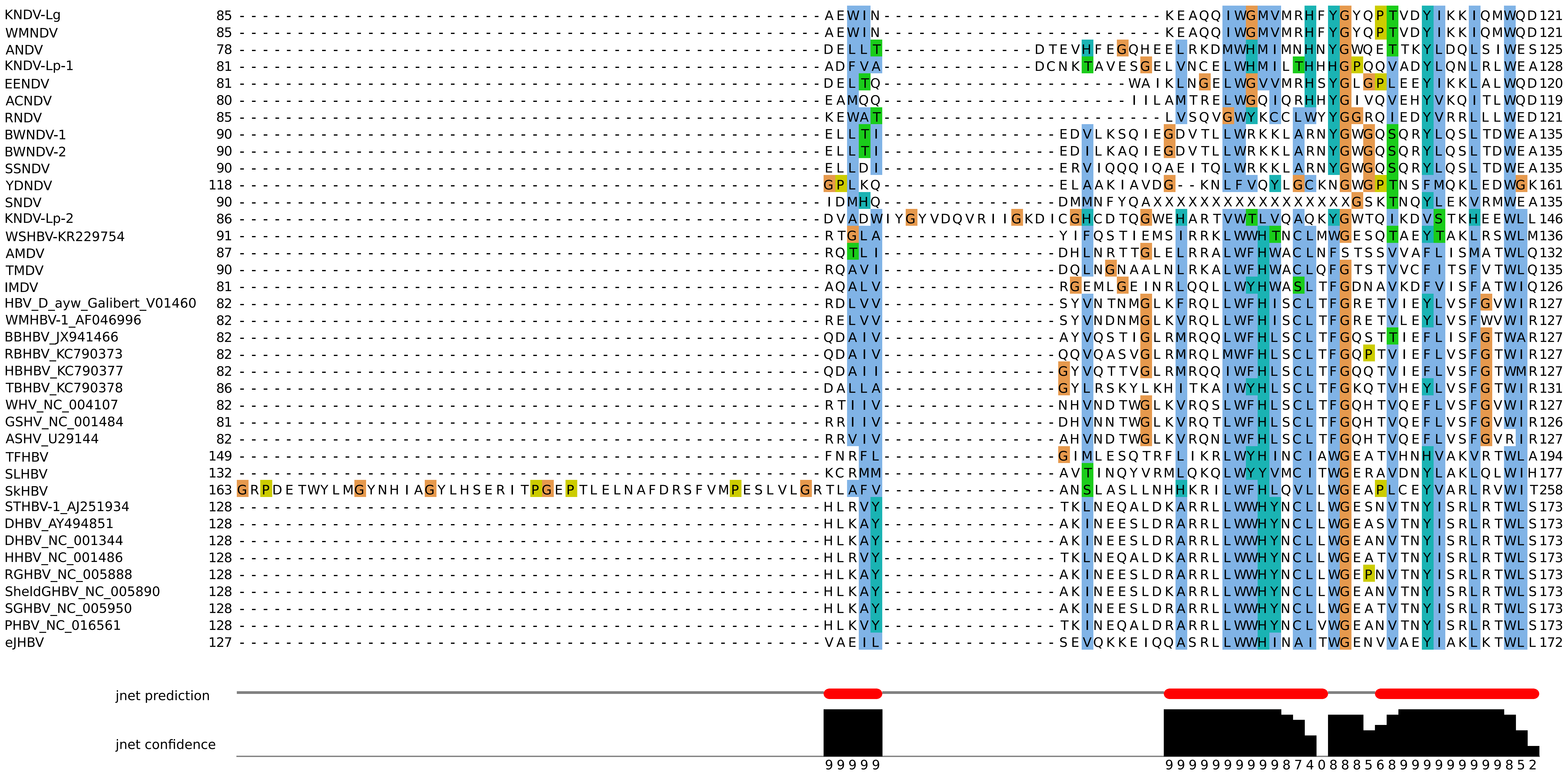
KNDV-Lg
WMNDV
ANDV
KNDV-Lp-1
EENDV
ACNDV
RNDV
BWNDV-1
BWNDV-2
SSNDV
YDNDV
SNDV
KNDV-Lp-2
WSHBV-KR229754
AMDV
TMDV
IMDV
HBV_D_ayw_Galibert_V01460
WMHBV-1_AF046996
BBHBV_JX941466
RBHBV_KC790373
HBHBV_KC790377
TBHBV_KC790378
WHV_NC_004107
GSHV_NC_001484
ASHV_U29144
TFHBV
SLHBV
SkHBV
STHBV-1_AJ251934
DHBV_AY494851
DHBV_NC_001344
HHBV_NC_001486
RGHBV_NC_005888
SheldGHBV_NC_005890
SGHBV_NC_005950
PHBV_NC_016561
eJHBV
jnet prediction
jnet confidence

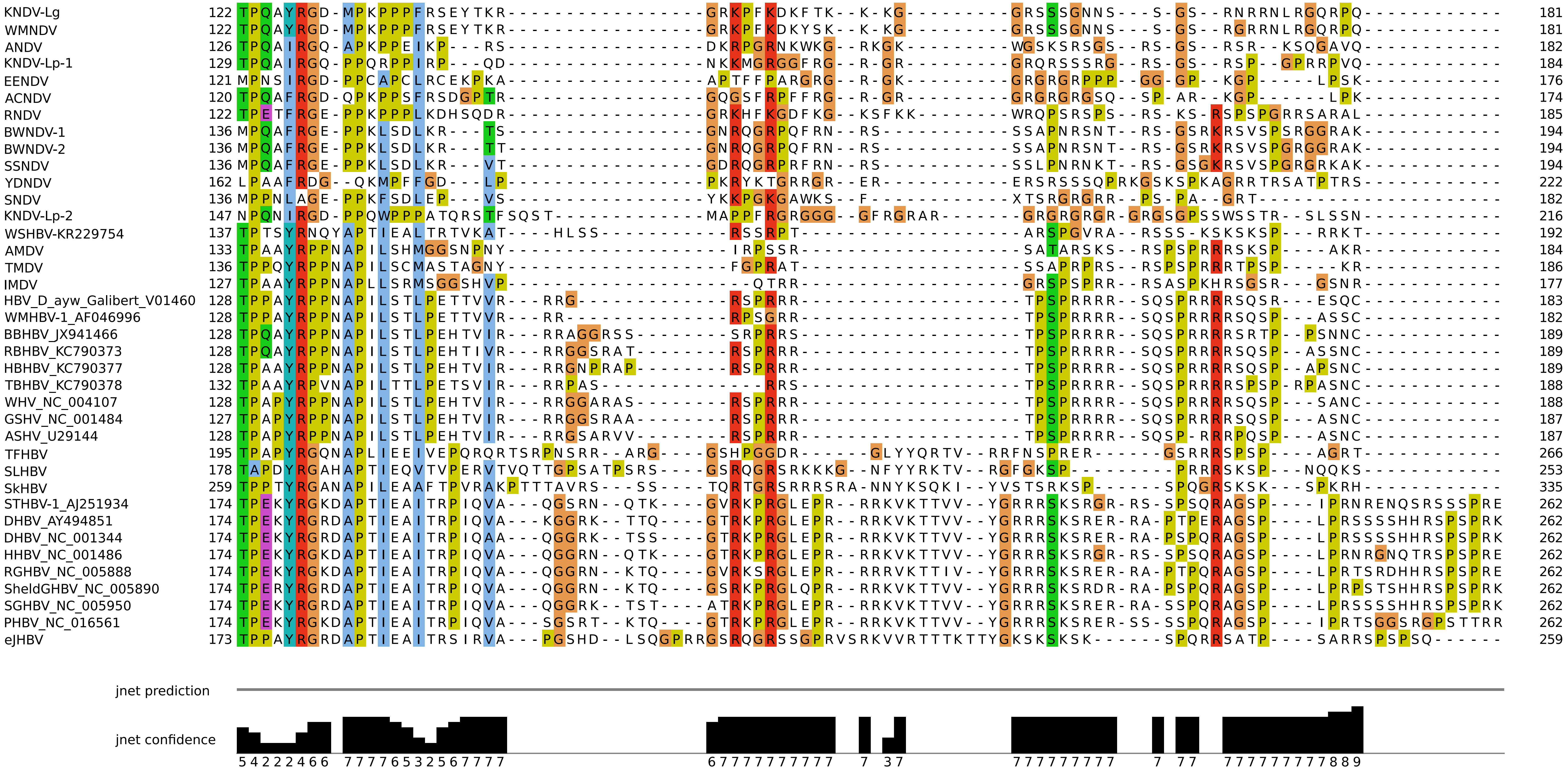

KNDV-Lg 122 TPQAYRGD-MPKPPPFRSEYTKR-----------------GRKPFKDKFTK--K-KG---------GRSSSGNNS---S-GS--RNRRNLRGQRPQ----------- 181
WMNDV 122 TPQAYRGD-MPKPPPFRSEYTKR-----------------GRKPFKDKYSK--K-KG---------GRSSSGNNS---S-GS--RGRRNLRGQRPQ----------- 181
ANDV 126 TPQAIRGQ-APKPPEIKP---RS-----------------DKRPGRNKWKG--RKGK---------WGSKSRSGS--RS-GS--RSR--KSQGAVQ----------- 182
KNDV-Lp-1 129 TPQAIRGQ-PPQRPPIRP---QD-----------------NKKMGRGGFRG--R-GR---------GRQRSSSRG--RS-GS--RSP--GPRRPVQ----------- 184
EENDV 121 MPNSIRGD-PPCAPCLRCEKPKA-----------------APTFFPARGRG--R-GK---------GRGRGRPPP--GG-GP--KGP----LPSK----------- 176
ACNDV 120 TPQAFRGD-QPKPPSFRSDGPTR-----------------GQGSFRPFFRG--R-GR---------GRGRGRGSQ--SP-AR--KGP------LPK----------- 174
RNDV 122 TPETFRGE-PPKPPPLKDHSQDR-----------------GRKHFKGDFKG--KSFKK--------WRQPSRSPS--RS-KS-RSPSPGRRSARAL----------- 185
BWNDV-1 136 MPQAFRGE-PPKLSDLKR---TS-----------------GNRQGRPQFRN--RS---------------SSAPNRSNT--RS-GSRKRSVSPSRGGRAK----------- 194
BWNDV-2 136 MPQAFRGE-PPKLSDLKR---TT-----------------GNRQGRPQFRN--RS---------------SSAPNRSNT--RS-GSRKRSVSPGRGGRAK----------- 194
SSNDV 136 MPQAFRGE-PPKLSDLKR---VT-----------------GDRQGRPRFRN--RS---------------SSLPNRNKT--RS-GSGKRSVSPGRGRKAK----------- 194
YDNDV 162 LPAAFRDG--QKMPFFGD---LP-----------------PKRYKTGRRGR--ER---------------ERSRSSSQPRKGSKSPKAGRRTRSATPTRS----------- 222
SNDV 136 MPPNLAGE-PPKFSDLEP---VS-----------------YKKPGKGAWKS--F---------------XTSRGRGRR--PS-PA--GRT----------------- 182
KNDV-Lp-2 147 NPQNIRGD-PPQWPPPATQRSTFSQST-------------MAPPFRGRGGG--GFRGRAR------GRGRGRGR-GRGSGPSSWSSTR--SLSSN----------- 216
WSHBV-KR229754 137 TPTSYRNQYAPTIEALTRTVKAT----HLSS--------------RSSRPT----------------------ARSPGVRA-RSSS-KSKSKSP---RRKT----------- 192
AMDV 133 TPAAYRPPNAPILSHMGGSNPNY-----------------IRPSSR--------------------------SATARSKS--RSPSPRRRSKSP----AKR----------- 184
TMDV 136 TPPQYRPPNAPILSCMASTAGNY-----------------FGPRAT--------------------------SSAPRPRS--RSPSPRRRTPSP----KR----------- 186
IMDV 127 TPAAYRPPNAPLLSRMSGGSHVP--------------------QTRR------------------------GRSPSPRR--RSASPKHRSGSR---GSNR----------- 177
HBV_D_ayw_Galibert_V01460 128 TPPAYRPPNAPILSTLPETTVVR---RRG--------------RSPRRR-----------------------TPSPRRRR--SQSPRRRRSQSR---ESQC----------- 183
WMHBV-1_AF046996 128 TPPAYRPPNAPILSTLPETTVVR---RR---------------RPSGRR-----------------------TPSPRRRR--SQSPRRRRSQSP---ASSC----------- 182
BBHBV_JX941466 128 TPQAYRPPNAPILSTLPEHTVIR---RRAGGRSS---------SRPRRS-----------------------TPSPRRRR--SQSPRRRRSRTP--PSNNC----------- 189
RBHBV_KC790373 128 TPQAYRPPNAPILSTLPEHTIVR---RRGGSRAT---------RSPRRR-----------------------TPSPRRRR--SQSPRRRRSQSP--ASSNC----------- 189
HBHBV_KC790377 128 TPAAYRPPNAPILSTLPEHTVIR---RRGNPRAP---------RSPRRR-----------------------TPSPRRRR--SQSPRRRRSQSP--APSNC----------- 189
TBHBV_KC790378 132 TPAAYRPVNAPILTTLPETSVIR---RRPAS---------------RRS-----------------------TPSPRRRR--SQSPRRRRSPSP-RPASNC----------- 188
WHV_NC_004107 128 TPAPYRPPNAPILSTLPEHTVIR---RRGGARAS---------RSPRRR-----------------------TPSPRRRR--SQSPRRRRSQSP---SANC----------- 188
GSHV_NC_001484 127 TPAPYRPPNAPILSTLPEHTVIR---RRGGSRAA---------RSPRRR-----------------------TPSPRRRR--SQSPRRRRSQSP---ASNC----------- 187
ASHV_U29144 128 TPAPYRPPNAPILSTLPEHTVIR---RRGSARVV---------RSPRRR-----------------------TPSPRRRR--SQSP-RRRPQSP---ASNC----------- 187
TFHBV 195 TPAPYRGQNAPLIEEIVEPQRQRTSRPNSRR--ARG---GSHPGGDR------GLYYQRTV--RRFNSPRER------GSRRRSPSP---AGRT----------- 266
SLHBV 178 TAPDYRGAHAPTIEQVTVPERVTVQTTGPSATPSRS---GSRQGRSRKKKG--NFYYRKTV--RGFGKSP----------PRRRSKSP---NQQKS----------- 253
SkHBV 259 TPPTYRGANAPILEAAFTPVRAKPTTTAVRS---SS---TQRTGRSRRRSRA-NNYKSQKI--YVSTSRKSP------SPQGRSKSK---SPKRH----------- 335
STHBV-1_AJ251934 174 TPEKYRGKDAPTIEAITRPIQVA---QGSRN--QTK---GVRKPRGLEPR--RRKVKTTVV--YGRRRSKSRGR-RS-SPSQRAGSP----IPRNRENQSRSSSPRE 262
DHBV_AY494851 174 TPEKYRGRDAPTIEAITRPIQVA---KGGRK--TTQ---GTRKPRGLEPR--RRKVKTTVV--YGRRRSKSRER-RA-PTPERAGSP----LPRSSSSHHRSPSPRK 262
DHBV_NC_001344 174 TPEKYRGRDAPTIEAITRPIQAA---QGGRK--TSS---GTRKPRGLEPR--RRKVKTTVV--YGRRRSKSRER-RA-PSPQRAGSP----LPRSSSSHHRSPSPRK 262
HHBV_NC_001486 174 TPEKYRGKDAPTIEAITRPIQVA---QGGRN--QTK---GTRKPRGLEPR--RRKVKTTVV--YGRRRSKSRGR-RS-SPSQRAGSP----LPRNRGNQTRSPSPRE 262
RGHBV_NC_005888 174 TPEKYRGKDAPTIEAITRPIQVA---QGGRN--KTQ---GVRKSRGLEPR--RRRVKTTIV--YGRRRSKSRER-RA-PTPQRAGSP----LPRTSRDHHRSPSPRE 262
SheldGHBV_NC_005890 174 TPERYRGRDAPTIEAITRPIQVA---QGGRN--KTQ---GSRKPRGLQPR--RRKVKTTVV--YGRRRSKSRDR-RA-PSPQRAGSP----LPRPSTSHHRSPSPRK 262
SGHBV_NC_005950 174 TPEKYRGRDAPTIEAITRPIQVA---QGGRK--TST---ATRKPRGLEPR--RRKVKTTVV--YGRRRSKSRER-RA-SSPQRAGSP----LPRSSSSHHRSPSPRK 262
PHBV_NC_016561 174 TPEKYRGKDAPTIEAITRPIQVA---SGSRT--KTQ---GTRKPRGLEPR--RRKVKTTVV--YGRRRSKSRER-SS-SSPQRAGSP----IPRTSGGSRGPSTTRR 262
eJHBV 173 TPPAYRGRDAPTIEAITRSIRVA---PGSHD--LSQGPRRGSRQGRSSGPRVSRKVVRTTTKTTYGKSKSKSK------SPQRRSATP----SARRSPSPSQ------ 259
jnet prediction
jnet confidence
5422224 66 777765325677777 67777777777 7 37 777777777 7 77 777777777889
