# Supplementary material for: Deciphering the Origin and Evolution of Hepatitis B Viruses by Means of a Family of Non-enveloped Fish Viruses
Source: Cell Host Microbe. 2017 Sep 13;22(3):387–399.e6. doi: 10.1016/j.chom.2017.07.019 (PMC5604429; doi:10.1016/j.chom.2017.07.019)
Supplement: Document S2. Article plus Supplemental Information [file mmc8.pdf]

Cell Host & Microbe

Deciphering the Origin and Evolution of Hepatitis B Viruses by Means of a Family of Non-enveloped Fish Viruses

Graphical Abstract

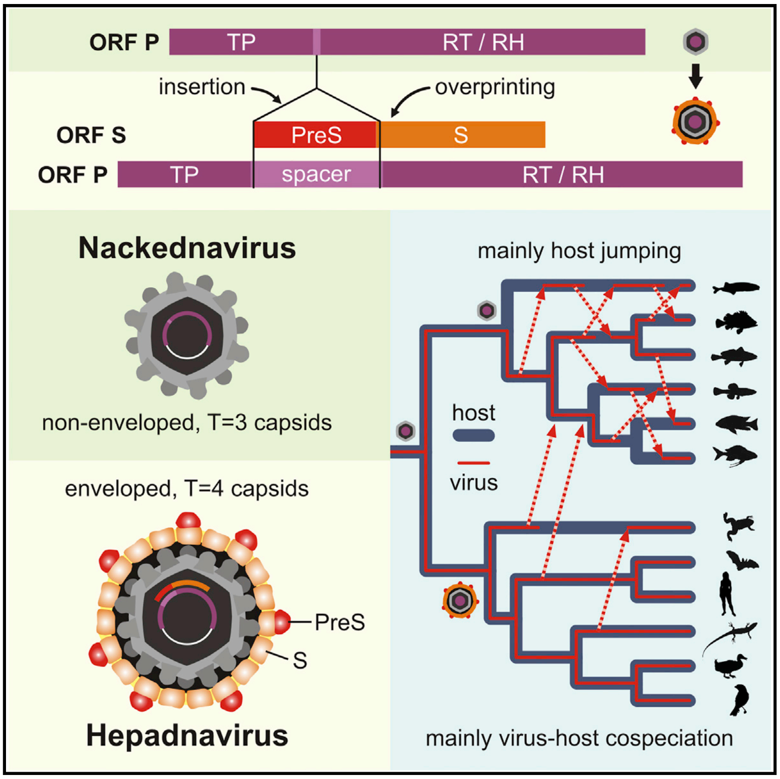

Highlights

- Nackednaviruses are non-enveloped fish viruses related to hepadnaviruses
- Both virus families separated from a common ancestor >400 million years ago
- The envelope protein gene of hepadnaviruses emerged through two distinct processes
- Hepadnaviruses mainly co-evolve with hosts while nackednaviruses jump between hosts

Authors

Chris Lauber, Stefan Seitz, Simone Mattei, ..., Lars Kaderali, John A.G. Briggs, Ralf Bartenschlager

Correspondence

stefan.seitz@med.uni-heidelberg.de

In Brief

Hepatitis B viruses are enveloped viruses of global medical importance. Lauber et al. report the discovery of nackednaviruses, a non-enveloped sister family to hepatitis B viruses in fish. Both lineages separated >400 million years ago. The envelope gene of hepatitis B viruses emerged *de novo*, followed by virus-host co-evolution over geologic eras.

Lauber et al., 2017, Cell Host & Microbe *22*, 387–399
September 13, 2017 © 2017 The Author(s). Published by Elsevier Inc.
http://dx.doi.org/10.1016/j.chom.2017.07.019

Cell Host & Microbe
Article

Deciphering the Origin and Evolution of Hepatitis B Viruses by Means of a Family of Non-enveloped Fish Viruses

Chris Lauber,[1,10] Stefan Seitz,[2,10,11,*] Simone Mattei,[3] Alexander Suh,[4] Jürgen Beck,[5] Jennifer Herstein,[6] Jacob Börold,[2] Walter Salzburger,[7] Lars Kaderali,[1,8] John A.G. Briggs,[3] and Ralf Bartenschlager[2,9]
[1]Institute for Medical Informatics and Biometry, Technische Universität Dresden, 01307 Dresden, Germany
[2]University of Heidelberg, Department of Infectious Diseases, Molecular Virology, 69120 Heidelberg, Germany
[3]Structural and Computational Biology Unit, European Molecular Biology Laboratory, 69117 Heidelberg, Germany
[4]Department of Evolutionary Biology, Evolutionary Biology Centre (EBC), Uppsala University, 75236 Uppsala, Sweden
[5]Department of Internal Medicine 2/Molecular Biology, University Hospital Freiburg, 79106 Freiburg, Germany
[6]Department of Psychiatry and the Behavioral Sciences, Keck School of Medicine, University of Southern California, Los Angeles, CA 90033, USA
[7]Zoological Institute, University of Basel, 4051 Basel, Switzerland
[8]Institute for Bioinformatics, University Medicine Greifswald, 17487 Greifswald, Germany
[9]Division of Virus-Associated Carcinogenesis, German Cancer Research Center (DKFZ), 69120 Heidelberg, Germany
[10]These authors contributed equally
[11]Lead Contact
*Correspondence: stefan.seitz@med.uni-heidelberg.de
http://dx.doi.org/10.1016/j.chom.2017.07.019

SUMMARY

Hepatitis B viruses (HBVs), which are enveloped viruses with reverse-transcribed DNA genomes, constitute the family *Hepadnaviridae*. An outstanding feature of HBVs is their streamlined genome organization with extensive gene overlap. Remarkably, the ~1,100 bp open reading frame (ORF) encoding the envelope proteins is fully nested within the ORF of the viral replicase P. Here, we report the discovery of a diversified family of fish viruses, designated nackednaviruses, which lack the envelope protein gene, but otherwise exhibit key characteristics of HBVs including genome replication via protein-primed reverse-transcription and utilization of structurally related capsids. Phylogenetic reconstruction indicates that these two virus families separated more than 400 million years ago before the rise of tetrapods. We show that HBVs are of ancient origin, descending from non-enveloped progenitors in fishes. Their envelope protein gene emerged *de novo*, leading to a major transition in viral lifestyle, followed by co-evolution with their hosts over geologic eras.

INTRODUCTION

Hepatitis B virus (HBV) is a human pathogen of global importance that has infected around two-fifths of the world population. At least 250 million people are chronic HBV carriers living at high risk of developing liver cirrhosis and hepatocellular carcinoma (WHO, 2017; Yang and Roberts, 2010). HBV infections account for ~890,000 deaths annually (WHO, 2017). HBV represents the prototype member of *Hepadnaviridae*, a family of small enveloped DNA viruses (Seeger and Mason, 2000). Their ~3.2 kb circular genomes are reverse-transcribed from an RNA intermediate by the viral P protein (Seeger and Mason, 2000; Beck and Nassal, 2007). DNA synthesis is initiated by a unique priming mechanism involving the covalent attachment of the first nucleotide to the terminal protein domain (TP) of P and proceeds by the action of the reverse transcriptase (RT) and RNase H (RH) domains, which are separated from TP by a flexible spacer region (Figure 1A) (Bartenschlager and Schaller, 1988; Beck and Nassal, 2007). This complex replication process takes place within the viral capsids. Consequently, the genomes cannot expand in size beyond a certain upper limit (Chirico et al., 2010). Hepadnaviruses overcome this constraint partly by increasing information density through extensive gene overlap. The most peculiar feature in this regard is the open reading frame (ORF) for the envelope glycoproteins (PreS/S), which spans >1.1 kb and lies completely within the P gene, but is frameshifted downstream by one nucleotide (Figures 1A and 1E). The sequence coding for the N-terminal PreS domains corresponds to that for the spacer region in P. The S coding sequence overlaps with the essential part of the RT domain. Such gene overlaps typically evolve through a process called "overprinting," i.e., the emergence of a novel ORF within the coding sequence of a pre-existing ancestral gene (Keese and Gibbs, 1992; Pavesi et al., 2013).

Until recently, hepadnaviruses were only known from mammals (genus *Orthohepadnavirus*) and birds (genus *Avihepadnavirus*) (Schaefer, 2007). Previous age estimates for the split between both genera ranged from 30,000 to 125,000 years before the present time, and the divergence of species within the mammalian virus clade was proposed to have occurred 10,000 to 25,000 years ago (Orito et al., 1989; Mizokami and Orito, 1999; van Hemert et al., 2011). The discovery of endogenous

This is an open access article under the CC BY license (http://creativecommons.org/licenses/by/4.0/).

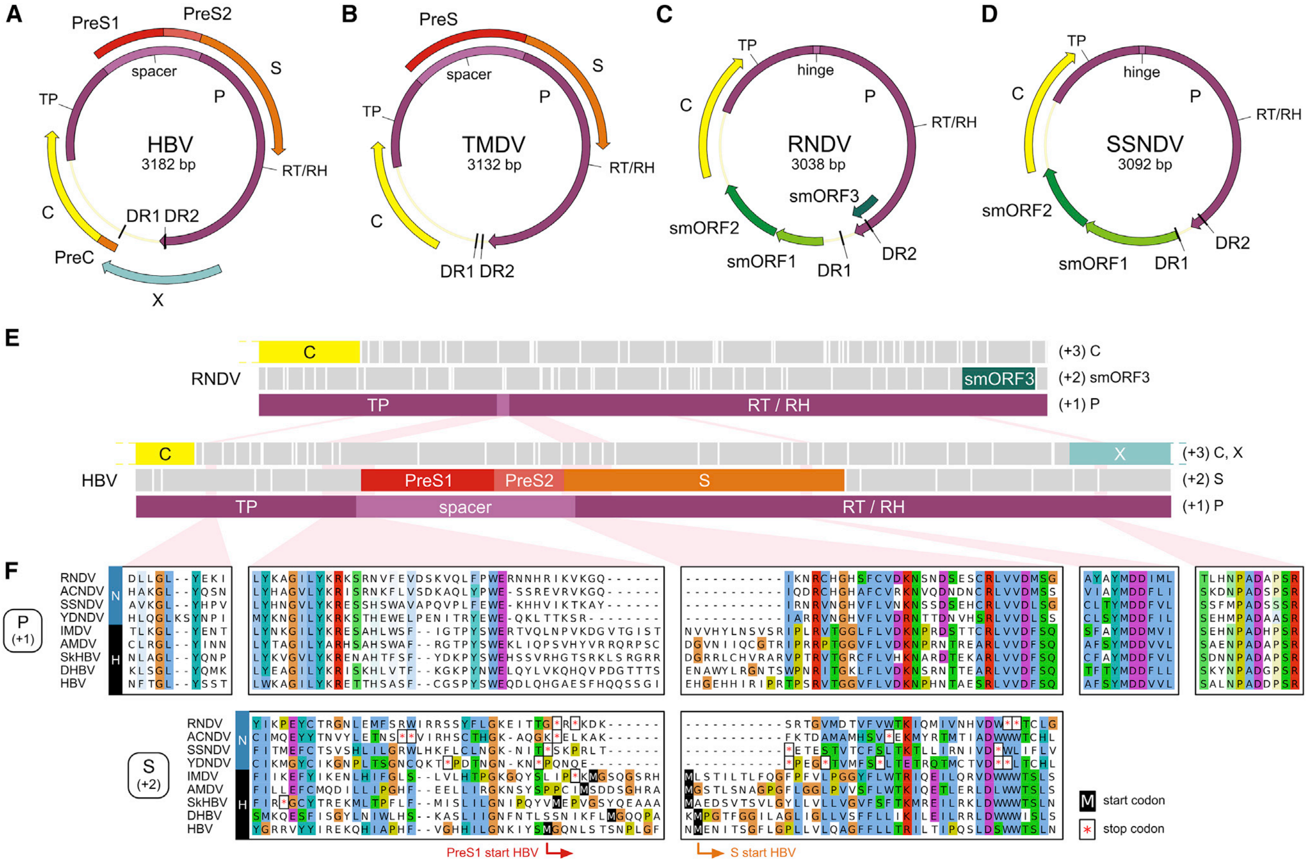

Figure 1. Genome Organization of Hepadna- and Nackednaviruses

(A) Human hepatitis B virus (HBV).

(B) Tetra metahepadnavirus (TMDV) of the Mexican tetra (*Astyanax mexicanus*). An ORF X is absent.

(C) Rockfish nackednavirus (RNDV).

(D) Sockeye salmon nackednavirus (SSNDV).

(E) Comparison of the RNDV and HBV P ORF. All three reading frames are depicted (+1, +2, +3). White vertical bars: stop codons. TP, terminal protein; RT, reverse transcriptase; RH, RNaseH.

(F) Amino acid sequence alignments of selected parts of P (+1) and S (+2) reading frames, including four representatives of nackednaviruses (N) and five of hepadnaviruses (H). Nackednaviruses harbor multiple stop codons in the region of the (+2) frame corresponding to the hepadnaviral RT/S overlap.

See also Table S1 and Figures S1 and S2.

hepadnaviruses in the genomes of birds (Gilbert and Feschotte, 2010; Liu et al., 2012; Suh et al., 2013), crocodilians, turtles, and snakes (Gilbert et al., 2014; Suh et al., 2014) has shifted the absolute age estimate for the entire virus family substantially into the past, since these endogenization events occurred up to 231 million years ago (mya) (Suh et al., 2014). The recent identification of several hepadnavirus species in teleost fishes (Hahn et al., 2015; Dill et al., 2016) and an amphibian implied an even more complex, mixed evolutionary pattern assumed to be driven both by virus-host cospeciation events and cross-species transmissions (Dill et al., 2016; Geoghegan et al., 2017). Hence, the origin of HBVs remains enigmatic and, as yet, no conclusive phylogenetic hypothesis of *Hepadnaviridae* exists: it is unknown when and how they became enveloped, diversified into separate lineages, and spread among tetrapods. Here, we describe a family of non-enveloped (naked), HBV-related fish viruses, allowing us to trace the evolutionary history of hepadnaviruses to a root more than 400 mya.

RESULTS

Nackednaviruses Are Non-enveloped HBV-Related Viruses of Teleost Fishes

We identified HBV-related viruses by homology searching in public sequence databases at the National Center for Biotechnology Information (NCBI). We used the protein sequence of the TP domain as the search query, since it is unique to these viruses. Among the screened data were >25,000 entries of bony fishes in the Sequence Read Archive (SRA). By this means, we retrieved 17 complete or nearly complete genome sequences of exogenous HBV-related viruses in teleost fishes (synopsis in Table S1, genome maps in Figure S1, annotated sequences in Data S1). Notably, these viruses are present in a wide variety of tissues and do not exhibit a marked liver tropism (Table S1). Furthermore, we discovered full genomes of exogenous hepadnaviruses in the skink *Saproscincus basiliscus* (SkHBV) and the spiny lizard *Sceloporus adleri* (SLHBV-1), as well as an

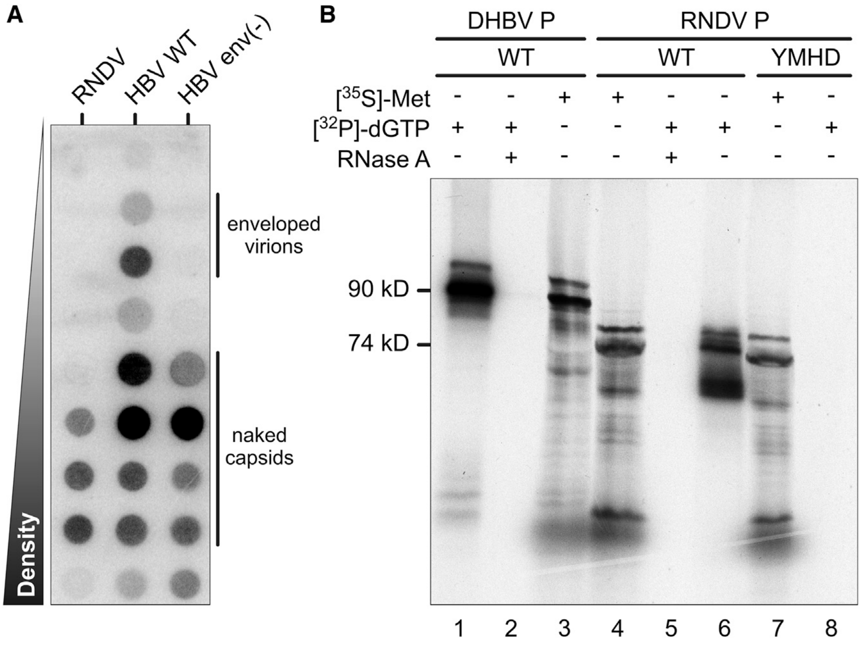

Figure 2. Virological Assays

(A) Release of naked capsids from RNDV-transfected cells. HuH-7 cells were transfected with expression plasmids containing terminally redundant genomes of RNDV, HBV, or HBV env(−), an envelope protein-deficient HBV mutant. Cell culture supernatants were subjected to CsCl density gradient centrifugation followed by detection of viral DNA in gradient fractions by DNA-dot blot hybridization. Similar results were obtained in the HEK cell line HEK293T and in the rainbow trout gonad cell line RTG-2 (data not shown).

(B) P priming assays. RNDV and duck hepatitis B virus (DHBV) wild-type (WT) P proteins produced in a coupled *in vitro* transcription/translation system were incubated with [α-^{32}P]dGTP and subjected to SDS-PAGE followed by autoradiography (lanes 1 and 6). To demonstrate template dependency of the priming reaction, RNase A digests were performed prior to incubation with [α-^{32}P]dGTP (lanes 2 and 5). An RNDV YMDD-motif mutant in P was included to show dependency of the priming reaction on an intact RT domain (YMHD; lane 8). As control for proper protein production, P proteins were metabolically radiolabeled with [^{35}S]methionine without addition of [α-^{32}P]dGTP (lanes 3, 4, and 7).

actively transcribed endogenous viral element in the dark-eyed Junco (eJHBV), a North American sparrow (*Junco hyemalis*) (Table S1; Figure S2).

As exemplified by the Mexican tetra metahepadnavirus (TMDV) (termed after its position in the viral phylogeny; see below), four of the piscine viruses display the typical genome structure with an envelope protein ORF, which is completely overlapped by ORF P and shows the characteristic bipartition into PreS and S regions (Figure 1B). An ORF X encoding a transactivator as in mammalian HBVs is absent.

Owing to their peculiar genome organization, the 13 remaining fish viruses constitute a distinct group that we termed nackednaviruses (Swabian German for "naked DNA viruses"). The genome sizes within this group range from 2,766 to 3,105 bp (Figure S1). As exemplified with the rockfish nackednavirus (RNDV) (Figure 1C) and the sockeye salmon nackednavirus (SSNDV) (Figure 1D), the circular genomes comprise two partially overlapping, major ORFs, encoding for a Core (C) and a P protein, the latter being composed of TP, RT, and RH domains. Two short direct repeats (DR1 and DR2), essential for replication in hepadnaviruses (Beck and Nassal, 2007), are located in the non-translated region and within the 3′ region of the RH domain. Between DR1 and ORF C, all nackednaviruses contain two small ORFs (Figure S1). In sharp contrast to hepadnaviruses, a PreS/S ORF for envelope proteins is missing (Figures 1C and 1D).

The TP, RT, and RH domains of the P proteins are homologous to the hepadnaviral counterparts, with an average degree of sequence similarity <50% between both groups (Figures 1E and 1F). Notably, all functionally important motifs are conserved (Figure 1F; full alignment in Data S2 and S3). In nackednaviruses, however, TP is directly linked to RT via a short hinge region (Figure 1E). They lack a long spacer that, in the case of hepadnaviruses, encodes the PreS domains of the envelope proteins in its second reading frame. In the region of the RT/S overlap of hepadnaviruses, all nackednaviruses contain multiple stop codons in the S-congruent reading frame (Figures 1E and 1F).

None of the genomes of the non-enveloped or enveloped fish viruses, respectively, features signs indicative of endogenous viral elements, e.g., flanking host genome sequences, inactivating frameshift mutations or premature stop codons. To date, we have not detected any example of endogenized HBV-related viral sequences in genomes of teleost fishes.

Nackednaviruses Are Replication-Competent Exogenous Viruses

Some nackednavirus genomes were retrieved from transcriptome-sequencing projects indicating active transcription of poly(A)-tailed full-length viral RNA in infected fish (Table S1). To elucidate replication competence, we synthesized the complete genome sequence of RNDV and inserted it into a eukaryotic expression vector. We transfected the human hepatoma cell line HuH-7 with this construct and harvested the culture supernatant after 10 days. HBV replicating cells are known to secrete enveloped virions and (via an independent trafficking pathway) also naked capsids (Ni et al., 2010; Bardens et al., 2011). While we detected both particle types in the supernatant of control cells expressing HBV, the cells expressing the RNDV genome exclusively released viral DNA-containing particles with a buoyant density of 1.34–1.45 g/cm^3, corresponding to naked capsids (Figure 2A).

To test RNDV P for the characteristic mode of protein-primed replication initiation, we performed priming assays as established for duck hepatitis B virus (DHBV) (Figure 2B) (Weber et al., 1994). Accordingly, we generated P in a coupled *in vitro* transcription-translation system and offered [α-^{32}P]dGTP as substrate. Full-length RNDV P appeared as a ^{32}P-labeled 74 kDa protein revealing covalent attachment of the nucleotide as marker for protein priming (Figure 2B, lane 6). The enzymatic activity depended on the presence of viral template RNA (Figure 2B, lane 5 versus 6) and required the integrity of the YMDD

motif in the catalytic center of the RT domain (Figure 2B, lane 6 versus 8). Together, these results demonstrate that RNDV is replication-competent and capable of producing non-enveloped extracellular progeny particles. The genome replication mechanism is similar to HBVs in involving protein-primed reverse-transcription of an RNA intermediate.

Ultrastructure of Nackednavirus Capsids

The nackednaviral C proteins showed little sequence similarity with those of hepadnaviruses, and only two regions appeared to be weakly conserved (alignment in Data S4). However, secondary structure predictions revealed the conserved arrangement of α helices characteristic for the C protein of HBV (Wynne et al., 1999), as well as an additional short helix (α+) at the extreme N terminus (Figure 3A).

HBV capsids are spherical particles with a holey shell and protruding spikes (Crowther et al., 1994). The vast majority of HBV capsids display an icosahedral T=4 symmetry (Crowther et al., 1994), while about 5% of the capsid particles are smaller and exhibit T=3 symmetry. We expressed C proteins of the African cichlid nackednavirus (ACNDV) in *E. coli*, purified self-assembled capsids, and performed cryoelectron microscopy (Figures S3A–S3C). The 3D particle reconstruction showed T=3 icosahedral symmetry (Figure 3B, top and middle panel) where the overall fold of the ACNDV C protein was similar to that of HBV (Figure S3D). In contrast to HBV, at the local (pseudo-)3-fold axes the holes in the particle shell were plugged by the additional N-terminal helices, which might aid protecting the genomes of the non-enveloped viruses against environmental damage (Figure 3B, middle and bottom panels).

Long-Term Virus-Host Evolution Patterns

To clarify the phylogenetic relationship between nackednaviruses and hepadnaviruses, we inferred rooted Bayesian trees based on the protein alignment of conserved parts of TP, RT, and RH (alignment in Data S2 and S3; uncalibrated trees in Figure S4). Rooting of the P phylogeny was independently confirmed by an analysis including a set of caulimoviruses and retroviruses as outgroups (Figure S5A). Nackednaviruses demarcated as a well-supported distinct branch constituting a sister taxon to hepadnaviruses (Figure 4; see also C protein phylogeny in Figure S5B). They formed two subgroups, designated RNDV-type and SSNDV-type. A third branch arose with KNDV-Lp-2 currently representing its only member.

In the clade of enveloped viruses, the first diverging lineage (termed parahepadnaviruses) comprised the recently described WSHBV from white sucker (*Catostomus commersonii*) (Hahn et al., 2015) and CSKV from coho salmon (*Oncorhynchus kisutch*) (Figure 4). The three other enveloped fish viruses, TMDV, AMDV (from astatotilapia), and IMDV (from the icefish), appeared as a sister group to the mammalian orthohepadnaviruses. This position was consistent with them having a so-called *a-determinant* (an insertion in the S domain [Glebe and Urban, 2007]) as a synapomorphic character. Hence, we named them metahepadnaviruses (Figure 4). Likewise, the exogenous viruses from the Tibetan frog and lizards (coined herpetohepadnaviruses) formed a separate lineage related to avihepadnaviruses, with which they share enlarged C proteins (alignment in Data S4).

The phylogenetic relatedness of nackednavirus species did not coincide with that of their extant hosts, which hints at frequent host switches across the tree of teleost fishes (Figure 5). Contrarily, the diversification pattern within hepadnaviruses largely reflected that of the respective host taxa, indicating cospeciation as predominant mode of virus-host evolution, although several exceptions were observed (Figure 5). Importantly, we also found a tight matching of the relative distances at those very nodes where the tree topologies are congruent between hepadnaviruses and their hosts (Figure S6A).

To infer divergence times, we included P protein sequences of an endogenous avihepadnaviral element (eAHBV-FRY) integrated in the genomes of Neoaves (Suh et al., 2013) (Figures 4 and S7A). Assuming concomitant diversification, we used the onset of the adaptive radiation of Neoaves (69–67 mya) (Jarvis et al., 2014; Prum et al., 2015; Claramunt and Cracraft, 2015) as the age for the eAHBV-FRY root, thus allowing for dating the other branching points in the virus tree. For nodes representing putative virus-host cospeciation events of exogenous hepadnaviruses (Figure 5), our age estimates were in excellent agreement with the divergence times of the respective host lineages (Figures 6 and S6B). Vice versa, independent calibrations based on the host split ages of these exogenous viruses yielded a mean age of 67.9 mya (± 13.6 mya SD) for the eAHBV-FRY node (Figure 6). Notably, with both calibration strategies we observed a tight and statistically well-supported congruence of the mutual divergence times in the investigated virus-host pairings with congruent tree topologies (Figure 6). Similar results were obtained in tree calibrations based on the age of an endogenous hepadnaviral element in the genomes of snakes (Figure S7B). These findings are remarkable, since they hint at comparable long-term substitution rates for exogenous and endogenized viral P protein sequences, thus implying synchronous evolution of hepadnaviruses with their hosts, probably for more than 400 million years.

De Novo Emergence of the PreS/S ORF in the Hepadnaviral Lineage

The most prominent difference between nackednaviruses and hepadnaviruses is the absence or presence of an envelope protein gene, respectively. Two explanations are possible: either the last common ancestor of both families was a naked virus and PreS/S appeared as an innovation in the hepadnaviral lineage (Pavesi, 2015), or PreS/S evolved in the common ancestry of both families and nackednaviruses lost it secondarily. In the latter instance, one might still find vestiges of a past envelope protein gene imprinted in the genomes of nackednaviruses. Since evolution of the PreS part must have involved an insertion/deletion event precluding comparative analyses (Figure 1E), we analyzed the S part, which overlaps with the RT domain of P. For HBV, the nucleotide variability in this region was described to be reduced at the third codon position of P, which equals codon position 2 of the S ORF (P3/S2) (Zaaijer et al., 2007). We extended this approach to a comprehensive set of viral genomes, including caulimo- and retroviruses as controls, and found the P3/S2 nucleotide variability to be diminished exclusively in the RT/S overlap of hepadnaviruses (Figure 7A). Moreover, hepadnaviruses exhibited a decreased frequency of adenine (A) at position P3/S2 in this region indicating selection

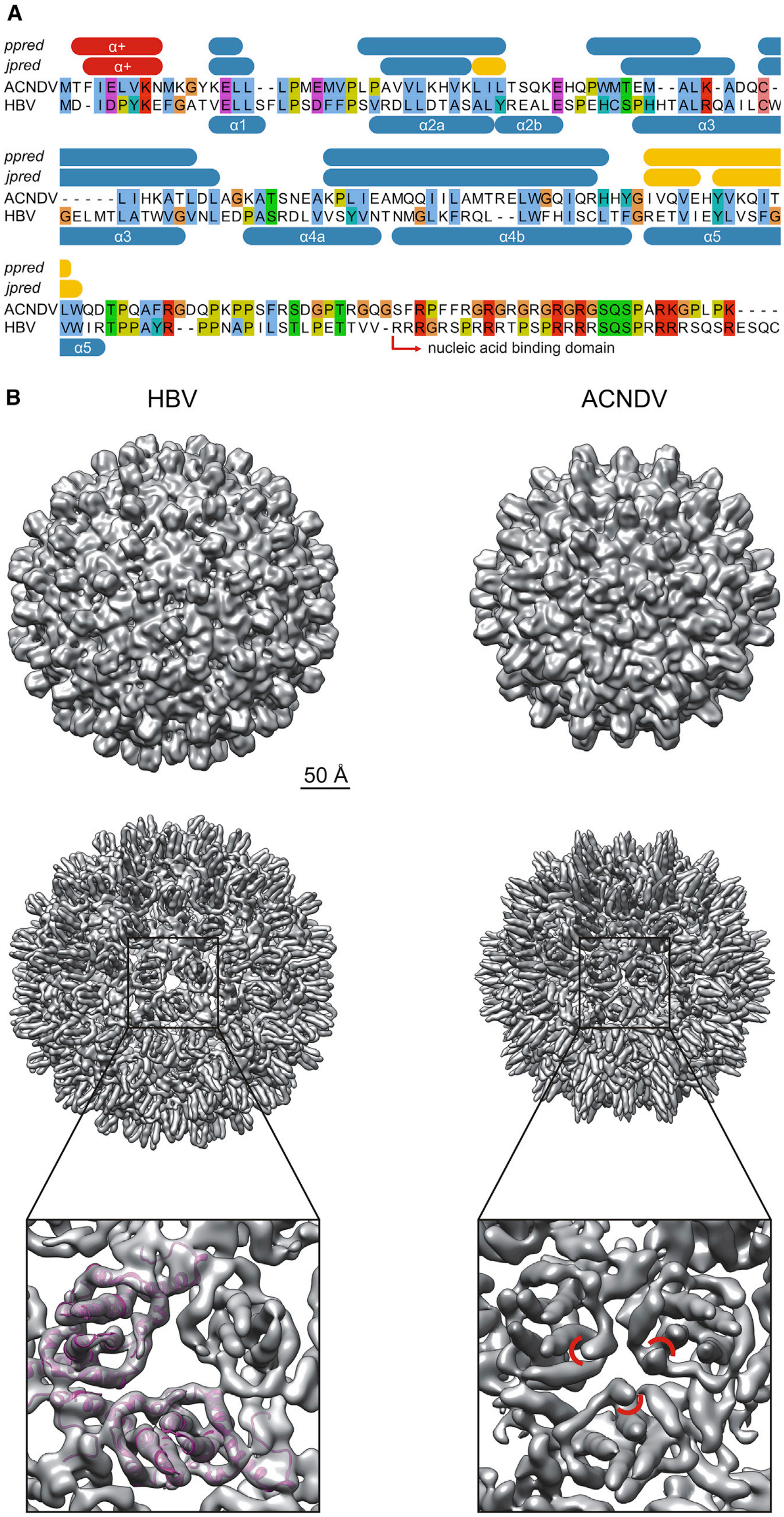

Figure 3. Capsid Ultrastructure

(A) Alignment of the C proteins of African cichlid nackednavirus (ACNDV) and HBV. α helices of HBV C indicated in the bottom refer to the crystal structure (Wynne et al., 1999). Secondary structures of ACNDV C predicted with jpred (Drozdetskiy et al., 2015) and psipred (ppred) (Jones, 1999) are given in the top. Blue, α helices; yellow, β sheets; red, additional, N-terminal α helix (α+).

(B) Comparison of the capsid structure of HBV (T=4) (Yu et al., 2013) and ACNDV (T=3). Cryoelectron microscopy maps low-pass filtered at 12 Å (top row) and 8 Å (middle row). Bottom row: zoomed view onto a local (pseudo-)3-fold axis. Additional α+ helices in ACNDV highlighted by red arcs.

See also Figure S3.

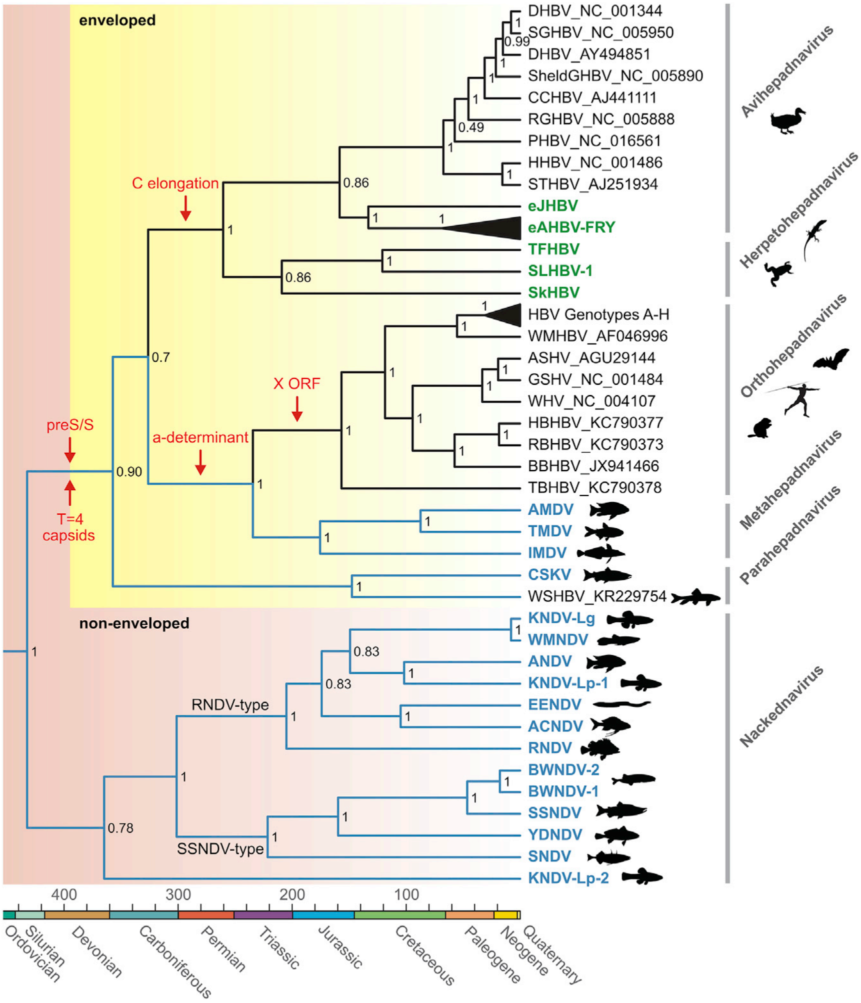

Figure 4. Phylogenetic Relationship of Hepadna- and Nackednaviruses

Rooted Bayesian phylogenetic tree based on protein sequence alignments of conserved regions in the TP, RT, and RH domains of P (437 amino acid positions). For details on parameter optimization of the Bayesian phylogenetic model, see the STAR Methods. Viruses discovered in this study are in color; lineages with piscine hosts in blue. A fourth member of the metahepadnavirus clade was described in a study by Dill et al. (2016). Scale bar, millions of years. Numbers at branching points: posterior probability support values. Red arrows: most parsimonious periods of major evolutionary innovations. The X ORF is an evolutionary novelty of orthohepadnaviruses.

See also Figures S4, S5, and S7 and Tables S2 and S3.

against stop codons in both reading frames (Figure 7B). None of these patterns were observed in nackednaviruses, which resembled the control groups one to one. This exceptional situation in hepadnaviruses supports the more parsimonious model of S gain in this lineage.

To further strengthen this model, we backtracked the evolutionary history of the S ORF by ancestral sequence reconstructions. The inferred hepadnaviral ancestor had an intact S frame, while that of nackednaviruses contained nine stop codons, and the inferred common ancestor of both groups was interrupted by 13 stop codons, indicating that S is an evolutionary novelty of hepadnaviruses (Figure 7C). To corroborate this finding independently, we sought to detect signatures of positive selection in the S-corresponding reading frame of the hepadnaviral ancestral lineage. Since branch lengths in phylogenetic trees quantify the amount of mutational change between two speciation events, we performed a differential tree inference for the two affected frames of the RT/S overlap. We found that the relative evolutionary change in the reading frame corresponding to S is elevated asymmetrically by a factor larger than 2 on the side of hepadnaviruses during the relevant time window after the split from nackednaviruses and before the first intragroup speciation event, while the P frame was under concomitant conservation in both lineages (Figures 7D–7F). In summary, we did not find evidence for a secondary loss of the envelope protein gene in nackednaviruses, but unveiled signs of an extensive adaptation process in the branch leading to hepadnaviruses. This suggests that nackednaviruses most likely retained an ancestral genome organization, while the PreS/S ORF was shaped *de novo* in the hepadnaviral lineage.

DISCUSSION

The peculiar characteristics of the non-enveloped fish viruses described in this study justify assigning them into a distinct virus family apart from hepadnaviruses, and we propose the name "*Nackednaviridae*." They constitute the most suitable outgroup to safely root the phylogeny of their enveloped counterparts for the very first time, enabling us to perform a meticulous cross-examination of the virus-host evolutionary pattern by taking into account both tree topology and relative branch lengths in the respective phylogenies (Figures 5 and S6A). This combined approach permits discerning whether present-day virus-host associations result from cospeciation or involved host switches, even in instances where topology alone is ambiguous. Moreover, the inclusion of an endogenized, "fossil" hepadnaviral element found in the genomes of Neoaves into our taxon sampling allowed for tightly controlled inference of time-calibrated phylogenies, thus retrieving absolute age estimates for viral speciation events (Figures 6 and S6B). According to these analyses, both virus families separated from a common ancestor most likely in the Silurian, ca. 432 mya (Figure 4). This age estimate is in agreement with the separation between ray-finned fishes (Actinopterygii) and lobe-finned fishes (Sarcopterygii, including Tetrapoda) dating back to about 429–425 mya (Betancur et al., 2013; The Timetree of Life, 2016). Consequently, we suggest a virus-host cospeciation event to be most plausible (Figures 5 and 6). The subsequent branch-off of para- and metahepadnaviruses took place about 360 and 240 mya,

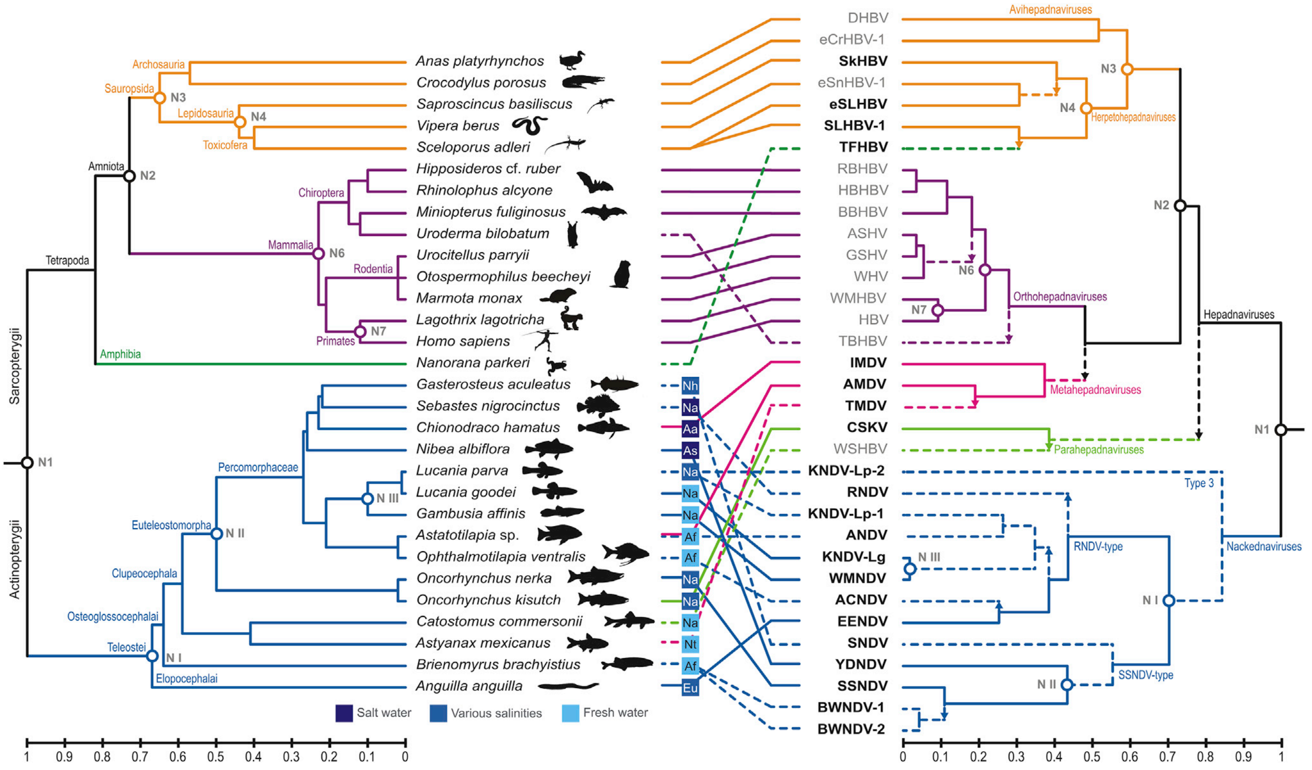

Figure 5. Tanglegram Juxtaposing the Host and Virus Phylogenies

Left panel: ultrametric phylogenetic tree of the host species. Right panel: ultrametric phylogenetic tree of the virus species. Middle panel: virus-host associations. To increase the virus-host spectrum, we included endogenous hepadnaviruses from crocodilians (eCrHBV-1) (Suh et al., 2014), snakes (eSnHBV-1) (Gilbert et al., 2014; Suh et al., 2014), and spiny lizards (eSLHBV). Abbreviations for geographic regions: Aa, Antarctica; Af, Africa; As, Asia; Eu, Europe; Na, North America; Nh, Northern Hemisphere; Nt, Neotropics. Solid lines in the virus tree indicate probable separation of viral daughter lineages due to a virus-host cospeciation event; dashed lines indicate probable virus duplication, i.e., virus speciation predating separation of the extant host lineages; and dashed lines with arrow indicate a host switch and its direction, i.e., virus speciation postdating separation of the extant host lineages. Nodes marked with open circles and labeled with Arabic numerals represent putative cospeciation events that were used in our time-calibration analysis (Figures 6 and S6B). The three putative cospeciation events on the side of nackednaviruses are labeled with Roman numerals (N I—N III).

respectively, indicating independent secondary invasions of actinopterygians through host switches by enveloped viruses that originated on the "sarcopterygian side" of the viral phylogeny (Figures 5, 6, and S6A). This result contradicts the model that hepadnaviruses arose in teleost fishes and colonized mammals and birds much more recently through cross-species transmission, as suggested in a study based on an exclusively cladistic comparison of tree topologies (Geoghegan et al., 2017). Since the mammalian and sauropsid (reptile and bird) hepadnaviruses each belong to ancient lineages, probably with >300 million years of segregated history (Figure 4), the latter ones could have integrated into the genomes of their hosts several times during the last 231 million years (Suh et al., 2014). This intimate association of hepadnaviruses with their hosts since the late Palaeozoic also provided ample time for a fine adaptation, possibly explaining their successfulness in establishing persistent infections that can remain largely asymptomatic in the affected individual for decades. From an evolutionary point of view, the question arises whether HBVs became symbionts, which (apart from their pathogenic potential) also provide advantages to their hosts (Hong et al., 2015).

According to our analyses, the genotypes of human HBV (including the isolates from apes) emerged during the last 30 mya (Figure S7C), coinciding with the origin, radiation, and dispersal of early Hominoidea (= apes) across Africa and Eurasia (Begun, 2003; Springer et al., 2012; Stevens et al., 2013). Since there is no barrier for HBV to be transmitted between humans and extant apes, we suggest that these host taxa represent, from a non-zoologist's but mere virologist's point of view, just slightly different variants of one and the same, unsegregated "host superspecies." In addition to the hypotheses under debate (reviewed in Littlejohn et al., 2016), we therefore propose that HBV might have been freely floating within and between the plethora of stem and crown hominoids whenever these animals (including humans) came into local contact with each other during their complex evolutionary history since the late Oligocene.

The divergence date estimates for nodes on the hepadnaviral branch of the phylogeny differ drastically from previously determined divergence times (see Introduction). These former time inferences were based on molecular clocks in which substitution rate estimates were derived by relating the genetic distances between viral sequences to the year of virus isolation (so-called heterochronous sampling). Our results are, however, in agreement with the observation by Gilbert and Feschotte (2010) that long-term evolutionary rates of exogenous hepadnaviruses are

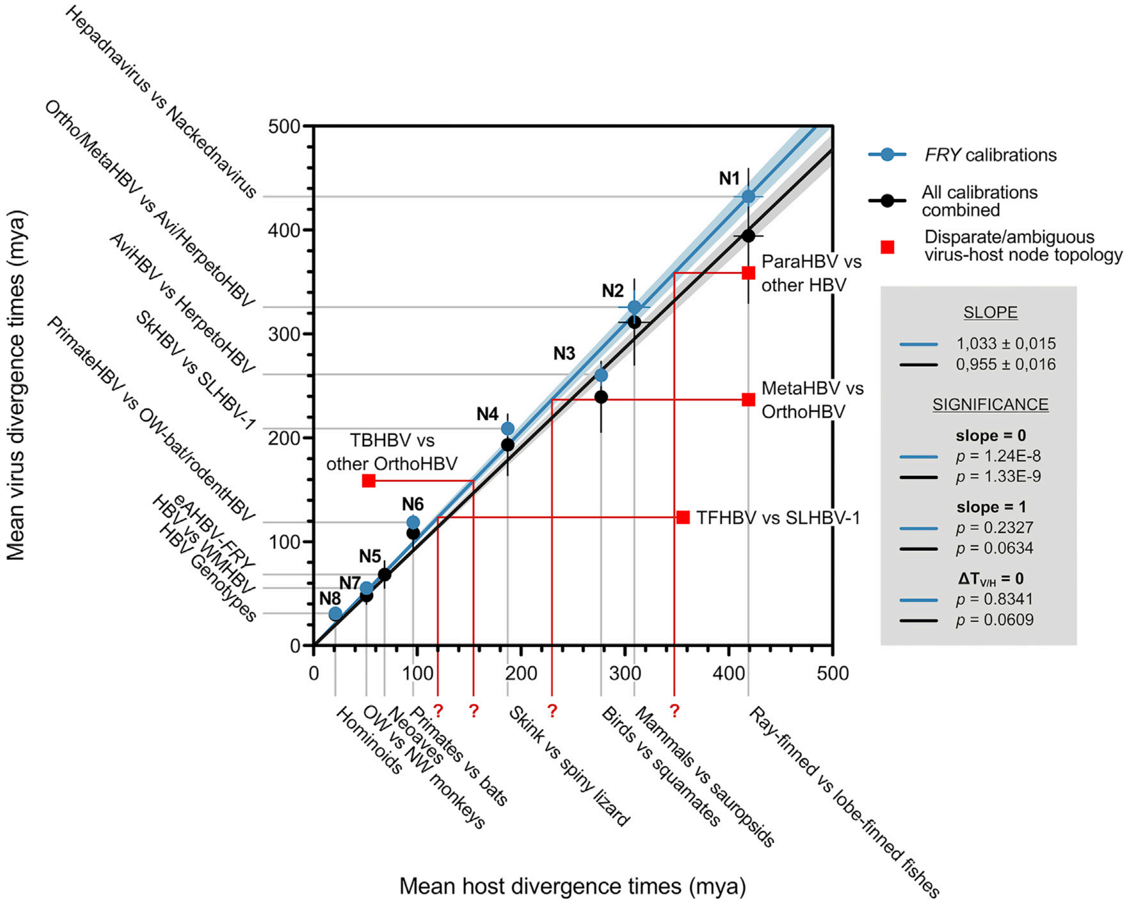

Figure 6. Correlation of Mean Divergence Times between Hepadnaviruses and Their Hosts

For the nodes in the hepadnaviral phylogeny representing putative virus-host cospeciation events (Figures 5 and S6A), the mean virus divergence times obtained with the calibrations based on eAHBV-FRY (blue) and the 11 independent calibrations based on the branching of exogenous hepadnaviruses in addition (black), were plotted against the mean host divergence times as retrieved from the literature (The Timetree of Life, 2016; Betancur et al., 2013; Bininda-Emonds et al., 2007; Hedges et al., 2015; Wang et al., 2013) (raw data in Table S2). Vertical and horizontal bars: SD. The nodes (N1–N8) are numbered as in Figures 5 and S6B. The linear regression of the eAHBV-FRY-calibrated nodes indicates a tight congruence between the related virus and host speciation times (blue line; 95% confidence interval: light blue background). Of note, the mean age estimate for the node of eAHBV-FRY resulting from the control calibrations (N5; 67.9 ± 13.6 mya SD) was consistent with the onset of the diversification of Neoaves (69–67 mya), implying that the long-term substitution rate of P proteins does not significantly differ between exogenous and endogenous hepadnaviruses. Both linear regressions had a significant deviation of the slope from 0, a non-significant deviation of the slope from 1, and the differences between the related virus and host divergence times ($\Delta T_{V/H}$) did not significantly differ from 0 (box with gray background). Red squares: the eAHBV-FRY-based node age estimates for the major viral nodes with disparate or ambiguous virus-host topology (Figures 5 and S6A) were plotted against the divergence times of the corresponding present-day hosts. The significant deviation of these nodes from the linear correlation of such nodes with congruent virus-host topology indicates a host switch to have occurred. The red lines and question marks indicate the expected age of the putative initial host reservoir, if these viruses also originated from a virus-host cospeciation event *before* they switched into a new host. For example parahepadnaviruses, i.e., WSHBV and CSKV, split off from all other hepadnaviruses 359 mya, i.e., at about the same time, when amphibians and amniotes diverged (352 mya according to http://timetree.org/). Likewise, TBHBV, so far the only known hepadnavirus from a South American bat (Drexler et al., 2013), separated from the other orthohepadnaviruses 158 mya, i.e., at about the same time, when placental and marsupial mammals diverged (159 mya according to http://timetree.org/). These observations might at least give a clue where to search for similar viruses.

at least 1,000-fold slower than expected from those former coalescent approaches representing short-term evolution. This inverse correlation between substitution rates and depth of time was confirmed in a study on intra- and inter-host evolution of human HBV in a family of chronic carriers over an ~100 year period of virus diversification (Lin et al., 2015). The discrepancy between fast short-term and slow long-term evolutionary rates has been widely recognized for hepadnaviruses by now (Godoy et al., 2013; Zehender et al., 2014; Littlejohn et al., 2016), and it turned out as a general rule probably applying to all major groups of viruses (Sharp and Simmonds, 2011; Patel et al., 2011; Feschotte and Gilbert, 2012). Importantly, Lin et al. (2015) found a significant variability of the mutation frequencies across different regions of the HBV genome with non-synonymous substitutions clustering at immune epitopes of structural genes. In our phylogenetic analyses we focused on the conserved parts of the viral replicase P and blanked out the highly variable sequence blocks that readily become saturated (Data S3). P is not a major target of the immune system, exerts its function in the cytoplasm, an evolutionary stable environment, and depends

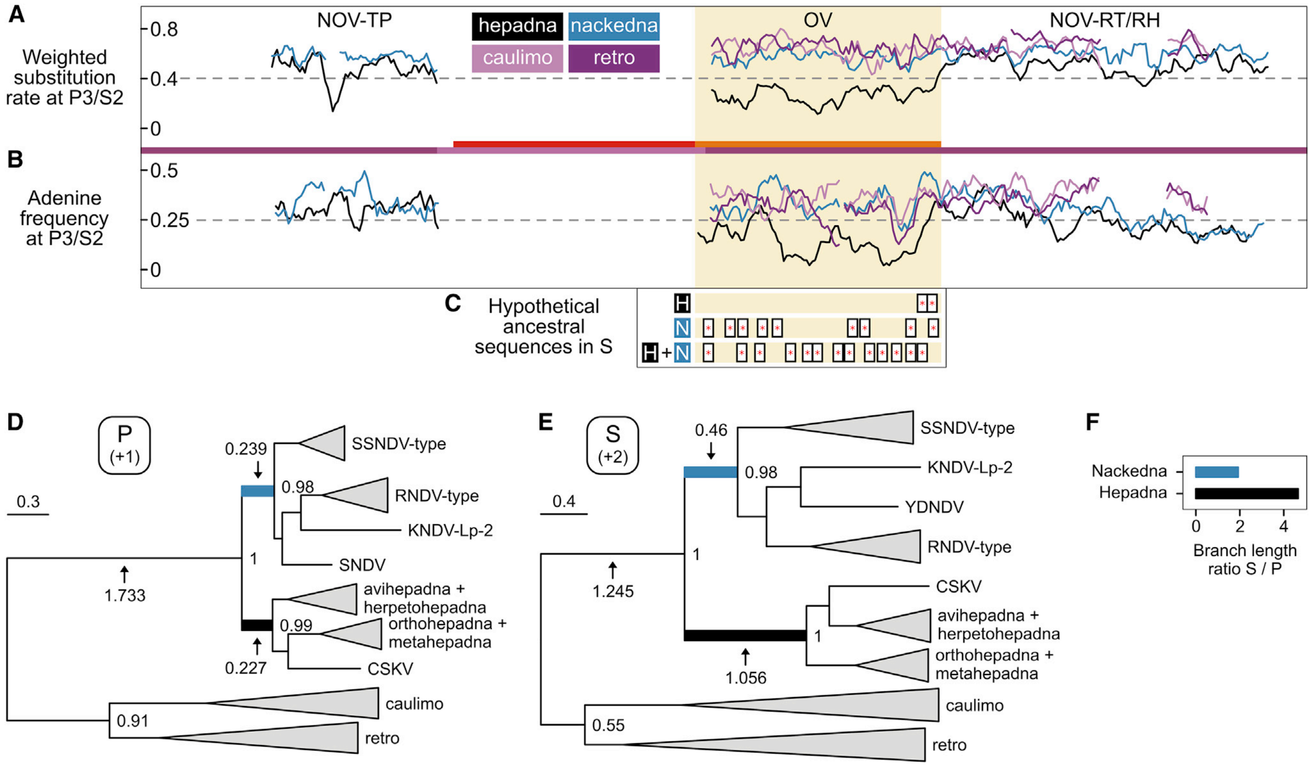

Figure 7. ***De Novo*** **Emergence of PreS/S in Hepadnaviruses**

(A) Weighted substitution rates at codon position 3 in the P frame, which equals codon position 2 in the S frame (P3/S2) for conserved regions in TP, RT, and RH. RT/S overlap region (OV) in hepadnaviruses highlighted by light-yellow background. NOV, non-overlapping regions.

(B) Adenine frequencies at P3/S2 positions.

(C) Hypothetical ancestral sequences for the overlap region reconstructed for the ancestors of hepadnaviruses (H), nackednaviruses (N), and hepadna- and nackednaviruses (H + N). Predicted stop codons in the S frame are highlighted.

(D) Phylogeny for the RT/S overlap region translated in the P frame. Scale bar, substitutions per site. Branches representing the relevant time window after the split between nackedna- and hepadnaviruses and before the first intragroup speciation events are colored and their lengths are indicated.

(E) Phylogeny for the RT/S overlap region translated in the S frame.

(F) Ratio of analogous branches in the P and S frame-based phylogenies for the RT/S overlap, estimating the relative evolutionary change in the S frame between viral lineages.

on interaction with slowly evolving cellular housekeeping factors such as chaperones, e.g., Hsp90, Hsp70, and Hsp40, to form a functional replication complex (Nassal, 2008; Nguyen et al., 2008). We would therefore expect the fittest P sequences to evolve over deep timescales mainly in response to the slow changes of the cytoplasmic environment. Consequently, it is not surprising for us to find evolutionary rates for the conserved parts of P from exogenous hepadnaviruses equaling those after endogenization, thus providing a proof of principle for the feasibility to use selected sequences of endogenous viral elements for time calibrations. Interestingly, our observations comply with the "covarion model," according to which the requirement to cooperate with host proteins imposes functional constraints on the number of viral protein residues that can accept substitutions at a given point in time, thus decelerating viral protein divergence (Koonin and Gorbalenya, 1989). In this respect, our results invite critical rethinking regarding the frequent use of molecular clocks reflecting short-term evolution to infer time estimates for deep viral phylogenies, and we hope that our taxon sampling provides a suitable test system to develop and evaluate additional methods for dating the long-term evolutionary history of viruses, as previously suggested (Sharp and Simmonds, 2011).

To explain the slowdown of the evolutionary rates over time, Lin et al. (2015) proposed a model of continuous switching of the viral mutant spectrum between colonization and adaptation. According to this, "colonizers" are optimally replicating viruses that are in advantage early after transmission into an immunologically naive host, while "adaptors" diversify under pressure of the host immune system during the late inflammatory phase of chronic infection at the cost of replicative fitness. The fast short-term evolutionary rates are hence attributed to the intra-host divergence of the "adaptors," whereas the back-selection toward "colonizers" succeeding each transmission event is thought to be responsible for the slow long-term evolution. Episomally persisting, circular viral DNA is supposed to represent a permanent reservoir of "colonizer" genomes in this model. Noteworthy, natural infections with hepadnaviruses are typically acquired early in the lifetime of the host and somatic integration of non-canonical, linearized viral DNA species into the genomes of hepatocytes can be detected within hours after transmission (Chauhan et al., 2017; Mason et al., 2016; Sung et al., 2012).

Such integrated viral genome copies will evolve at the substitution rate of the host cell genome, and if they had the potential to constantly replenish the quasispecies of circulating virus during lifelong chronic infection with particles resembling the initial inoculum, the viral generation times could approximate host generation times. This in turn might contribute to the synchronous long-term virus-host evolution, as we observe it for P.

We speculate that the co-evolution of tetrapod hepadnaviruses with their hosts over geologic eras could be a direct consequence of their specialization to a single organ, the liver, while the fish viruses reside in a broad spectrum of tissues and organs, which might favor frequent host switches. The liver tropism of tetrapod hepadnaviruses in turn is primarily determined by the interaction of PreS with receptor molecules on the hepatocyte surface during virus entry (Glebe and Urban, 2007). All lines of evidence argue for the *de novo* emergence of PreS/S in the hepadnaviral lineage after 432 mya and before 360 mya (Figure 4). The evolution of the surface protein gene must have involved two distinct processes: first, the insertion of additional nucleic acid between the TP and RT domains of P that led to the spacer and the PreS region in the two respective reading frames; second, the generation of the S part by overprinting of the pre-existing RT-coding sequence in the alternative reading frame. The accompanying increase in genome size might then have triggered a symmetry switch from small T=3 to large T=4 particles as predominant capsid type. In this regard, it is worth remembering that HBV-infected cells do not only secrete enveloped virions, but also naked capsids (Ni et al., 2010; Bardens et al., 2011). This might be a mere vestigial feature retained from their distant past as non-enveloped viruses. However, an intriguing possibility is that these naked capsids may still play an important role in establishing or maintaining an infection with HBV.

To our knowledge, it is the first reported case of the *de novo* emergence of a completely overlapping gene encoding for essential structural proteins by such a mechanism. Typical examples for the evolution of gene overlap by overprinting are small accessory genes coding for regulatory factors (Rancurel et al., 2009), e.g., those found in the genomes of deltaretroviruses (Pavesi et al., 2013). Becoming enveloped, on the other hand, frequently involves incorporation of yet fully functional genetic modules from other viruses through heterologous recombination, as was described for different invertebrate retrotransposons (family *Metaviridae*), which independently captured envelope protein genes from phlebo-, herpes-, or baculoviruses, respectively (Malik et al., 2000; Kim et al., 2004). The twinned virus families presented in our study therefore constitute an unprecedented example for a fundamental transition in viral lifestyle.

STAR★METHODS

Detailed methods are provided in the online version of this paper and include the following:

- KEY RESOURCES TABLE
- CONTACT FOR REAGENT AND RESOURCE SHARING
- EXPERIMENTAL MODEL AND SUBJECT DETAILS
  - Cell Line HuH-7
- METHOD DETAILS
  - Search and Assembly of Viral Genomes
  - *In Vitro* Virological Assays
  - Ultrastructure of Nackednavirus Capsids
  - Sequence Alignments
  - Phylogenetic Reconstructions
  - Virus-Host Cophylogeny Testing
  - Identifying and Reconstructing eAHBV-FRY
  - Time-Calibration of the Viral Phylogeny
  - Bayesian Phylogenetic Model Optimization
  - S Protein Evolution
- DATA AVAILABILITY

SUPPLEMENTAL INFORMATION

Supplemental Information includes seven figures, three tables, and four data files and can be found with this article online at http://dx.doi.org/10.1016/j.chom.2017.07.019.

AUTHOR CONTRIBUTIONS

R.B. and S.S. designed the study. S.S. and C.L. discovered viruses in sequence databases. J.H., C.L., and S.S. assembled viral genomes from the SRA. C.L. and S.S. constructed multiple sequence alignments. C.L. and A.S. performed all other bioinformatics under advice from L.K. W.S. and S.S. physically amplified ACNDV sequences from cDNA of *O. ventralis*. J. Börold performed *in vitro* replication assays. J. Beck designed and performed priming assays. J. Börold and S.S. expressed and purified nackednaviral capsids. S.M., S.S., and J.A.G.B. performed electron microscopy analyses. S.M. generated 3D image reconstructions. S.S., C.L., and R.B. wrote the manuscript. All authors discussed data and commented on the manuscript.

ACKNOWLEDGMENTS

We are grateful to all colleagues in the scientific community who make their sequencing data publicly accessible. We acknowledge the NCBI for providing an elaborate platform to exchange sequencing data. We thank the Center for Information Services and High Performance Computing (ZIH) at TU Dresden for generous allocations of computer time, E. Santos and L. Baldo for preparing cDNA of *O. ventralis*, EMBL IT services and W. Hagen for technical support, and S. Urban and M. Nassal for critical discussion. Work of R.B. was supported in part by a grant from the Deutsche Forschungsgemeinschaft (SFB/Transregio 179, TP9); the work of J. Beck was supported in part by the Deutsche Forschungsgemeinschaft grant Na154/9-4 to M. Nassal.

Received: March 22, 2017
Revised: July 10, 2017
Accepted: July 29, 2017
Published: August 31, 2017

SUPPORTING CITATIONS

The following references appear in the Supplemental Information: Castoe et al. (2009); Kyriazi et al. (2013); Pyron and Burbrink (2012); Rambaut et al. (2016b); van Tuinen and Torres (2015); Zhang et al. (2014); Zheng and Wiens (2016).

REFERENCES

Abascal, F., Zardoya, R., and Posada, D. (2005). ProtTest: selection of best-fit models of protein evolution. Bioinformatics *21*, 2104–2105.

Altschul, S.F., Gish, W., Miller, W., Myers, E.W., and Lipman, D.J. (1990). Basic local alignment search tool. J. Mol. Biol. *215*, 403–410.

Andrews, S. (2010). FastQC. A quality control tool for high throughput sequence data. http://www.bioinformatics.babraham.ac.uk/projects/fastqc.

Baele, G., Lemey, P., Bedford, T., Rambaut, A., Suchard, M.A., and Alekseyenko, A.V. (2012). Improving the accuracy of demographic and molecular clock model comparison while accommodating phylogenetic uncertainty. Mol. Biol. Evol. *29*, 2157–2167.

Baldo, L., Santos, M.E., and Salzburger, W. (2011). Comparative transcriptomics of Eastern African cichlid fishes shows signs of positive selection and a large contribution of untranslated regions to genetic diversity. Genome Biol. Evol. *3*, 443–455.

Bankevich, A., Nurk, S., Antipov, D., Gurevich, A.A., Dvorkin, M., Kulikov, A.S., Lesin, V.M., Nikolenko, S.I., Pham, S., Prjibelski, A.D., et al. (2012). SPAdes: a new genome assembly algorithm and its applications to single-cell sequencing. J. Comput. Biol. *19*, 455–477.

Bardens, A., Döring, T., Stieler, J., and Prange, R. (2011). Alix regulates egress of hepatitis B virus naked capsid particles in an ESCRT-independent manner. Cell Microbiol. *13*, 602–619.

Bartenschlager, R., and Schaller, H. (1988). The amino-terminal domain of the hepadnaviral P-gene encodes the terminal protein (genome-linked protein) believed to prime reverse transcription. EMBO J. *7*, 4185–4192.

Beck, J., and Nassal, M. (2007). Hepatitis B virus replication. World J. Gastroenterol. *13*, 48–64.

Begun, D.R. (2003). Planet of the apes. Sci. Am. *289*, 74–83.

Betancur, R., Broughton, R.E., Wiley, E.O., Carpenter, K., Lopez, J.A., Li, C., Holcroft, N.I., Arcila, D., Sanciangco, M., Cureton Ii, J.C., et al. (2013). The tree of life and a new classification of bony fishes. PLoS Curr. *5*, http://dx.doi.org/10.1371/currents.tol.53ba26640df0ccaee75bb165c8c26288.

Bininda-Emonds, O.R., Cardillo, M., Jones, K.E., MacPhee, R.D., Beck, R.M., Grenyer, R., Price, S.A., Vos, R.A., Gittleman, J.L., and Purvis, A. (2007). The delayed rise of present-day mammals. Nature *446*, 507–512.

Castoe, T.A., Gu, W., de Koning, A.P., Daza, J.M., Jiang, Z.J., Parkinson, C.L., and Pollock, D.D. (2009). Dynamic nucleotide mutation gradients and control region usage in squamate reptile mitochondrial genomes. Cytogenet. Genome Res. *127*, 112–127.

Chauhan, R., Churchill, N.D., Mulrooney-Cousins, P.M., and Michalak, T.I. (2017). Initial sites of hepadnavirus integration into host genome in human hepatocytes and in the woodchuck model of hepatitis B-associated hepatocellular carcinoma. Oncogenesis *6*, e317.

Chirico, N., Vianelli, A., and Belshaw, R. (2010). Why genes overlap in viruses. Proc. Biol. Sci. *277*, 3809–3817.

Claramunt, S., and Cracraft, J. (2015). A new time tree reveals Earth history's imprint on the evolution of modern birds. Sci. Adv. *1*, e1501005.

Conow, C., Fielder, D., Ovadia, Y., and Libeskind-Hadas, R. (2010). Jane: a new tool for the cophylogeny reconstruction problem. Algorithms. Mol. Biol. *5*, 16.

Crowther, R.A., Kiselev, N.A., Bottcher, B., Berriman, J.A., Borisova, G.P., Ose, V., and Pumpens, P. (1994). Three-dimensional structure of hepatitis B virus core particles determined by electron cryomicroscopy. Cell *77*, 943–950.

Dill, J.A., Camus, A.C., Leary, J.H., Di, G.F., Holmes, E.C., and Ng, T.F. (2016). Distinct viral lineages from fish and amphibians reveal the complex evolutionary history of hepadnaviruses. J. Virol. *90*, 7920–7933.

Drexler, J.F., Geipel, A., König, A., Corman, V.M., van Riel, D., Leijten, L.M., Bremer, C.M., Rasche, A., Cottontail, V.M., Maganga, G.D., et al. (2013). Bats carry pathogenic hepadnaviruses antigenically related to hepatitis B virus and capable of infecting human hepatocytes. Proc. Natl. Acad. Sci. USA *110*, 16151–16156.

Drozdetskiy, A., Cole, C., Procter, J., and Barton, G.J. (2015). JPred4: a protein secondary structure prediction server. Nucleic Acids Res. *43*, W389–W394.

Drummond, A.J., Ho, S.Y., Phillips, M.J., and Rambaut, A. (2006). Relaxed phylogenetics and dating with confidence. PLoS Biol. *4*, e88.

Drummond, A.J., Suchard, M.A., Xie, D., and Rambaut, A. (2012). Bayesian phylogenetics with BEAUti and the BEAST 1.7. Mol. Biol. Evol. *29*, 1969–1973.

Edgar, R.C. (2004). MUSCLE: multiple sequence alignment with high accuracy and high throughput. Nucleic Acids Res. *32*, 1792–1797.

Feschotte, C., and Gilbert, C. (2012). Endogenous viruses: insights into viral evolution and impact on host biology. Nat. Rev. Genet. *13*, 283–296.

Geoghegan, J.L., Duchene, S., and Holmes, E.C. (2017). Comparative analysis estimates the relative frequencies of co-divergence and cross-species transmission within viral families. PLoS Pathog. *13*, e1006215.

Gilbert, C., and Feschotte, C. (2010). Genomic fossils calibrate the long-term evolution of hepadnaviruses. PLoS Biol. *8*, http://dx.doi.org/10.1371/journal.pbio.1000495.

Gilbert, C., Meik, J.M., Dashevsky, D., Card, D.C., Castoe, T.A., and Schaack, S. (2014). Endogenous hepadnaviruses, bornaviruses and circoviruses in snakes. Proc. Biol. Sci. *281*, 20141122.

Glebe, D., and Urban, S. (2007). Viral and cellular determinants involved in hepadnaviral entry. World J. Gastroenterol. *13*, 22–38.

Godoy, B.A., Alvarado-Mora, M.V., Gomes-Gouvea, M.S., Pinho, J.R., and Fagundes, N., Jr. (2013). Origin of HBV and its arrival in the Americas—the importance of natural selection on time estimates. Antivir. Ther. *18*, 505–512.

Gouy, M., Guindon, S., and Gascuel, O. (2010). SeaView version 4: a multiplatform graphical user interface for sequence alignment and phylogenetic tree building. Mol. Biol. Evol. *27*, 221–224.

Guindon, S., Dufayard, J.F., Lefort, V., Anisimova, M., Hordijk, W., and Gascuel, O. (2010). New algorithms and methods to estimate maximum-likelihood phylogenies: assessing the performance of PhyML 3.0. Syst. Biol. *59*, 307–321.

Hahn, C.M., Iwanowicz, L.R., Cornman, R.S., Conway, C.M., Winton, J.R., and Blazer, V.S. (2015). Characterization of a novel hepadnavirus in the white sucker (*Catostomus commersonii*) from the Great Lakes Region of the United States. J. Virol. *89*, 11801–11811.

Hedges, S.B., Marin, J., Suleski, M., Paymer, M., and Kumar, S. (2015). Tree of life reveals clock-like speciation and diversification. Mol. Biol. Evol. *32*, 835–845.

Hong, M., Sandalova, E., Low, D., Gehring, A.J., Fieni, S., Amadei, B., Urbani, S., Chong, Y.S., Guccione, E., and Bertoletti, A. (2015). Trained immunity in newborn infants of HBV-infected mothers. Nat. Commun. *6*, 6588.

Jarvis, E.D., Mirarab, S., Aberer, A.J., Li, B., Houde, P., Li, C., Ho, S.Y., Faircloth, B.C., Nabholz, B., Howard, J.T., et al. (2014). Whole-genome analyses resolve early branches in the tree of life of modern birds. Science *346*, 1320–1331.

Jones, D.T. (1999). Protein secondary structure prediction based on position-specific scoring matrices. J. Mol. Biol. *292*, 195–202.

Keese, P.K., and Gibbs, A. (1992). Origins of genes: "big bang" or continuous creation? Proc. Natl. Acad. Sci. USA *89*, 9489–9493.

Kim, F.J., Battini, J.L., Manel, N., and Sitbon, M. (2004). Emergence of vertebrate retroviruses and envelope capture. Virology *318*, 183–191.

Koonin, E.V., and Gorbalenya, A.E. (1989). Evolution of RNA genomes: does the high mutation rate necessitate high rate of evolution of viral proteins? J. Mol. Evol. *28*, 524–527.

Kyriazi, P., Kornilios, P., Nagy, Z.T., Poulakakis, N., Kumlutaş, Y., Ilgaz, Ç., Avcı, A., Göçmen, B., and Lymberakis, P. (2013). Comparative phylogeography reveals distinct colonization patterns of Cretan snakes. J. Biogeogr. *40*, 1143–1155.

Legendre, P., Desdevises, Y., and Bazin, E. (2002). A statistical test for host-parasite coevolution. Syst. Biol. *51*, 217–234.

Lin, Y.Y., Liu, C., Chien, W.H., Wu, L.L., Tao, Y., Wu, D., Lu, X., Hsieh, C.H., Chen, P.J., Wang, H.Y., et al. (2015). New insights into the evolutionary rate of hepatitis B virus at different biological scales. J. Virol. *89*, 3512–3522.

Littlejohn, M., Locarnini, S., and Yuen, L. (2016). Origins and evolution of hepatitis B virus and hepatitis D virus. Cold Spring Harb. Perspect. Med. *6*, a021360.

Liu, W., Pan, S., Yang, H., Bai, W., Shen, Z., Liu, J., and Xie, Y. (2012). The first full-length endogenous hepadnaviruses: identification and analysis. J. Virol. *86*, 9510–9513.

Malik, H.S., Henikoff, S., and Eickbush, T.H. (2000). Poised for contagion: evolutionary origins of the infectious abilities of invertebrate retroviruses. Genome Res. *10*, 1307–1318.

Martin, M. (2011). Cutadapt removes adapter sequences from high-throughput sequencing reads. EMBnet. J. *17*, 10–12.

Mason, W.S., Gill, U.S., Litwin, S., Zhou, Y., Peri, S., Pop, O., Hong, M.L., Naik, S., Quaglia, A., Bertoletti, A., and Kennedy, P.T. (2016). HBV DNA integration and clonal hepatocyte expansion in chronic hepatitis B patients considered immune tolerant. Gastroenterology *151*, 986–998.

Mindell, J.A., and Grigorieff, N. (2003). Accurate determination of local defocus and specimen tilt in electron microscopy. J. Struct. Biol. *142*, 334–347.

Mizokami, M., and Orito, E. (1999). Molecular evolution of hepatitis viruses. Intervirology *42*, 159–165.

Nassal, M. (2008). Hepatitis B viruses: reverse transcription a different way. Virus Res. *134*, 235–249.

Nguyen, D.H., Ludgate, L., and Hu, J. (2008). Hepatitis B virus-cell interactions and pathogenesis. J. Cell Physiol. *216*, 289–294.

Ni, Y., Sonnabend, J., Seitz, S., and Urban, S. (2010). The pre-s2 domain of the hepatitis B virus is dispensable for infectivity but serves a spacer function for L-protein-connected virus assembly. J. Virol. *84*, 3879–3888.

Orito, E., Mizokami, M., Ina, Y., Moriyama, E.N., Kameshima, N., Yamamoto, M., and Gojobori, T. (1989). Host-independent evolution and a genetic classification of the hepadnavirus family based on nucleotide sequences. Proc. Natl. Acad. Sci. USA *86*, 7059–7062.

Patel, M.R., Emerman, M., and Malik, H.S. (2011). Paleovirology—ghosts and gifts of viruses past. Curr. Opin. Virol. *1*, 304–309.

Pavesi, A. (2015). Different patterns of codon usage in the overlapping polymerase and surface genes of hepatitis B virus suggest a de novo origin by modular evolution. J. Gen. Virol. *96*, 3577–3586.

Pavesi, A., Magiorkinis, G., and Karlin, D.G. (2013). Viral proteins originated de novo by overprinting can be identified by codon usage: application to the "gene nursery" of Deltaretroviruses. PLoS Comput. Biol. *9*, e1003162.

Prum, R.O., Berv, J.S., Dornburg, A., Field, D.J., Townsend, J.P., Lemmon, E.M., and Lemmon, A.R. (2015). A comprehensive phylogeny of birds (Aves) using targeted next-generation DNA sequencing. Nature *526*, 569–573.

Pyron, R.A., and Burbrink, F.T. (2012). Extinction, ecological opportunity, and the origins of global snake diversity. Evolution *66*, 163–178.

Rambaut, A. (2016). FigTree. http://tree.bio.ed.ac.uk/software/figtree.

Rambaut, A., Suchard, M.A., and Drummond, A.J. (2016a). Tracer. http://tree.bio.ed.ac.uk/software/tracer.

Rambaut, A., Lam, T.T., Max, C.L., and Pybus, O.G. (2016b). Exploring the temporal structure of heterochronous sequences using TempEst (formerly Path-O-Gen). Virus Evol. *2*, vew007.

Rancurel, C., Khosravi, M., Dunker, A.K., Romero, P.R., and Karlin, D. (2009). Overlapping genes produce proteins with unusual sequence properties and offer insight into de novo protein creation. J. Virol. *83*, 10719–10736.

Ranwez, V., Harispe, S., Delsuc, F., and Douzery, E.J. (2011). MACSE: multiple alignment of coding SEquences accounting for frameshifts and stop codons. PLoS One *6*, e22594.

Remmert, M., Biegert, A., Hauser, A., and Soding, J. (2012). HHblits: lightning-fast iterative protein sequence searching by HMM-HMM alignment. Nat. Methods *9*, 173–175.

Schaefer, S. (2007). Hepatitis B virus taxonomy and hepatitis B virus genotypes. World J. Gastroenterol. *13*, 14–21.

Scheres, S.H. (2012). RELION: implementation of a Bayesian approach to cryo-EM structure determination. J. Struct. Biol. *180*, 519–530.

Seeger, C., and Mason, W.S. (2000). Hepatitis B virus biology. Microbiol. Mol. Biol. Rev. *64*, 51–68.

Sharp, P.M., and Simmonds, P. (2011). Evaluating the evidence for virus/host co-evolution. Curr. Opin. Virol. *1*, 436–441.

Simpson, J.T., Wong, K., Jackman, S.D., Schein, J.E., Jones, S.J., and Birol, I. (2009). ABySS: a parallel assembler for short read sequence data. Genome Res. *19*, 1117–1123.

Springer, M.S., Meredith, R.W., Gatesy, J., Emerling, C.A., Park, J., Rabosky, D.L., Stadler, T., Steiner, C., Ryder, O.A., Janecka, J.E., et al. (2012). Macroevolutionary dynamics and historical biogeography of primate diversification inferred from a species supermatrix. PLoS One *7*, e49521.

Stevens, N.J., Seiffert, E.R., O'Connor, P.M., Roberts, E.M., Schmitz, M.D., Krause, C., Gorscak, E., Ngasala, S., Hieronymus, T.L., and Temu, J. (2013). Palaeontological evidence for an Oligocene divergence between Old World monkeys and apes. Nature *497*, 611–614.

Suh, A., Brosius, J., Schmitz, J., and Kriegs, J.O. (2013). The genome of a Mesozoic paleovirus reveals the evolution of hepatitis B viruses. Nat. Commun. *4*, 1791.

Suh, A., Weber, C.C., Kehlmaier, C., Braun, E.L., Green, R.E., Fritz, U., Ray, D.A., and Ellegren, H. (2014). Early mesozoic coexistence of amniotes and Hepadnaviridae. PLoS Genet. *10*, e1004559.

Sung, W.K., Zheng, H., Li, S., Chen, R., Liu, X., Li, Y., Lee, N.P., Lee, W.H., Ariyaratne, P.N., Tennakoon, C., et al. (2012). Genome-wide survey of recurrent HBV integration in hepatocellular carcinoma. Nat. Genet. *44*, 765–769.

Tamura, K., Stecher, G., Peterson, D., Filipski, A., and Kumar, S. (2013). MEGA6: molecular evolutionary genetics analysis version 6.0. Mol. Biol. Evol. *30*, 2725–2729.

Tang, G., Peng, L., Baldwin, P.R., Mann, D.S., Jiang, W., Rees, I., and Ludtke, S.J. (2007). EMAN2: an extensible image processing suite for electron microscopy. J. Struct. Biol. *157*, 38–46.

The Timetree of Life. (2016); http://www.timetree.org/.

van Heel, M., Harauz, G., Orlova, E.V., Schmidt, R., and Schatz, M. (1996). A new generation of the IMAGIC image processing system. J. Struct. Biol. *116*, 17–24.

van Hemert, F.J., van de Klundert, M.A., Lukashov, V.V., Kootstra, N.A., Berkhout, B., and Zaaijer, H.L. (2011). Protein X of hepatitis B virus: origin and structure similarity with the central domain of DNA glycosylase. PLoS One *6*, e23392.

van Tuinen, M., and Torres, C.R. (2015). Potential for bias and low precision in molecular divergence time estimation of the Canopy of Life: an example from aquatic bird families. Front. Genet. *6*, 203.

Vogel, M., Vorreiter, J., and Nassal, M. (2005). Quaternary structure is critical for protein display on capsid-like particles (CLPs): efficient generation of hepatitis B virus CLPs presenting monomeric but not dimeric and tetrameric fluorescent proteins. Proteins *58*, 478–488.

Wang, Z., Pascual-Anaya, J., Zadissa, A., Li, W., Niimura, Y., Huang, Z., Li, C., White, S., Xiong, Z., Fang, D., et al. (2013). The draft genomes of soft-shell turtle and green sea turtle yield insights into the development and evolution of the turtle-specific body plan. Nat. Genet. *45*, 701–706.

Waterhouse, A.M., Procter, J.B., Martin, D.M., Clamp, M., and Barton, G.J. (2009). Jalview version 2—a multiple sequence alignment editor and analysis workbench. Bioinformatics *25*, 1189–1191.

Weber, M., Bronsema, V., Bartos, H., Bosserhoff, A., Bartenschlager, R., and Schaller, H. (1994). Hepadnavirus P protein utilizes a tyrosine residue in the TP domain to prime reverse transcription. J. Virol. *68*, 2994–2999.

WHO. (2017). Hepatitis B. http://www.who.int/mediacentre/factsheets/fs204/en/.

Wynne, S.A., Crowther, R.A., and Leslie, A.G. (1999). The crystal structure of the human hepatitis B virus capsid. Mol. Cell *3*, 771–780.

Yang, J.D., and Roberts, L.R. (2010). Hepatocellular carcinoma: a global view. Nat. Rev. Gastroenterol. Hepatol. *7*, 448–458.

Yu, X., Jin, L., Jih, J., Shih, C., and Zhou, Z.H. (2013). 3.5A cryoEM structure of hepatitis B virus core assembled from full-length core protein. PLoS One *8*, e69729.

Zaaijer, H.L., van Hemert, F.J., Koppelman, M.H., and Lukashov, V.V. (2007). Independent evolution of overlapping polymerase and surface protein genes of hepatitis B virus. J. Gen. Virol. *88*, 2137–2143.

Zehender, G., Ebranati, E., Gabanelli, E., Sorrentino, C., Lo, P.A., Tanzi, E., Ciccozzi, M., and Galli, M. (2014). Enigmatic origin of hepatitis B virus: an ancient travelling companion or a recent encounter? World J. Gastroenterol. *20*, 7622–7634.

Zhang, G., Li, C., Li, Q., Li, B., Larkin, D.M., Lee, C., Storz, J.F., Antunes, A., Greenwold, M.J., Meredith, R.W., et al. (2014). Comparative genomics reveals insights into avian genome evolution and adaptation. Science *346*, 1311–1320.

Zheng, Y., and Wiens, J.J. (2016). Combining phylogenomic and supermatrix approaches, and a time-calibrated phylogeny for squamate reptiles (lizards and snakes) based on 52 genes and 4162 species. Mol. Phylogenet. Evol. *94*, 537–547.

STAR★METHODS

KEY RESOURCES TABLE

REAGENT or RESOURCE	SOURCE	IDENTIFIER
Chemicals, Peptides, and Recombinant Proteins		
dGTP [α-^{32}P] 3000Ci/mmol 10mCi/ml	Perkin Elmer	Cat#BLU514H250UC
Methionine L-[^{35}S] Premium Stabilized T	Perkin Elmer	Cat#NEG009A500UC
Critical Commercial Assays		
TNT Quick Coupled Transcription/ Translation System	Promega	Cat#L1170
Deposited Data		
Viral genome sequences	This paper	Data S1 (gb format)
P protein alignment	This paper	Data S2 (fasta format)
Full-length ACNDV capsid structure	This paper	EMDB: EMD-3822
Truncated ACNDV capsid structure	This paper	EMDB: EMD-3823
Experimental Models: Cell Lines		
HuH-7 cells	Japanese Collection of Research Bioresources (JCRB)	Cat#JCRB0403
Recombinant DNA		
Construct for eukaryotic RNDV genome expression	This paper	pcDNA3.1(+)-RNDV
Construct for wt RNDV P protein in vitro transcription/translation	This paper	pT7/AMV-RNDVpol
Construct for YMHD mutant RNDV P in vitro transcription/translation	This paper	pT7/AMV-RNDVpol-YMHD
Construct for bacterial expression of full-length ACNDV capsid protein	This paper	pET28a2-ACNDVc1-174
Construct for bacterial expression of truncated ACNDV capsid protein	This paper	pET28a2-ACNDVc1-146
Software and Algorithms		
Blast	Altschul et al., 1990	https://blast.ncbi.nlm.nih.gov; RRID: SCR_004870
FastQC	Andrews, 2010	https://www.bioinformatics.babraham.ac.uk/projects/fastqc/; RRID: SCR_014583
Cutadapt	Martin, 2011	http://cutadapt.readthedocs.io/en/stable/guide.html; RRID: SCR_011841
ABySS	Simpson et al., 2009	http://www.bcgsc.ca/platform/bioinfo/software/abyss; RRID: SCR_010709
SPAdes	Bankevich et al., 2012	http://bioinf.spbau.ru/spades; RRID: SCR_000131
SeaView	Gouy et al., 2010	http://doua.prabi.fr/software/seaview; RRID: SCR_015059
Muscle	Edgar, 2004	http://www.drive5.com/muscle/
HHsuite	Remmert et al., 2012	http://www.soeding.genzentrum.lmu.de/software-and-servers-2/
Jalview	Waterhouse et al., 2009	http://www.jalview.org/; RRID: SCR_006459
ProtTest 2	Abascal et al., 2005	http://darwin.uvigo.es/software/prottest2_server.html
BEAST	Drummond et al., 2012	http://beast.bio.ed.ac.uk/; RRID: SCR_010228
Tracer	Rambaut, 2016	http://tree.bio.ed.ac.uk/software/tracer/

(*Continued on next page*)

Continued		
REAGENT or RESOURCE	SOURCE	IDENTIFIER
ParaFit	Legendre et al., 2002	https://cran.r-project.org/web/packages/ape/index.html
Jane4	Conow et al., 2010	https://www.cs.hmc.edu/~hadas/jane/
MACSE	Ranwez et al., 2011	http://mbb.univ-montp2.fr/MBB/subsection/softExec.php?soft=macse
PhyML 3	Guindon et al., 2010	http://www.atgc-montpellier.fr/phyml/; RRID: SCR_014629
MEGA 6	Tamura et al., 2013	http://www.megasoftware.net/; RRID: SCR_000667
FigTree	Rambaut, 2016	http://tree.bio.ed.ac.uk/software/figtree/; RRID: SCR_008515
EPU	FEI	https://www.fei.com/software/epu/
CTFFIND3	Mindell and Grigorieff, 2003	http://grigoriefflab.janelia.org/ctf
EMAN2	Tang et al., 2007	http://blake.bcm.edu/emanwiki/EMAN2
RELION 1.2	Scheres, 2012	http://www2.mrc-lmb.cam.ac.uk/relion/index.php/Main_Page
IMAGIC	van Heel et al., 1996	https://www.imagescience.de/imagic.html; RRID: SCR_014447

CONTACT FOR REAGENT AND RESOURCE SHARING

Further information and requests for resources, reagents, and data should be directed to and will be fulfilled by the Lead Contact, Stefan Seitz (stefan.seitz@med.uni-heidelberg.de).

EXPERIMENTAL MODEL AND SUBJECT DETAILS

Cell Line HuH-7

HuH-7 is a human cell line derived from a hepatocellular carcinoma. Sex: male. Culture conditions: 37°C, 5% CO_2 atmosphere, in Dulbecco's modified Eagle medium supplemented with 10% fetal calf serum, 2 mM L-glutamine, 100 U of penicillin/ml, and 100 µg of streptomycin/ml (Ni et al., 2010). Cells have not been authenticated.

METHOD DETAILS

Search and Assembly of Viral Genomes

We used tblastn (Altschul et al., 1990) to screen the Whole-genome Shotgun Assembly (WGS), Transcriptome Shotgun Assembly (TSA), and Sequence Read Archives (SRA) accessible at NCBI for the presence of unknown HBV-related sequences. The screened data included more than 25,000 individual SRA experiments from bony fish sequencing projects. Initially, we used the TP protein sequence of DHBV as single query. To increase sensitivity of the search, we later extended the query list by adding the TP sequences of several of the discovered hepadna- and nackednaviruses. A Blast hit was considered for downstream analysis (i) if it had a very good E-value of 10^{-4}, or (ii) if it had a moderate E-value of 10 and the same sequence read was found by at least two queries, or (iii) if at least two sequencing reads gave hits of 50% or better sequence identity. Moreover, all potential hits were verified manually by inspection of the Blast outputs. Raw sequencing reads of verified hits were downloaded from NCBI/SRA. FastQC (Andrews, 2010) was used for quality control and Cutadapt (Martin, 2011) for trimming adapter sequences and low-quality bases. For *de novo* assembly of a viral genome we used ABySS (Simpson et al., 2009) and SPAdes (Bankevich et al., 2012) with different kmer values ranging from 12 to 96 and chose the longest viral contig. Both ends of the linear assembly were completed manually to account for circularity of the viral genomes. For cases of very low read coverage resulting in fragmental genomes, we manually joined the fragments using closely related complete genomes as reference.

In Vitro Virological Assays

The whole genome sequence of RNDV was custom synthesized and inserted into the eukaryotic expression vector pcDNA3.1(+). Analogous HBV constructs (*wild-type* and an envelope protein deficient mutant) were used as control. HuH-7 cells on 10-cm diameter dishes were transfected with 10µg of plasmid DNA using the TransIT-LT1 reagent. To remove cell-associated plasmid DNA, cells were washed 3 times with PBS on day one and day two after transfection. In addition, cells were treated with DNase at day one. Culture supernatants were collected at day 10 after transfection. To separate enveloped and non-enveloped viral particles,

the supernatants were subjected to CsCl density gradient ultracentrifugation, followed by DNA-dot-blot analysis of gradient fractions as described previously for HBV (Ni et al., 2010).

RNDV P priming assays were performed as described previously for DHBV (Weber et al., 1994). In brief, DHBV P *in vitro* translated from plasmid pT7/AMV-pol16 (Weber et al., 1994) served as positive control. An analogous RNDV P construct (pT7/AMV-RNDVpol) was generated by inserting downstream of a T7 promoter a subgenomic fragment of RNDV (nt 1066 to 223) comprising the entire P ORF plus downstream sequences. The YMHD mutant was obtained by site-directed mutagenesis changing RNDV P codon 335 from GAT (Asp) to CAC (His). P proteins were expressed *in vitro* using the TNT Quick Coupled Transcription/Translation System (Promega) according to the manufacturer's instructions. To verify P protein translation, control reactions were performed in the presence of [^{35}S]methionine. For priming assays, P proteins were synthesized in the presence of unlabeled methionine. Subsequently, aliquots of the reaction mixes were either treated with RNase A or left untreated. After addition of priming buffer containing [α-^{32}P]dGTP (3000 Ci/mmol) samples were incubated for 60 min at 37°C (DHBV) or 23°C (RNDV). Priming reactions were terminated by adding SDS protein sample buffer. Samples were subjected to SDS-PAGE followed by detection of labeled protein bands by phospho-imaging.

Ultrastructure of Nackednavirus Capsids

Full-length ORF C of ACNDV (aa 1-174) and a truncated variant lacking the C-terminal nucleic acid binding domain (aa 1-146) were PCR-amplified from a cDNA library of pooled organs from *Ophthalmotilapia ventralis* (Baldo et al., 2011). Both sequences were inserted into the bacterial expression vector pET28a2 (Vogel et al., 2005). After transformation with the respective constructs, *E. coli* were grown overnight in 50 ml starter cultures, transferred to 1 L Terrific Broth and further incubated at 37°C until the cultures reached an OD_{600} of 0.6. Heterologous protein expression was induced by addition of 100 mM IPTG. To reduce sequestration of C proteins in inclusion bodies, the culture medium was supplemented with 3% ethanol (v/v) and cells were subsequently shaken at 25°C for 4 h. After cell lysis in a microfluidizer, bacterial debris was pelleted two times and the supernatants containing soluble capsid particles were filtered through 0.45 μm filter units. Capsid particles were purified by i) pelleting through a 30% (w/w) sucrose cushion (104,000 x g, 12 h, 4°C), ii) sucrose equilibrium density gradient centrifugation (202,000 x g, 12 h, 4°C), and iii) size exclusion chromatography on a Superose-6 column (GE Healthcare). Particle-containing fractions were identified and quality-controlled by SDS-PAGE and Coomassie stain (Figure S3A). A similar attempt to express and purify RNDV capsids did not result in sufficient particle concentrations since the vast majority of RNDV C protein became deposited in insoluble inclusion bodies (a phenomenon well-known e.g. from DHBV C protein).

For cryo-electron microscopy, 2.5 μl aliquots of purified ACNDV capsids were applied to C-Flat 2/2-2C grids glow discharged for 30 s at 20 mA. Grids were blotted for 1-2 s and plunge frozen in liquid ethane using an FEI VitRobot Mark 2. Data acquisition was performed on an FEI Titan Krios (for full-length ACNDV), or an FEI Tecnai F30 Polara (for truncated ACNDV and HBV), both equipped with an FEI Falcon-II direct electron detector and operated at 300 keV.

For full-length ACNDV, 1,548 micrographs were acquired using FEI EPU software package, with a total dose per micrograph of 25 $e^-/Å^2$, a calibrated pixel size of 1.08 Å, and defocus ranging from -1 to -4 μm. Contrast transfer function (CTF) parameters for each micrograph were estimated using CTFFIND3 (Mindell and Grigorieff, 2003). Particle picking was performed using e2boxer from the EMAN2 software package (Tang et al., 2007). The 53,431 picked particles were extracted using RELION 1.2 (Scheres, 2012). After extraction, 2D classification was performed using IMAGIC (van Heel et al., 1996) and RELION 1.2 to select regular, intact particles and remove disrupted particles. 2,547 selected particles were further analysed with RELION 1.2 using a spherical density with diameter of 40 nm as an initial reference and imposing icosahedral symmetry. Two initial classes were generated, the first of which showed features consistent with hepadnavirus morphology. This structure was used as a starting model to refine the full dataset generating a final structure with a measured resolution of 8.0 Å. The structure was sharpened using the relion_postprocess program with a B factor of -500.

For truncated ACNDV, 1,018 micrographs were acquired using Serial EM software package, with a total dose per micrograph of 35 $e^-/Å^2$, a calibrated pixel size of 1.18 Å, and defocus ranging from -1 to -4 μm. Contrast transfer function (CTF) parameters for each micrograph were estimated using CTFFIND4. 781 particles were manually picked and extracted using RELION 1.3 from binned data with pixel size of 2.36 Å. After extraction, 2D classification was performed using RELION 1.3 to select regular, intact particles and remove disrupted particles. 771 selected particles were further analyzed with RELION 1.3 using the ACNDV full-length structure low-pass filtered at 100 Å as initial reference and imposing icosahedral symmetry. Three initial classes were generated, the first of which showed features consistent with ACNDV full-length morphology. The 360 particles assigned to this class were unbinned and further refined against the obtained structure generating a final reconstruction with a measured resolution of 9 Å. The structure was sharpened using the relion_postprocess program with a B factor of -500.

Sequence Alignments

P nucleotide sequences of known ortho- and avihepadnavirus representatives and all viruses discovered in this study were loaded into SeaView (Gouy et al., 2010) which allows switching between nucleotide and protein level and has built-in alignment computation functionalities. In a first step, groups of closely related sequences (for instance the genotypes of human HBV and the woolly monkey hepatitis B virus) were aligned on the protein level using Muscle (Edgar, 2004) with default parameters followed by manual correction of alignment mistakes. In a second step, the group-specific alignments were iteratively joined – from low to high sequence divergence – using Muscle in profile-vs-profile mode followed by manual correction of alignment mistakes (Data S2 provides the

P alignment in fasta format). A PreS/S protein alignment was obtained by *in silico* translating the Pol nucleotide alignment in the PreS/S reading frame. To obtain a C amino acid sequence alignment of hepadna- and nackednaviruses (Data S4), we followed the strategy used for P with the addition that predicted protein secondary structure was taken into account during profile-based alignments. To this end, we used the HHalign program of HHsuite v2.0.16 (Remmert et al., 2012) with the default parameter configuration. HHalign results were incorporated manually into the SeaView-based alignment construction. Graphical representations of sequence alignments were compiled using Jalview (Waterhouse et al., 2009).

Phylogenetic Reconstructions

For all phylogenetic reconstruction analyses we used ProtTest v2.4 (Abascal et al., 2005) to select the best-fitting amino acid substitution model. Uncalibrated Bayesian trees were reconstructed using BEAST v1.8.0 (Drummond et al., 2012) with the substitution model selected by ProtTest and a Yules speciation prior. Two chains were run for five million steps and convergence of the runs was verified using Tracer (Rambaut et al., 2016a). A variable rate molecular clock model with lognormal distribution was applied (Drummond et al., 2006). For details on parameter optimization of the Bayesian phylogenetic model see below and Table S3.

Virus-Host Cophylogeny Testing

To analyze the concordance of the virus and host tree topologies as measure for the degree of coevolution, we applied ParaFit (implemented in the R package APE) as distance–based method (Legendre et al., 2002) and Jane4 as event-based method (Conow et al., 2010). ParaFit statistically tests the extent to which the data fit to the null hypothesis of independent evolution, i.e. random association of viruses and hosts. Global ParaFit *p-value*s – indicating the probability of the null hypothesis to be true – were calculated for the whole virus taxon sampling, and independently for nackednaviruses only and hepadnaviruses only. Two separate runs were performed, one in which the cladistic tree topology was considered solely, and one in which the relative genetic distances of the virus and host lineages were taken into account in addition (results presented in the legend of Figure S6A). Jane4 is a genetic algorithm computing solutions to map a parasite tree onto the host tree with least costs for five types of possible events, i.e. parasite-host cospeciation, parasite duplication, host switch, parasite loss and failure to diverge, respectively. In a first experiment, we used cladistic trees and performed 13 runs with varying cost values for duplication and host switch events. Statistical post-testing demonstrated that the costs for all solutions were below the least costs gained under the assumption of random virus-host associations. Cospeciation events in the nackednaviral and hepadnaviral clade were counted separately in the two largest classes of isomorphic solutions from each of the 13 runs. In a second experiment, we used virus and host trees divided into eight time zones reflecting the relative branch lengths. A single run was performed with default cost values and a population size of 1300 at a generation size of 30, resulting in eight classes of isomorphic solutions which invariantly showed the same pattern of events. The results are presented in the legend of Figure S6A.

Identifying and Reconstructing eAHBV-FRY

To allow for a time-calibration of the viral phylogeny, we recovered sequences of endogenous avihepadnaviral elements (eAHBV) in recently published whole-genome sequence data of birds (Jarvis et al., 2014). To this end, we screened the WGS database at NCBI in tblastn mode using the DHBV C+P protein sequence as query. We retrieved more than 100 hits and selected 35 nearly full-length eAHBV elements which are orthologues of the previously described eZHBV C element from passerine birds (Suh et al., 2013) (corresponding to the endogenous zebra finch hepatitis B virus eZHBVbk, as it was first described by Gilbert and Feschotte, 2010). This element results from an integration near the *FRY* gene and is present in representative genomes of all Neoaves orders (Figure S7A), but absent in the genomes of Galloanserae (Suh et al., 2013). Hence, the element originated from an ancient exogenous avihepadnavirus which invaded the genome of a host bird species in the common ancestry of Neoaves after divergence from galloanserine birds. According to the time-calibration of the phylogeny of modern birds (Jarvis et al., 2014), the endogenization event must have occurred between 89 and 69 mya. P ORF sequences of the 35 eAHBV-FRY elements were reconstructed using MACSE v1.01b (Ranwez et al., 2011) with parameters '-fs 500 -stop 500 -fs lr 15 -stop lr 10'. The extant DHBV and actively transcribed eJHBV P sequences were used as reference to correct indels and stop codons which were introduced after integration into the bird genome.

Time-Calibration of the Viral Phylogeny

Time-calibrated Bayesian trees based on 437 conserved amino acid positions of the P protein alignment were reconstructed using BEAST with the substitution model selected by ProtTest, a calibrated Yules speciation prior, and a variable rate molecular clock model with log-normal distribution. For details on parameter optimization of the Bayesian phylogenetic model see below and Table S3. As control for the obtained topology, uncalibrated Maximum likelihood phylogenetic trees were reconstructed using PhyML v3.0 (Guindon et al., 2010) with the same substitution models as in BEAST and 100 non-parametric bootstraps. We followed two approaches. First, we calibrated the phylogeny by dating the root of the eAHBV-FRY cluster according to the onset of the diversification of Neoaves. Specifically, we used the slightly different calibration dates of 67.4 mya (Prum et al., 2015), 69 mya (Jarvis et al., 2014), and 69.2 mya (Claramunt and Cracraft, 2015) for three independent reconstructions, and a consensus tree was obtained from the joint posterior sets of trees of these three experiments. Second, we performed control calibrations in order to rule out a bias towards

too high age estimates due to a potentially reduced substitution rate after integration of eAHBV-FRY into the Neoavian host genome. To this end, we computed 11 independent time-calibrated trees in addition, each of which was based on the dating of one of seven major diversification events of exogenous hepadnaviruses according to the related host divergence time retrieved from the literature (The Timetree of Life, 2016; Betancur et al., 2013; Bininda-Emonds et al., 2007; Hedges et al., 2015; Wang et al., 2013) (raw data in Table S2).

Bayesian Phylogenetic Model Optimization

Parameters of the uncalibrated and time-calibrated Bayesian phylogenetic models were selected using path sampling (PS) and stepping stone sampling (SS) analysis implemented in BEAST (Baele et al., 2012). The marginal likelihood estimation results are provided in Table S3. We run in total 28 analyses based on our P alignment, testing various combinations of three different, commonly used amino acid substitution models, three different clock models, four different speciation priors, and two different chain lengths. For all priors we used the default distribution in Beauti with the exception of the time-calibrated calculations (analysis 7-10, 17-19, and 26-28 in Table S3) for which we (i) set a uniform prior for the mean clock rate and (ii) used a normally distributed prior with mean of 69.2 and standard deviation of 1.735 for the age of the eAHBV-FRY root according to Jarvis et al. (2014). For the marginal likelihood estimation we used 64 path steps, chain lengths of 275,000 plus 27,500 burn-in, and the default Beta path step distribution in Beauti. Two independent runs were performed for each of the 28 analyses and Tracer was used to assess their proper mixing and convergence. Specifically, we compared the JTT (analysis 1-10), LG (analysis 11-19), and WAG (analysis 20-28) substitution models each with rate heterogeneity across sites modeled through a gamma distribution with four categories (+G). We did not consider substitution models with site rate homogeneity because they never ranked high in ProtTest. Moreover, we compared the strict clock (STRICT), the uncorrelated relaxed clock with log-normal distribution (UCLN), and the uncorrelated relaxed clock with exponential distribution (UCED) models. We also compared the Yule, the Birth-death, the Birth-death with incomplete sampling, and the calibrated Yule speciation priors. Other tree priors were not considered due to their inappropriateness for our family-level analysis. The model parameter configuration chosen for the main analysis – JTT+G with UCLN and Yule speciation prior (uncalibrated model) or calibrated Yule speciation prior (time-calibrated model) – outperformed all other tested parameter configurations in terms of marginal likelihood according to both path sampling and stepping stone sampling. Using this parameter configuration, we additionally run the time-calibrated analysis without data ensuring that the results are not exclusively driven by the prior and with data and for 20 million states (analysis 10) verifying that convergence was reached. The combined traces after burn-in removal of the latter analysis showed an Effective Sample Size (ESS) of 87.8, which we consider sufficient for proper mixing as the two independent chains converged to the same solution.

S Protein Evolution

Weighted substitution rates were calculated for sets of 25 hepadnaviruses, 13 nackednaviruses, 7 caulimoviruses, and 6 retroviruses at each position along the protein-guided codon alignment of P. Non-conserved N-terminal, C-terminal, and spacer regions were excluded from the analysis. The included viruses represent the available species diversity of the four virus families. The weighted substitution frequency at a position was calculated as the weighted average of the number of nucleotide differences at that position between all virus pairs of the family. The weights were calculated as e^{-PED} where *PED* is the pairwise evolutionary distance estimated as the sum of branch lengths separating the two viruses in the P phylogeny. In this way, nucleotide differences between closely related viruses have a higher impact on the weighted substitution frequency than differences between highly divergent virus pairs. For the same alignment relative frequencies of adenine residues were counted for each position of P. The weighted substitution frequencies and the adenine frequencies were plotted separately for first, second, and third codon positions. Curves were smoothed using a sliding window approach with a window size of ten and a shift of two codons.

Hypothetical ancestral sequences (HAS) for the S-corresponding reading frame were generated in MEGA6 (Tamura et al., 2013) based on a nucleotide sequence alignment which comprised the hepadnaviral S ORF and included the sequences of 39 hepadnaviruses, 13 nackednaviruses, 7 caulimoviruses and 6 retroviruses. The topology of the phylogenetic tree shown in Figure 4 of the main text served as guide to yield the most probable ancestral nucleotide state for each alignment position. The following parameters were used for computation: maximum likelihood analysis, GTR model, gamma-distributed rates among sites with invariant sites option. For the separate HAS of hepadnaviruses or nackednaviruses, respectively, we conducted 15 rounds of randomly subsampling each of the major ingroup taxa, inferred HAS during each round in MEGA6, and then manually generated a majority-rule HAS consensus derived from these 15 rounds. In the taxon subsampling for the HAS of the hepadnaviral ancestor, we chose RNDV and SSNDV as outgroups, and randomly picked one representative of orthohepadnaviruses, metahepadnaviruses, avihepadnaviruses, herpetohepadnaviruses, and parahepadnaviruses, respectively. The subsampling for the HAS of the nackednaviral ancestor comprised HBV_ayw_Z35716 and DHBV_AY494851 as outgroups, as well as a random sample from the three major nackednaviral lineages (i.e., RNDV-type, SSNDV-type, and KNDV-Lp-2), respectively. For the reconstruction of the common ancestor of hepadna- and nackednaviruses, the retro- and caulimoviral sequences served as outgroups.

To determine ancestral branch lengths (Figures 7D–7F), we performed a differential phylogenetic tree inference for those parts of RT which overlap with S in hepadnaviruses and are conserved across the ingroup and outgroup viruses. The respective nucleotide alignment was translated to protein level in the P and S-corresponding reading frames and Bayesian trees were reconstructed for both protein sequences using BEAST (Drummond et al., 2012) with the substitution model selected by ProtTest (Abascal et al.,

2005) and a Yules speciation prior. Lengths of the branches (measured as amino acid substitutions per site) were determined in FigTree (Rambaut, 2016) in order to calculate the branch length ratio between the S- and P-frame-based trees.

DATA AVAILABILITY

Annotated genome sequences of all viruses described in this study in gb-format: Data S1. P protein alignment of viral sequences used in this study in fasta format: Data S2. EM structures of full-length (aa1-174) and truncated (aa1-146) ACNDV capsids, respectively, have been deposited in the Electron Microscopy Data Bank with accession numbers EMDB: EMD-3822 and EMD-3823.

Cell Host & Microbe, Volume *22*

Supplemental Information

Deciphering the Origin and Evolution of Hepatitis B Viruses by Means of a Family of Non-enveloped Fish Viruses

Chris Lauber, Stefan Seitz, Simone Mattei, Alexander Suh, Jürgen Beck, Jennifer Herstein, Jacob Börold, Walter Salzburger, Lars Kaderali, John A.G. Briggs, and Ralf Bartenschlager

SUPPLEMENTAL INFORMATION

Figure S1, related to Figure 1. Genome maps of novel HBV-related fish viruses

Figure S2, related to Figure 1. Genome maps of new tetrapod hepatitis B viruses

Figure S3, related to Figure 3. Morphology and ultrastructure of heterologously expressed nackednavirus capsids

Figure S4, related to Figure 4. Uncalibrated phylogenetic trees of P

Figure S5, related to Figure 4. Uncalibrated Bayesian phylogenetic trees of P including outgroups, and of C

Figure S6, related to Figures 5 and 6. Correlation of the hepadnaviral phylogeny with the host phylogeny

Figure S7, related to Figure 4. Phylogenetic relationship of endogenous avihepadnaviruses (eAHBV-*FRY*), time-calibrated tree based on endogenous snake hepatitis B virus 1 (eSnHBV-1), and time-calibrated subtree of HBV genotype isolates from humans and apes

Table S1, related to Figure 1. Synopsis of novel HBV-related viruses described in this study

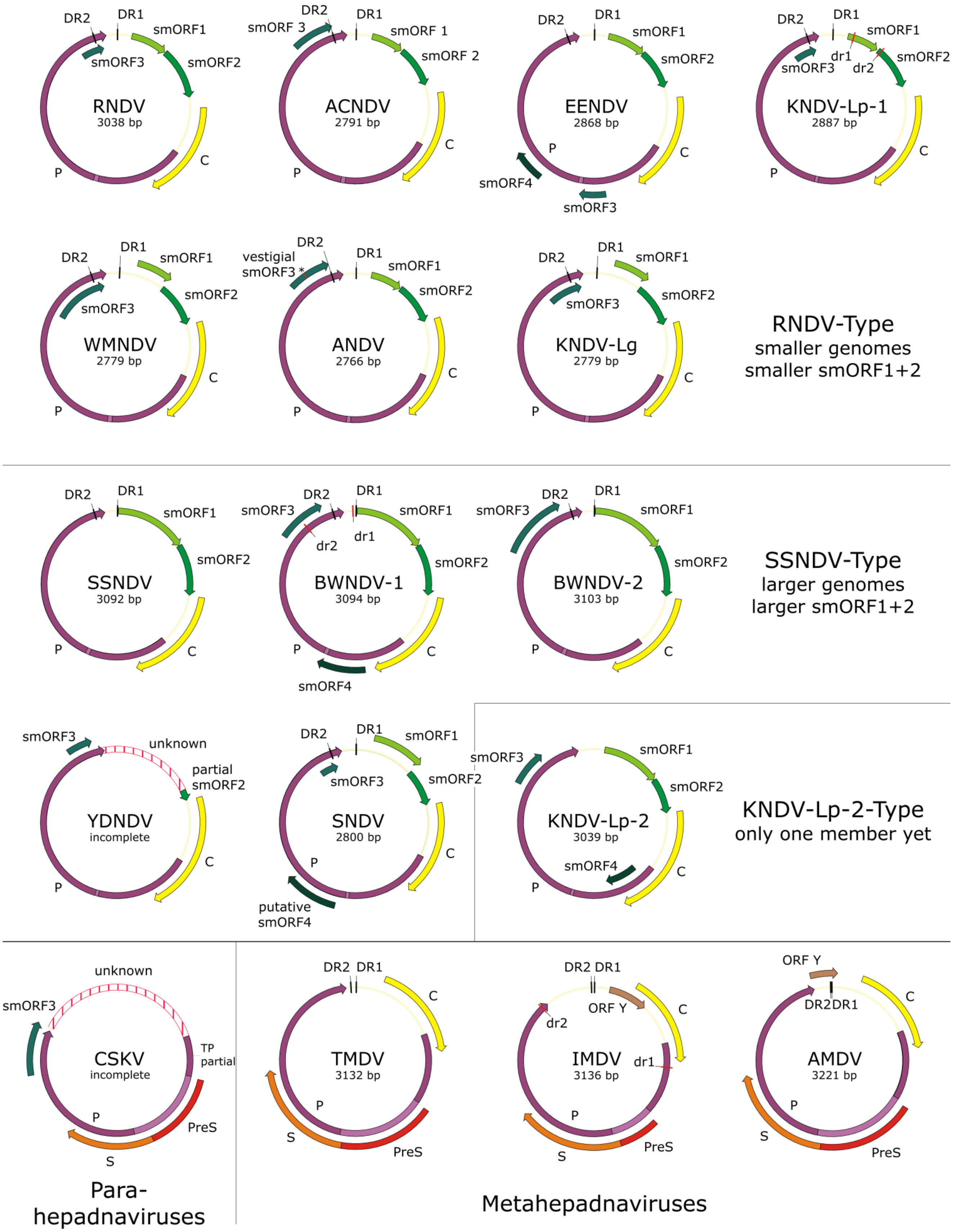

DR2
DR1
smORF1
smORF2
smORF3
RNDV
3038 bp
P
C
smORF 3
smORF 1
smORF 2
ACNDV
2791 bp
EENDV
2868 bp
smORF4
KNDV-Lp-1
2887 bp
dr1
dr2
WMNDV
2779 bp
vestigial
smORF3 *
ANDV
2766 bp
KNDV-Lg
2779 bp
RNDV-Type
smaller genomes
smaller smORF1+2
SSNDV
3092 bp
BWNDV-1
3094 bp
BWNDV-2
3103 bp
SSNDV-Type
larger genomes
larger smORF1+2
unknown
partial
smORF2
YDNDV
incomplete
SNDV
2800 bp
putative
smORF4
KNDV-Lp-2
3039 bp
KNDV-Lp-2-Type
only one member yet
CSKV
incomplete
TP
partial
PreS
S
TMDV
3132 bp
ORF Y
IMDV
3136 bp
AMDV
3221 bp
Para-
hepadnaviruses
Metahepadnaviruses

Figure S1, related to Figure 1. Genome maps of HBV-related fish viruses

The 5'-ends of direct repeat DR1 were defined as genome start coordinates. Several viral genomes contain an additional pair of direct repeats (dr1, dr2) with uncertain functionality. None of the viruses described here has a PreC ORF typical for ortho- and avihepadnaviruses. Rows 1 to 4: nackednaviruses; row 5: hepadnaviruses. ACNDV: African cichlid nackednavirus (a fragment of the genome of this nackednavirus was previously described as ACHBV – African cichlid hepatitis B virus – by Hahn et al., 2015); AMDV: Astatotilapia metahepadnavirus; ANDV: Astatotilapia nackednavirus; BWNDV-1 and -2: “Baby whale” (Mormyrid) nackednaviruses; CSKV: Coho salmon kidney virus; EENDV: European eel nackednavirus; IMDV: Icefish metahepadnavirus; KNDV-Lg: Killifish nackednavirus from Lucania goodei; KNDV-Lp-1 and -2: Killifish nackednaviruses from Lucania parva; RNDV: Rockfish nackednavirus; SNDV: Stickleback nackednavirus; SSNDV: Sockeye salmon nackednavirus; TMDV: Tetra metahepadnavirus; WMNDV: Western mosquitofish nackednavirus; YNDV: Yellow drum nackednavirus.

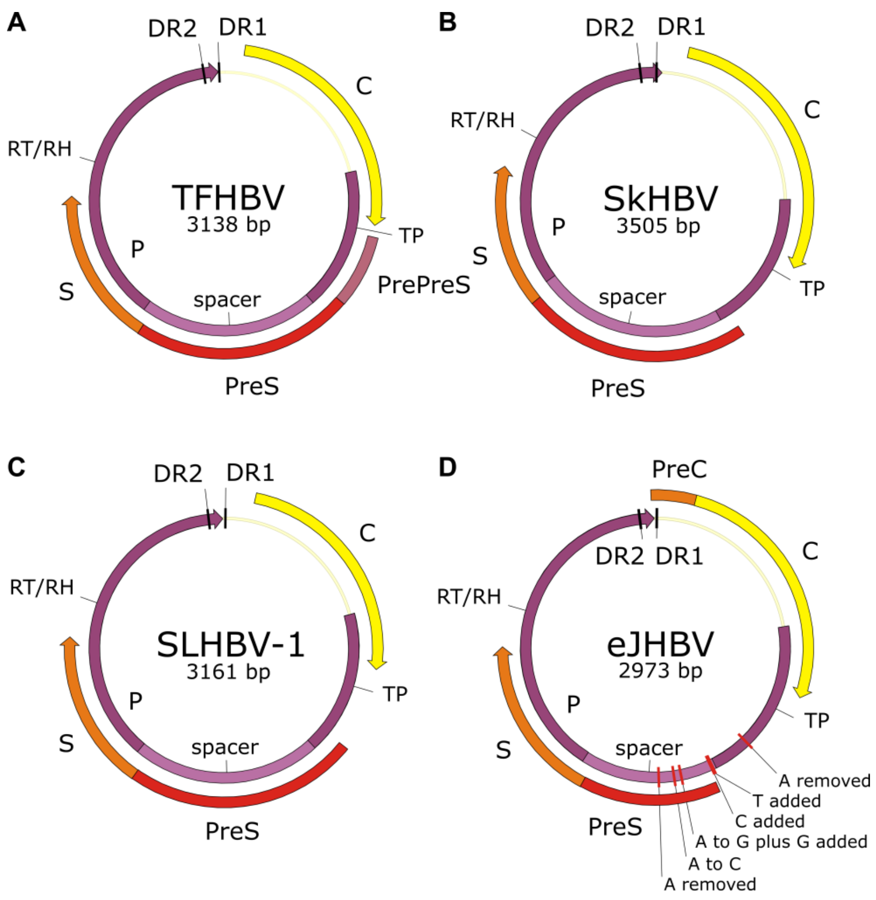

Figure S2, related to Figure 1. Genome maps of tetrapod hepatitis B viruses

(A) Tibetan frog hepatitis B virus (TFHBV). The same virus genome was independently discovered by Dill et al. (2016).

(B) Skink hepatitis B virus (SkHBV).

(C) Spiny lizard hepatitis B virus (SLHBV-1).

(D) Endogenous junco hepatitis B virus (eJHBV). Corrections for frameshift mutations and premature stop codons in eJHBV as indicated.

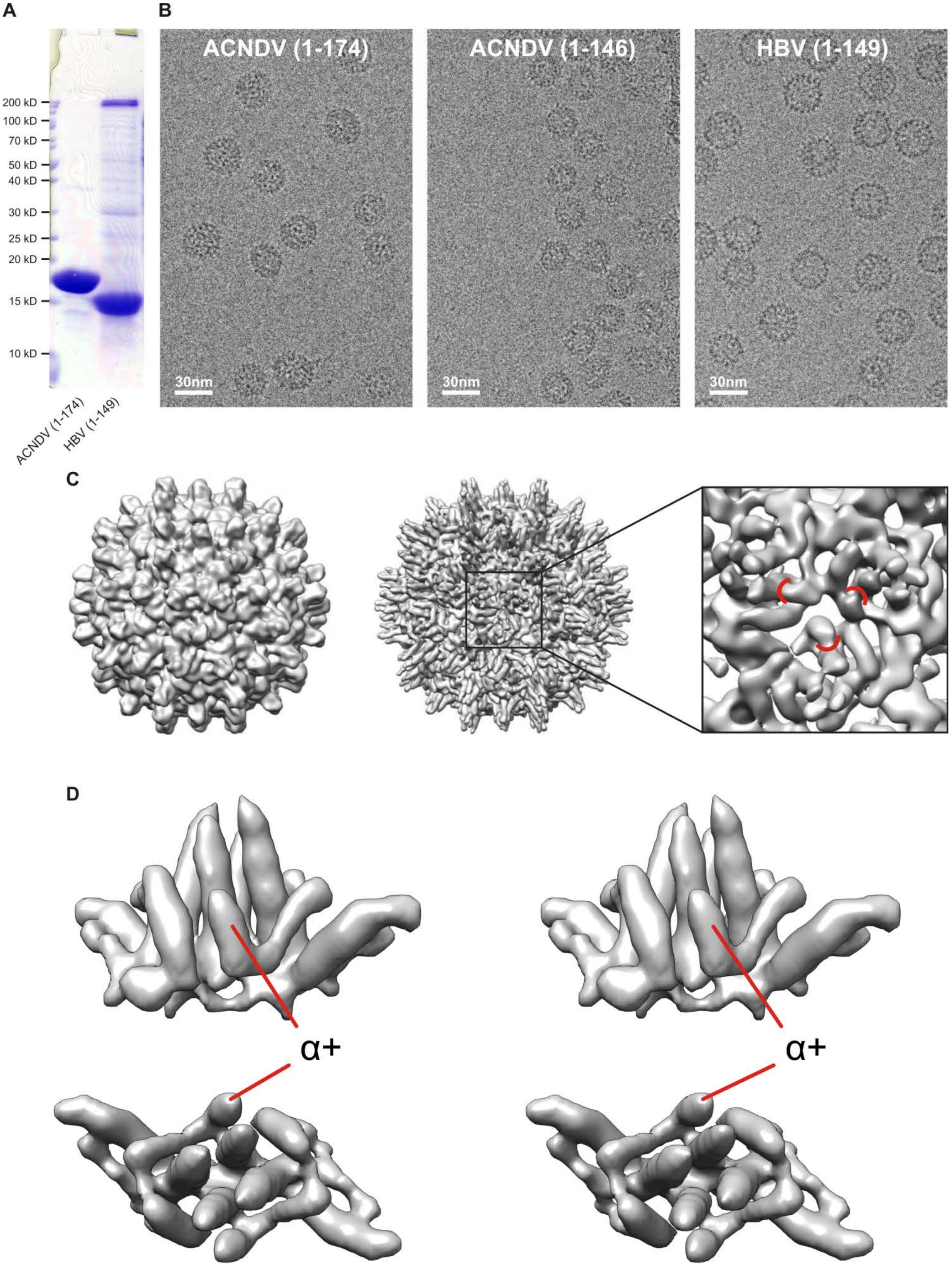
A
B
200 kD
100 kD
70 kD
50 kD
40 kD
30 kD
25 kD
20 kD
15 kD
10 kD
ACNDV (1-174)
HBV (1-149)
ACNDV (1-174)
ACNDV (1-146)
HBV (1-149)
30nm
30nm
30nm
C
D
α+
α+

Figure S3, related to Figure 3. Morphology and ultrastructure of heterologously expressed nackednavirus capsids

(A) SDS-PAGE and Coomassie-stain of highly purified ACNDV capsids consisting of full-length C protein (174 aa). Control: partially purified capsids built of truncated HBV C (aa 1-149).

(B) Cryo-electron micrographs of purified capsid particles self-assembled from full-length ACNDV C (aa 1-174), truncated ACNDV C (aa 1-146) and truncated HBV C proteins (aa 1-149), respectively. Full-length and truncated ACNDV capsids do not display the strict dimorphism known from HBV. They are more variable in shape than HBV capsid particles. The vast majority of ACNDV particles are small, and we were not able to detect a class of regular particles with T=4 icosahedral symmetry. Nonetheless, we do not rule out that some irregular particles might have local areas following a T=4 pattern. Full-length and truncated ACNDV particles appear similar, indicating that deletion of the C-terminal residues 147-174, including the nucleic acid binding domain, has no obvious influence on the shape or size distribution.

(C) Cryo-EM map of C-terminally truncated ACNDV (aa 1-146), filtered at 9 Å. Comparison with the structure of full-length ACNDV (Figure 3) indicates no substantial change in structure of the capsid scaffold. Notably, the reappearance of the additional helices sealing the holes at the local (pseudo-)three-fold axes independently corroborates that these helices represent the N-termini of ACNDV C protein molecules.

(D) Cross-eye stereo views onto an ACNDV C protein dimer. Top panel: side view; bottom panel: top view. α+: additional N-terminal α-helices not found in hepadnaviruses.

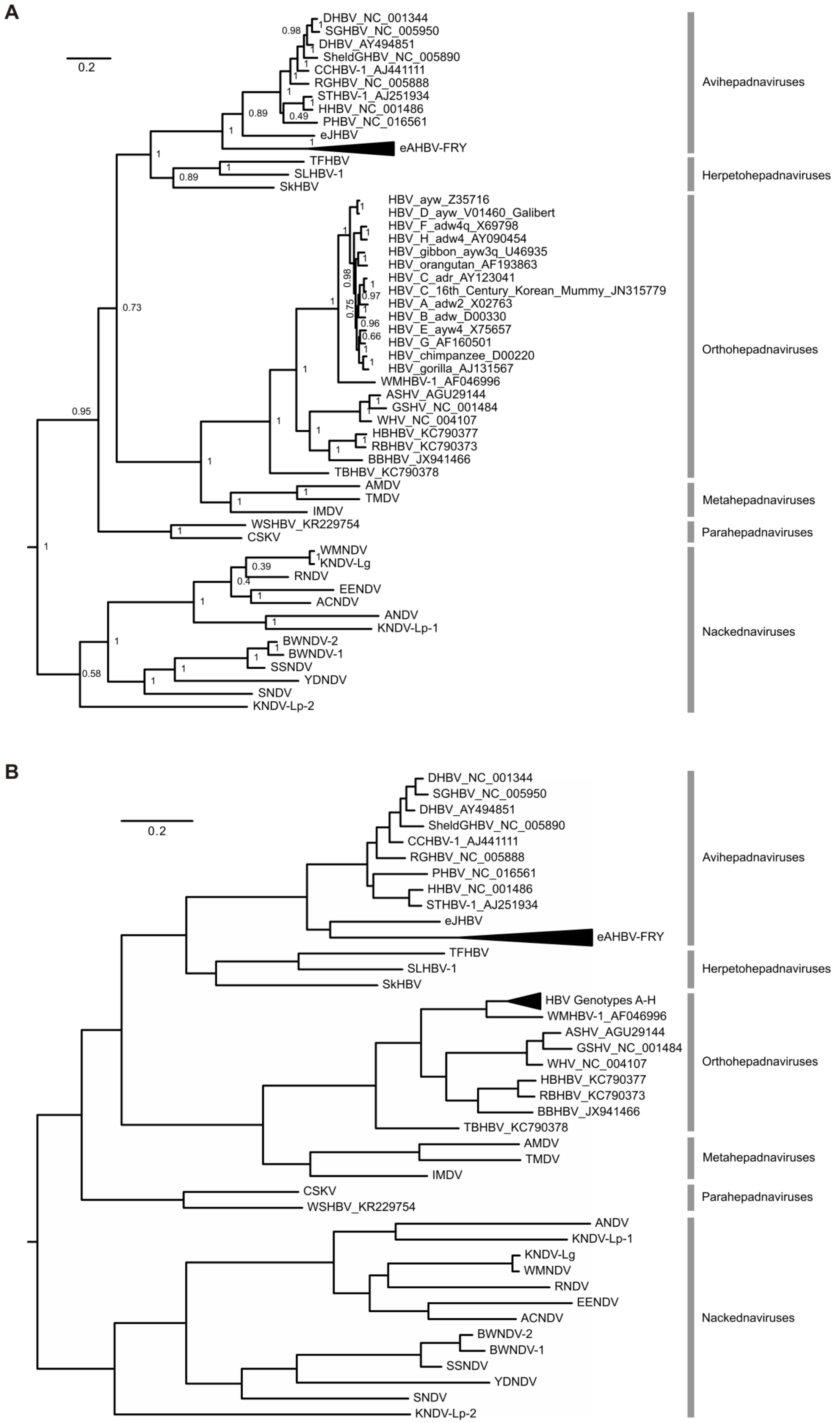
A
0.2
DHBV_NC_001344
SGHBV_NC_005950
DHBV_AY494851
SheldGHBV_NC_005890
CCHBV-1_AJ441111
RGHBV_NC_005888
STHBV-1_AJ251934
HHBV_NC_001486
PHBV_NC_016561
eJHBV
eAHBV-FRY
TFHBV
SLHBV-1
SkHBV
HBV_ayw_Z35716
HBV_D_ayw_V01460_Galibert
HBV_F_adw4q_X69798
HBV_H_adw4_AY090454
HBV_gibbon_ayw3q_U46935
HBV_orangutan_AF193863
HBV_C_adr_AY123041
HBV_C_16th_Century_Korean_Mummy_JN315779
HBV_A_adw2_X02763
HBV_B_adw_D00330
HBV_E_ayw4_X75657
HBV_G_AF160501
HBV_chimpanzee_D00220
HBV_gorilla_AJ131567
WMHBV-1_AF046996
ASHV_AGU29144
GSHV_NC_001484
WHV_NC_004107
HBHBV_KC790377
RBHBV_KC790373
BBHBV_JX941466
TBHBV_KC790378
AMDV
TMDV
IMDV
WSHBV_KR229754
CSKV
WMNDV
KNDV-Lg
RNDV
EENDV
ACNDV
ANDV
KNDV-Lp-1
BWNDV-2
BWNDV-1
SSNDV
YDNDV
SNDV
KNDV-Lp-2
Avihepadnaviruses
Herpetohepadnaviruses
Orthohepadnaviruses
Metahepadnaviruses
Parahepadnaviruses
Nackednaviruses
B
0.2
DHBV_NC_001344
SGHBV_NC_005950
DHBV_AY494851
SheldGHBV_NC_005890
CCHBV-1_AJ441111
RGHBV_NC_005888
PHBV_NC_016561
HHBV_NC_001486
STHBV-1_AJ251934
eJHBV
eAHBV-FRY
TFHBV
SLHBV-1
SkHBV
HBV Genotypes A-H
WMHBV-1_AF046996
ASHV_AGU29144
GSHV_NC_001484
WHV_NC_004107
HBHBV_KC790377
RBHBV_KC790373
BBHBV_JX941466
TBHBV_KC790378
AMDV
TMDV
IMDV
CSKV
WSHBV_KR229754
ANDV
KNDV-Lp-1
KNDV-Lg
WMNDV
RNDV
EENDV
ACNDV
BWNDV-2
BWNDV-1
SSNDV
YDNDV
SNDV
KNDV-Lp-2
Avihepadnaviruses
Herpetohepadnaviruses
Orthohepadnaviruses
Metahepadnaviruses
Parahepadnaviruses
Nackednaviruses

Figure S4, related to Figure 4. Uncalibrated phylogenetic trees of P

(A) Uncalibrated Bayesian tree based on conserved regions of the P protein (437 amino acid positions underlined with light grey bars in the P protein alignment in Supplemental Data File S3). The clade of hepatitis B virus genotypes A-H, including isolates from gibbons and great apes, is shown expanded. The eAHBV-FRY cluster is shown collapsed (see Figure S7A for an expanded subtree). Scale bar: 0.2 amino acid substitutions per site.

(B) Maximum likelihood phylogenetic tree of P. The tree is based on the same dataset as in Figures 4 and S4A. JTT + G4 + F substitution model selected by ProtTest (Abascal et al., 2005). The root was determined with TempEst (Rambaut et al., 2016b). Nackedna- and hepadnaviruses demarcate as well-separated sister clades, thus independently confirming the results of the Bayesian approach. Scale bar: 0.2 amino acid substitutions per site.

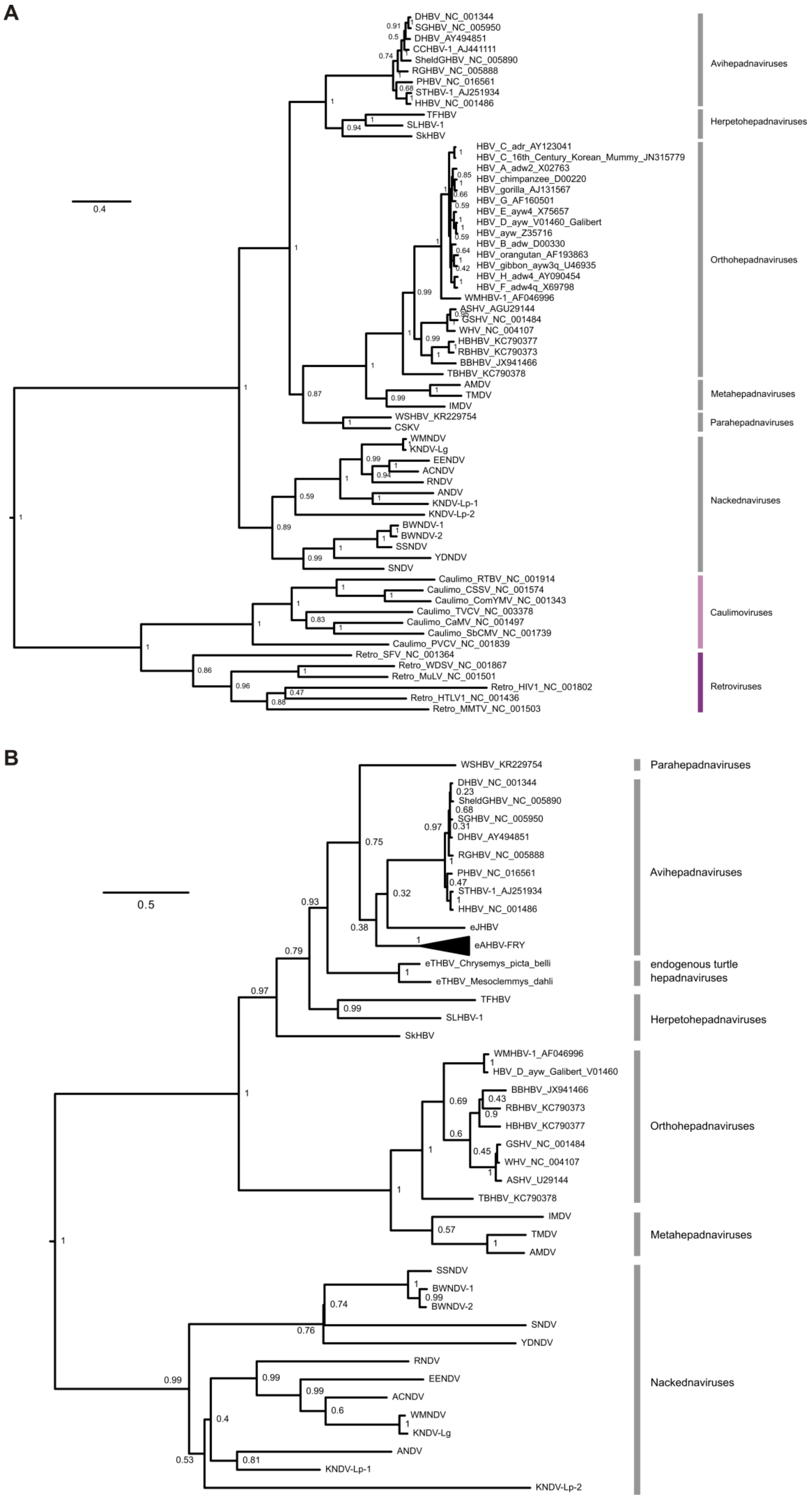

A
0.4
DHBV_NC_001344
SGHBV_NC_005950
DHBV_AY494851
CCHBV-1_AJ441111
SheldGHBV_NC_005890
RGHBV_NC_005888
PHBV_NC_016561
STHBV-1_AJ251934
HHBV_NC_001486
TFHBV
SLHBV-1
SkHBV
HBV_C_adr_AY123041
HBV_C_16th_Century_Korean_Mummy_JN315779
HBV_A_adw2_X02763
HBV_chimpanzee_D00220
HBV_gorilla_AJ131567
HBV_G_AF160501
HBV_E_ayw4_X75657
HBV_D_ayw_V01460_Galibert
HBV_ayw_Z35716
HBV_B_adw_D00330
HBV_orangutan_AF193863
HBV_gibbon_ayw3q_U46935
HBV_H_adw4_AY090454
HBV_F_adw4q_X69798
WMHBV-1_AF046996
ASHV_AGU29144
GSHV_NC_001484
WHV_NC_004107
HBHBV_KC790377
RBHBV_KC790373
BBHBV_JX941466
TBHBV_KC790378
AMDV
TMDV
IMDV
WSHBV_KR229754
CSKV
WMNDV
KNDV-Lg
EENDV
ACNDV
RNDV
ANDV
KNDV-Lp-1
KNDV-Lp-2
BWNDV-1
BWNDV-2
SSNDV
YDNDV
SNDV
Caulimo_RTBV_NC_001914
Caulimo_CSSV_NC_001574
Caulimo_ComYMV_NC_001343
Caulimo_TVCV_NC_003378
Caulimo_CaMV_NC_001497
Caulimo_SbCMV_NC_001739
Caulimo_PVCV_NC_001839
Retro_SFV_NC_001364
Retro_WDSV_NC_001867
Retro_MuLV_NC_001501
Retro_HIV1_NC_001802
Retro_HTLV1_NC_001436
Retro_MMTV_NC_001503
Avihepadnaviruses
Herpetohepadnaviruses
Orthohepadnaviruses
Metahepadnaviruses
Parahepadnaviruses
Nackednaviruses
Caulimoviruses
Retroviruses
B
0.5
WSHBV_KR229754
DHBV_NC_001344
SheldGHBV_NC_005890
SGHBV_NC_005950
DHBV_AY494851
RGHBV_NC_005888
PHBV_NC_016561
STHBV-1_AJ251934
HHBV_NC_001486
eJHBV
eAHBV-FRY
eTHBV_Chrysemys_picta_belli
eTHBV_Mesoclemmys_dahli
TFHBV
SLHBV-1
SkHBV
WMHBV-1_AF046996
HBV_D_ayw_Galibert_V01460
BBHBV_JX941466
RBHBV_KC790373
HBHBV_KC790377
GSHV_NC_001484
WHV_NC_004107
ASHV_U29144
TBHBV_KC790378
IMDV
TMDV
AMDV
SSNDV
BWNDV-1
BWNDV-2
SNDV
YDNDV
RNDV
EENDV
ACNDV
WMNDV
KNDV-Lg
ANDV
KNDV-Lp-1
KNDV-Lp-2
Parahepadnaviruses
Avihepadnaviruses
endogenous turtle hepadnaviruses
Herpetohepadnaviruses
Orthohepadnaviruses
Metahepadnaviruses
Nackednaviruses

Figure S5, related to Figure 4. Uncalibrated Bayesian phylogenetic trees of P including outgroups, and of C

(A) This P tree is based on conserved parts of RT and RH shared with caulimo- and retroviruses as outgroups (see P protein alignment in Supplemental Data File S3). Independent corroboration of the rooting of the phylogenetic inferences shown in Figures 4 and S4. Numbers at branching points show posterior probability support values. Scale bar: 0.4 amino acid substitutions per site.

(B) Bayesian phylogenetic tree of C based on 45 conserved amino acid residues of the C protein alignment shown in Supplemental Data File S4. JTT + G4 substitution model selected by ProtTest. Scale bar: 0.5 amino acid substitutions per site. The similarity of this tree with those for P (Figures 4, S4 and S5A) provides evidence that there is no significant variation of the phylogenies between genes. There is one prominent exception from the major concordance between the C- and P-based phylogenies: In the C-based tree the piscine parahepadnavirus WSHBV clusters with avihepadnaviruses, irrespective of inference method and substitution model used for tree inference. We assume this to be a case of homoplasy due to convergent evolution not reflecting the true phylogeny, since the C protein of WSHBV belongs to the plesiomorphic short type of C proteins while avi- and herpetohepadnaviruses share elongated C proteins as derived trait (Supplemental Data File S4). The other, minor differences, like the position of SkHBV, are likely caused by the limited number of conserved C protein residues resulting in a lower power to resolve isolated branches compared to the P proteins.

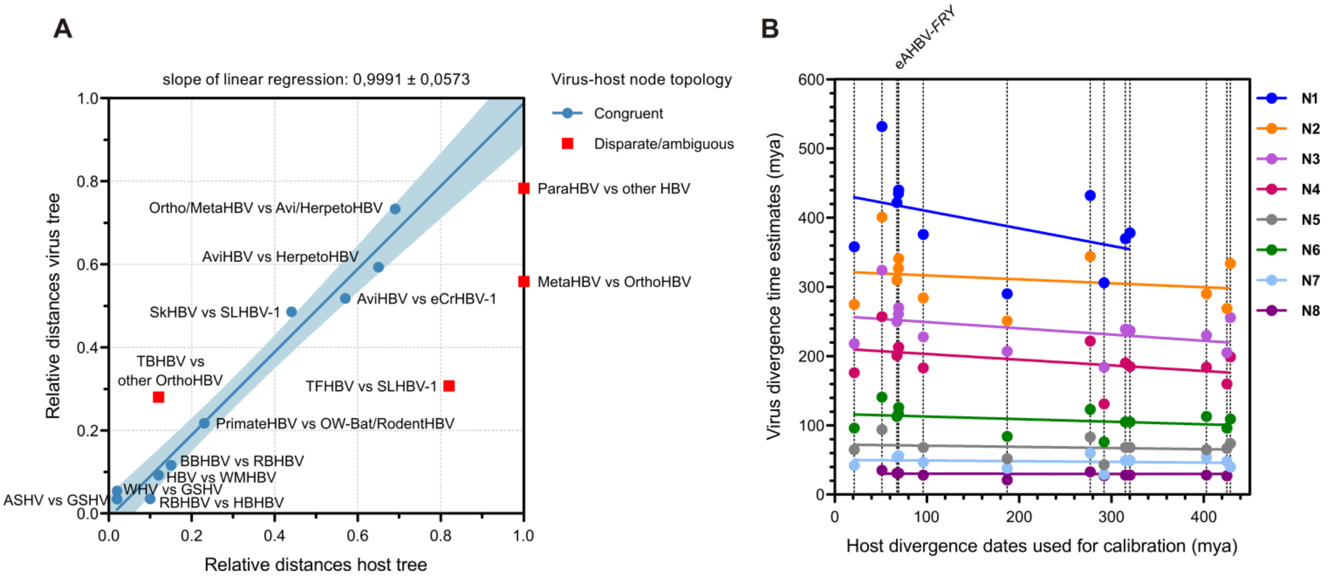

Figure S6, related to Figures 5 and 6. Correlation of the hepadnaviral phylogeny with the host phylogeny

(A) Matching of the relative distances between hepadnavirus and host nodes with congruent topology in the uncalibrated ultrametric trees shown in the tanglegram in Figure 5 (blue dots). Linear regression: R^2 = 0.9743. Slope = 0.9991 ± 0.0571 SD, indicating that also the roots of both trees represent a virus-host codivergence event (nackednaviral clade & actinopterygians vs. hepadnaviral clade & sarcorpterygians, including tetrapods). Red squares depict the relative distances of the major nodes with disparate virus-host topology in the tanglegram (Figure 5), as well as the one ambiguous node (piscine parahepadnaviruses vs. other hepadnaviruses) which – by mere cladistic topology – could represent an alternative cospeciation event associated with the split between actinopterygians and sarcopterygians. The evident double-mismatch of topology *and* relative branch length between the virus and host trees for these viral nodes makes it unlikely that they result from cospeciation events with their current host lineages. In order to corroborate the virus-host evolutionary patterns independently, we additionally performed statistical cophylogeny tests using ParaFit (Legendre

et al., 2002) and Jane4 (Conow et al., 2010). Based on the cladistic topology, global ParaFit *p*-values – indicating the probability of random virus-host associations, i.e. independent evolution of hosts and viruses – were <0.0001, 0.0015, and 0.3032, respectively, for the whole virus taxon sampling, hepadnaviruses only, and nackednaviruses only. When taking into account the relative branch lengths of the virus and host trees, the global ParaFit *p*-values were <0.0001 for the whole virus taxon sampling, 0.0133 for hepadnaviruses only, and 0.4025 for nackednaviruses only. Based on the cladistic topology, Jane4 predicted on average 3.54 of 12 speciation events in the nackednaviral lineage to result from virus-host cospeciation (frequency: 0.295), and 12.81 of 20 in the hepadnaviral lineage (frequency: 0.640), respectively. In a second experiment accounting for the relative branch lengths, Jane4 invariantly detected the same putative cospeciation events in all resulting solutions, three of them occurring on the nackednaviral side (frequency: 0.25) and 12 of them on the hepadnaviral side (frequency: 0.6).

(B) Virus divergence time estimates resulting from 14 independent tree inferences, each calibrated on one single node in the hepadnaviral phylogeny. Dots on each vertical dashed line represent viral node age estimates from an individual time-scaled tree. The three calibrations based on the root age of eAHBV-FRY are indicated. Node numberings as in Figures 5 and 6. Independently obtained age estimates for each node and related linear regression analyses are in the same color. The lack of a correlation (slopes of linear regressions not significantly deviating from zero) indicates that the retrieved divergence time estimates for any individual node are independent from the ages of the nodes used for tree calibration. This observation rules out a bias caused by substitution saturation which would lead to an systematic underestimation of divergence times when calibrating trees by use of young (more terminal)

nodes and an overestimation of divergence times when calibrating trees by use of ancient (more basal) nodes (van Tuinen and Torres, 2015).

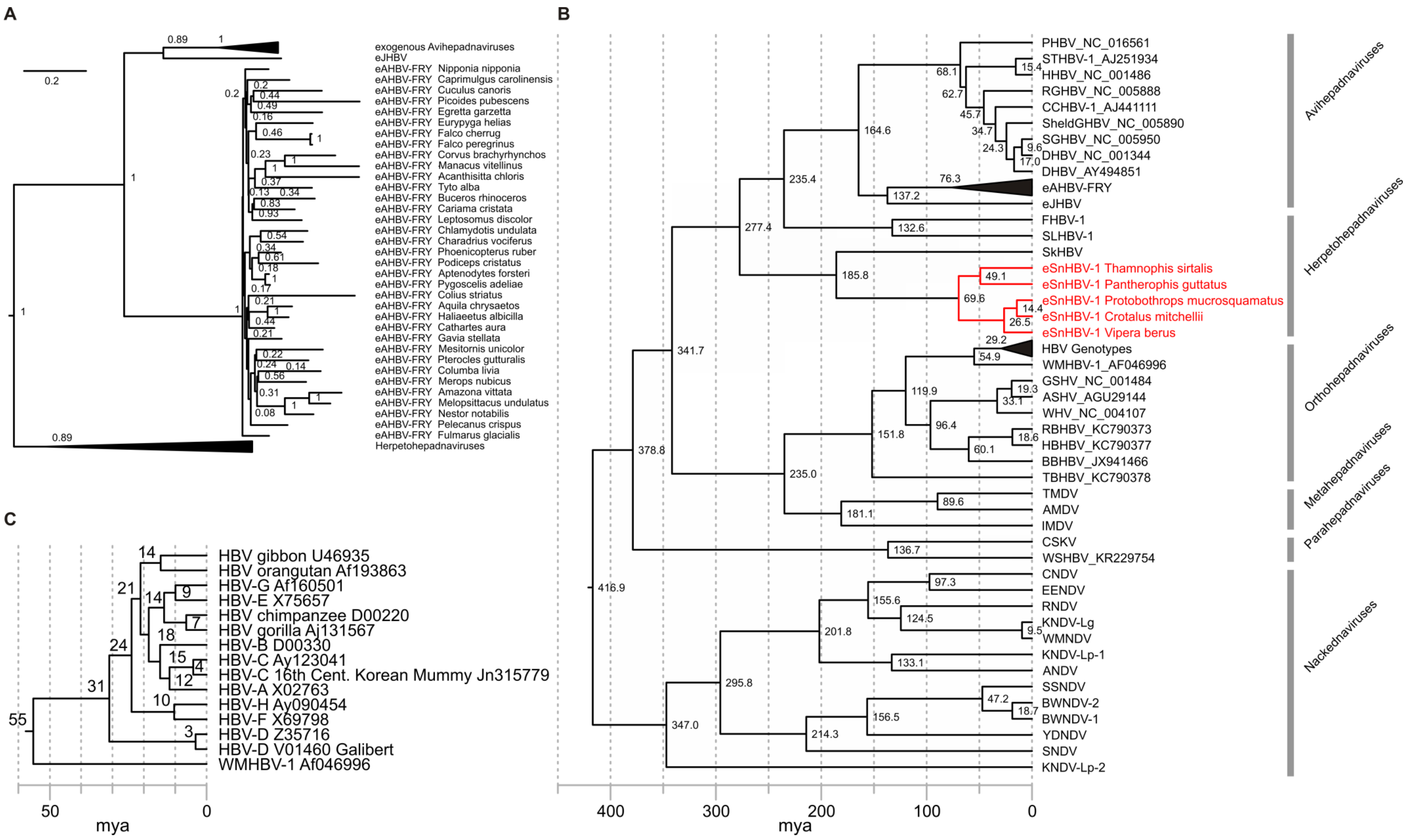
A
B
C
exogenous Avihepadnaviruses
eJHBV
eAHBV-FRY Nipponia nippona
eAHBV-FRY Caprimulgus carolinensis
eAHBV-FRY Cuculus canoris
eAHBV-FRY Picoides pubescens
eAHBV-FRY Egretta garzetta
eAHBV-FRY Eurypyga helias
eAHBV-FRY Falco cherrug
eAHBV-FRY Falco peregrinus
eAHBV-FRY Corvus brachyrhynchos
eAHBV-FRY Manacus vitellinus
eAHBV-FRY Acanthisitta chloris
eAHBV-FRY Tyto alba
eAHBV-FRY Buceros rhinoceros
eAHBV-FRY Cariama cristata
eAHBV-FRY Leptosomus discolor
eAHBV-FRY Chlamydotis undulata
eAHBV-FRY Charadrius vociferus
eAHBV-FRY Phoenicopterus ruber
eAHBV-FRY Podiceps cristatus
eAHBV-FRY Aptenodytes forsteri
eAHBV-FRY Pygoscelis adeliae
eAHBV-FRY Colius striatus
eAHBV-FRY Aquila chrysaetos
eAHBV-FRY Haliaeetus albicilla
eAHBV-FRY Cathartes aura
eAHBV-FRY Gavia stellata
eAHBV-FRY Mesitornis unicolor
eAHBV-FRY Pterocles gutturalis
eAHBV-FRY Columba livia
eAHBV-FRY Merops nubicus
eAHBV-FRY Amazona vittata
eAHBV-FRY Melopsittacus undulatus
eAHBV-FRY Nestor notabilis
eAHBV-FRY Pelecanus crispus
eAHBV-FRY Fulmarus glacialis
Herpetohepadnaviruses
PHBV_NC_016561
STHBV-1_AJ251934
HHBV_NC_001486
RGHBV_NC_005888
CCHBV-1_AJ441111
SheldGHBV_NC_005890
SGHBV_NC_005950
DHBV_NC_001344
DHBV_AY494851
eAHBV-FRY
eJHBV
FHBV-1
SLHBV-1
SkHBV
eSnHBV-1 Thamnophis sirtalis
eSnHBV-1 Pantherophis guttatus
eSnHBV-1 Protobothrops mucrosquamatus
eSnHBV-1 Crotalus mitchellii
eSnHBV-1 Vipera berus
HBV Genotypes
WMHBV-1_AF046996
GSHV_NC_001484
ASHV_AGU29144
WHV_NC_004107
RBHBV_KC790373
HBHBV_KC790377
BBHBV_JX941466
TBHBV_KC790378
TMDV
AMDV
IMDV
CSKV
WSHBV_KR229754
CNDV
EENDV
RNDV
KNDV-Lg
WMNDV
KNDV-Lp-1
ANDV
SSNDV
BWNDV-2
BWNDV-1
YDNDV
SNDV
KNDV-Lp-2
Avihepadnaviruses
Herpetohepadnaviruses
Orthohepadnaviruses
Metahepadnaviruses
Parahepadnaviruses
Nackednaviruses
mya
HBV gibbon U46935
HBV orangutan Af193863
HBV-G Af160501
HBV-E X75657
HBV chimpanzee D00220
HBV gorilla Aj131567
HBV-B D00330
HBV-C Ay123041
HBV-C 16th Cent. Korean Mummy Jn315779
HBV-A X02763
HBV-H Ay090454
HBV-F X69798
HBV-D Z35716
HBV-D V01460 Galibert
WMHBV-1 Af046996
mya

Figure S7, related to Figure 4. Phylogenetic relationship of endogenous avihepadnaviruses (eAHBV-*FRY*), time-calibrated tree based on endogenous snake hepatitis B virus 1 (eSnHBV-1), and time-calibrated subtree of HBV genotype isolates from humans and apes

(A) We extracted and reconstructed a set of 35 endogenous avihepadnaviral (eAHBV) P protein sequences from whole-genome sequencing data of birds (Zhang et al., 2014). These belong to an orthologous integration of an almost full-length viral genome near the *FRY* gene, and hence descended from a single genome invasion event. Since eAHBV-FRY is present in all currently sequenced genomes of Neoaves, but absent in the genomes of Galloanserae (Suh et al., 2013), the integration must have occurred in the ancestry of Neoaves after divergence from galloanserine birds, i.e. between 89 and 69 mya (Jarvis et al., 2014). Depicted is a subtree of the uncalibrated Bayesian phylogenetic tree in Figure S4A. The eAHBV-FRY cluster is shown expanded and extant exogenous avihepadnaviruses and herpetohepadnaviruses are included as outgroups. The explosive diversification of the eAHBV-FRY isolates closely resembles the rapid adaptive radiation of Neoaves which started 69—67 mya (Jarvis et al., 2014; Prum et al., 2015; Claramunt and Cracraft, 2015). Consequently, we assume concomitant diversification and used this age for dating the root of eAHBV-*FRY* to time-calibrate the viral phylogeny (see Figures 6 and S6B). Numbers at branching points show posterior probability support values. Scale bar: 0.2 amino acid substitutions per site.

(B) Time-calibrated Bayesian tree based on P sequences of eSnHBV-1 from five member species of the snake superfamily Colubroidea (marked in red). Numbers indicate node age estimates in mya. eSnHBV-1 was first detected in the genome of the speckled rattlesnake (*Crotalus mitchellii*) (Gilbert et al., 2014; Suh et al., 2014). We assembled and reconstructed additional, almost full-length eSnHBV-1 P sequences from the European adder (*Vipera berus*),

the brown spotted pit viper (*Protobothrops mucrosquamatus*), the corn snake (*Pantherophis guttatus*) and the common garter snake (*Thamnophis sirtalis*). 417 aa positions of the P protein alignment (Supplemental Data File S2) were utilized for reconstructing time-calibrated phylogenies. We computed a consensus tree from two independent Bayesian phylogenetic inferences (JTT+G4 model, relaxed molecular clock with log-normal distribution, Yules speciation prior), in which we dated the root of the eSnHBV-1 P protein sequences using published age estimates for the most recent common ancestor of Colubroidea of 61 mya (Pyron and Burbrink, 2012; Zheng and Wiens, 2016) or 77 mya (Castoe et al., 2009; Kyriazi et al., 2013), respectively. The retrieved node age estimates of this consensus tree match very well with those obtained in the calibrations based eAHBV-FRY (Figure 4). For example, the separation of nackedna- and hepadnaviruses was estimated to have occurred 417 mya and the root age of eAHBV-FRY was determined as 76 mya (compare to Figures 4 and 6).

(C) Time-calibrated subtree of the main tree in Figure 4 with the HBV genotype isolates from humans and apes shown expanded. Numbers indicate node age estimates in mya. Woolly monkey hepatitis B virus (WMHBV) from the New world monkey *Lagothrix lagotricha* included as outgroup.

Table S1, related to Figure 1. Synopsis of novel HBV-related viruses described in this study

Virus	Host; common name (scientific name)	Seq. type[a]	Source[b]	Tissue/organ	Remarks
**Nackednaviruses RNDV-type**					
RNDV	Tiger rockfish (*Sebastes nigrocinctus*)	WGS	AUPR01188533	Fin	Further hits in unpublished SRA
ACNDV	African cichlid (*Ophthalmotilapia ventralis*)	TS	gb|JL559376	Pooled organs	Further hits: gb|JL576735.1; SRX078329
ANDV	Astatotilapia (*Astatotilapia sp.*)	WGS	ERX240954	Unknown	Bioproject with 433 WGS experiments of individual fish
EENDV	European eel (*Anguilla anguilla*)	TS	SRX700630	Olfactory epithelium	
KNDV-Lg	Bluefin killifish (*Lucania goodei*)	TS	SRX340220	Pooled organs	Gill, eye, fin, testis, ovary, brain
KNDV-Lp-1	Rainwater killifish (*Lucania parva*)	TS	SRX340836	Pooled organs	PolyA-tail identified; gill, eye, fin, testis, ovary, brain
WMNDV	Western mosquitofish (*Gambusia affinis*)	TS	SRX376926	Ovary	
**Nackednaviruses SSNDV-type**					
SSNDV	Sockeye salmon (*Oncorhynchus nerka*)	TS	SRX265393	Pooled organs	PolyA-tail identified; heart, liver gonad, muscle, olfact. bulb
BWNDV-1	Baby whale (*Brienomyrus brachyistius*)	TS	SRX553136	Muscle	
BWNDV-2	Baby whale (*Brienomyrus brachyistius*)	TS	SRX573075	Electric organ	PolyA-tail identified
SNDV	Three-spined stickleback (*G. aculeatus*)	WGS	SRX1037831	Unknown	
YDNDV	Yellow drum (*Nibea albiflora*)	TS	SRX367575	Unknown	
**Nackednaviruses KNDV-Lp-2-type**					
KNDV-Lp-2	Rainwater killifish (*Lucania parva*)	TS	SRX340853	Pooled organs	Gill, eye, fin, testis, ovary, brain
**Parahepadnaviruses**					
CSKV	Coho salmon (*Oncorhynchus kisutch*)	TS	SRX1037831	Kidney	Not in liver and spleen => Coho salmon kidney virus
**Metahepadnaviruses**					
AMDV	Astatotilapia (*Astatotilapia sp.*)	WGS	ERX674915	Unknown	Bioproject with 433 WGS experiments of individual fish
IMDV	Crocodile icefish (*Chionodraco hamatus*)	TS	SRX145766	Muscle	
TMDV	Mexican tetra (*Astyanax mexicanus*)	TS	SRX229523	Eyes-surface	
**Herpetohepadnaviruses**					
TFHBV	Tibetan frog (*Nanorana parkeri*)	WGS	PRJNA243398	Muscle	High sequence coverage; probably high titer viremia
SkHBV	Skink (*Saproscincus basiliscus*)	TS	SRX213382	Unknown	
SLHBV-1	Spiny lizard (*Sceloporus adleri*)	WGS	SRX542351	Liver	pooled with heart, muscle
**Avihepadnaviruses**					
eJHBV	Dark-eyed junco (*Junco hyemalis*)	TS	PRJNA158927	Pooled organs	14 organs including liver

[a]Sequencing type: WGS: whole-genome shotgun sequencing; TS: transcriptome shotgun sequencing. [b]Source annotations: ERX and SRX are SRA experiments at NCBI; PRJNA are Bioprojects at NCBI; all others are genbank accession numbers.
